# Genomic Diversification, Structural Plasticity, and Hybridization in *Leishmania (Viannia) braziliensis*

**DOI:** 10.3389/fcimb.2020.582192

**Published:** 2020-10-16

**Authors:** Luz H. Patino, Marina Muñoz, Lissa Cruz-Saavedra, Carlos Muskus, Juan David Ramírez

**Affiliations:** ^1^ Grupo de Investigaciones Microbiológicas-UR (GIMUR), Departamento de Biología, Facultad de Ciencias Naturales, Universidad del Rosario, Bogotá, Colombia; ^2^ Programa de Estudio y Control de Enfermedades Tropicales (PECET), Facultad de Medicina, Universidad de Antioquia, Medellín, Colombia

**Keywords:** DNA-seq, genomic variability, copy number variations, single-nucleotide polymorphisms, *Leishmania braziliensis*

## Abstract

*Leishmania (Viannia) braziliensis* is an important *Leishmania* species circulating in several Central and South American countries. Among *Leishmania* species circulating in Brazil, Argentina and Colombia, *L. braziliensis* has the highest genomic variability. However, genomic variability at the whole genome level has been only studied in Brazilian and Peruvian isolates; to date, no Colombian isolates have been studied. Considering that in Colombia, *L. braziliensis* is a species with great clinical and therapeutic relevance, as well as the role of genetic variability in the epidemiology of leishmaniasis, we analyzed and evaluated intraspecific genomic variability of *L. braziliensis* from Colombian and Bolivian isolates and compared them with Brazilian isolates. Twenty-one genomes were analyzed, six from Colombian patients, one from a Bolivian patient, and 14 Brazilian isolates downloaded from public databases. The results obtained of Phylogenomic analysis showed the existence of four well-supported clades, which evidenced intraspecific variability. The whole-genome analysis revealed structural variations in the somy, mainly in the Brazilian genomes (clade 1 and clade 3), low copy number variations, and a moderate number of single-nucleotide polymorphisms (SNPs) in all genomes analyzed. Interestingly, the genomes belonging to clades 2 and 3 from Colombia and Brazil, respectively, were characterized by low heterozygosity (~90% of SNP loci were homozygous) and regions suggestive of loss of heterozygosity (LOH). Additionally, we observed the drastic whole genome loss of heterozygosity and possible hybridization events in one genome belonging to clade 4. Unique/shared SNPs between and within the four clades were identified, revealing the importance of some of them in biological processes of *L. braziliensis*. Our analyses demonstrate high genomic variability of *L. braziliensis* in different regions of South America, mainly in Colombia and suggest that this species exhibits striking genomic diversity and a capacity of genomic hybridization; additionally, this is the first study to report whole-genome sequences of Colombian *L. braziliensis* isolates.

## Introduction

*Leishmania (Viannia) braziliensis* is the most important *Leishmania* species associated with cases of cutaneous leishmaniasis and muco-cutaneous leishmaniasis in several Central and South American countries (Ramirez et al., 2016; Patino et al., 2017). This species is characterized by its ability to cause distinct forms of tegumentary leishmaniasis in humans (Jirmanus et al., 2012; Meireles et al., 2017) and animals (De Oliveira et al., 2013; Brilhante et al., 2019), and also for its variable infectivity, virulence, and response to anti-leishmanial therapy (Rego et al., 2018; Patino et al., 2019a). In addition, it is considered a zoonotic parasite circulating in a wide range of mammalian and vector host species (Kuhls et al., 2013; Roque and Jansen, 2014), characteristics that can contribute to the generation and maintenance of genetic diversity within the species (Cupolillo et al., 2003; Bruna et al., 2019).

Several nuclear and mitochondrial DNA markers as well as different molecular techniques have been used with the purpose to evaluate inter- and intra-specific genetic variability of New/Old world *Leishmania* species, such as *L. major*, *L.infantum, L. donovani*, *L. tropica*, *L. braziliensis*, *L. peruviana*, *L. guyanensis*, and *L. panamensis* (Cupolillo et al., 2003; Nolder et al., 2007; Kuhls et al., 2013; Restrepo et al., 2013; Marco et al., 2015; Restrepo et al., 2015; Fotouhi-Ardakani et al., 2016; Cysne-Finkelstein et al., 2018; Quaresma et al., 2018; Banu et al., 2019). This has suggested high intra-specific variability, especially for *L. braziliensis* from Brazil (Marlow et al., 2014), Argentina (Marco et al., 2015) and Colombia (Herrera et al., 2017). This variability is probably related to sand fly vector(s) and/or animal reservoir(s) involved in transmission cycles (Cupolillo et al., 2003) and with different clinical manifestations (Quaresma et al., 2018; Rego et al., 2018). However, few studies have examined this variability in whole genomes of New-World *Leishmania* species, such as *L. braziliensis* (Valdivia et al., 2015; Urrea et al., 2018; Bruna et al., 2019; Restrepo et al., 2019), because most of the research has focused on revealing the genome diversity of Old-World *Leishmania* species (Downing et al., 2011; Imamura et al., 2016; Ghouila et al., 2017; Franssen et al., 2020).

Two recent studies have used whole genome sequencing to reveal the genetic variability of *L. braziliensis*. One of them, published by Bruna et al. (2019) highlighted the tremendous genetic variability (~95,000–~131,000 SNPs) in 10 clinical isolates from forested and urbanized environments of Brazil and the existence of three distinct phylogenetic groups including one isolate from a forested environment that was characterized by moderate aneuploidy and reduced heterozygosity. The other study by Broeck et al. (2020) investigated the nuclear and mitochondrial genomes of *L. braziliensis* isolates from Peru and demonstrated genetic diversification and subsequent hybridization, this study highlights the origin of Andean/Amazonian *Leishmania* species (*L. peruviana* and *L. braziliensis*) and describes a possible meiotic recombination event between them, with uniparental inheritance of maxicircles but biparental inheritance of minicircles, which may be crucial for survival of the parasite in the wild. Considering the close relatedness between *L. braziliensis* and *L. peruviana*, some authors have conducted genomic comparative analysis, which have identified a great number of interspecific SNP/indel differences between them as well as the presence of different gene and chromosome copy number variations supporting the classification of both organisms as closely related but distinct species (Valdivia et al., 2015; Broeck et al., 2020).

Despite these findings, knowledge about the genomic variability of *L. braziliensis* in other South American regions, such as Colombia, is limited. Different studies describe a high frequency of this species in Colombia; this being more frequent in rural (Perez-Franco et al., 2016; Patino et al., 2017) compared with urban populations (Ramirez et al., 2016; Ovalle-Bracho et al., 2019). In addition, Colombia has a large number of sand fly vectors and animal reservoirs, which are involved in the transmission cycle of the parasite (Valderrama-Ardila et al., 2010; Ferro et al., 2015). Considering the public health importance of leishmaniasis caused by *L. braziliensis* in Colombia and the role of genetic variability of the parasite in the epidemiology of the disease, the objective of this study was to perform a comprehensive analysis of whole genome sequencing (DNA-seq) to evaluate phylogenomic relationships among six Colombian clinical isolates and one Bolivian clinical isolate. We also determined phylogenomic relationships and genomic plasticity among these isolates and 14 publicly available *L. braziliensis* genomes from Brazil in terms of genome plasticity (chromosomes/genes) and single point mutations.

## Methods

### Ethics Statement

All procedures were approved by the Ethics committee of the Universidad de Antioquia (number VRI3445/2010) in accordance with resolution number 36836. The patients included into the study read and signed the informed consent.

### Population of Study

Twenty-one whole genomes were analyzed in this study, including six from Colombian clinical isolates and one Bolivian clinical isolate from patients who were attending in the ‘Programa de Estudio y Control de Enfermedades Tropicales (PECET), Medellín-Colombia’. Also, 14 *L. braziliensis* genomic sequences reads, from Brazilian isolates publicly available from the DDBJ/ENA/GenBank database (http://www.ebi.ac.uk/ena) under accession codes PRJNA292004 (Alves-Ferreira et al., 2015) and PRJNA475480 (Bruna et al., 2019) were downloaded for respective comparison. The dataset was downloaded using the tools described in: https://github.com/EnzoAndree/getENA. The seven isolates included in this study were obtained from patients from different regions of Colombia and one from Bolivia, with nodular or ulcerative cutaneous lesions. The sampling was performed before patients underwent anti-Leishmanial therapy.

### Parasite Culture and Identification

The parasites obtained from six Colombian clinical isolates and the Bolivian isolate were cultured axenically in Schneider’s insect medium, which was supplemented with 10% (v/v) fetal bovine serum; the cultures were maintained at 26°C with 5% CO_2_ as describe previously (Patino et al., 2020). Once the parasites were in late logarithmic growth phase, were recovered by centrifugation and submitted to DNA extraction. High Pure PCR Template Preparation Kit (Roche Life Science) was used for DNA extraction in accordance with the manufacturer’s instructions. Later the concentration, quality and integrity of DNA were determined. To verify the quality of DNA, each sample was divided in two groups, the first used for species identification and the second for whole-genome sequencing.

Cytochrome b proteins (*Cytb*) and heat shock protein (*Hsp70*) were the genes selected to species identification through Sanger sequencing, as has been described previously (Ramirez et al., 2016; Patino et al., 2017). The amplification products were purified with EXOSAP (Affymetrix, USA) and sequenced using the dideoxy-terminal method in an automated capillary sequencer (AB3730; Applied Biosystem). Subsequently, sequences were subjected to a BLASTn similarity search for *Leishmania* sequences deposited in GenBank (Ramirez et al., 2016). Those isolates identified as *L. braziliensis* were selected for whole-genome sequencing.

### Genomic Sequencing

HiSeq X-Ten system (Illumina) was used to sequence the whole-genome DNA, later mate-paired libraries were built and finally subjected to paired-end sequencing (2 × 150-bp read length producing a median coverage at least of 40x per sample). Reads with adapter contamination, >10% uncertain nucleotides, or >50% low-quality nucleotides (base quality < 5) were discarded (Yan et al., 2013).

### DNA Mapping

The paired-end Illumina reads of six Colombian clinical isolates, one Bolivian clinical isolate and of the fourteen Brazilian genomes (data downloaded from European Nucleotide Archive) were mapped to the reference MHOMBR75/M2904_2019 *L. braziliensis* genome sequence assembly (http://tritrypdb.org) using the SMALT program (version 0.7.4) (www.sanger.ac.uk/resources/software/smalt/). Exhaustive search option (−x and −y of 0.8), a sliding step of 3 and a reference hash index of 13 bases were the parameters using during the mapping. To prevent the mapping of non-*Leishmania* reads to the reference sequences, we used an identity threshold of y = 0.8. SAMtools (version 0.1.18) and Picard (version 1.85) were used to no-mapping read exclusion, sorting, file merging and elimination of PCR duplicates (Imamura et al., 2016). The reads corresponding to the mitochondrial genome were extracted and assembled under the same pipeline described for nuclear genome but using maxicircle sequence from novel long read assembly of *L. braziliensis* M2904 (available in tritrypDB).

### Phylogenomic Analysis

SNPs alignments from whole nuclear and mitochondrial (maxicircle) genomes unphased and phased (see “Phasing of heterozygous SNP sites” section), were used to evaluate the phylogenomic relationship among *L. braziliensis* isolates and between each sequence generated by each isolate during the phasing. FastTree double precision version 2.1.10. (Price et al., 2009) was used to build a based-maximum-likelihood phylogenetic tree. The robustness of the nodes was evaluated using the Bootstrap method (BT, with 1,000 replicates). The obtained tree was visualized using the interactive tool Interactive Tree Of Life V4 (http://itol.embl.de) (Letunic and Bork, 2019). To detect recombination signatures in the analyzed genomes, phylogenetic networks were built in SplitsTree5 (Huson and Bryant, 2006) using the Neighbor-Net method. We included MHOMBR75/M2904_2019 *L. braziliensis* as genome sequence (REF) and used as outgroup the *L. guyanensis* genome assembly from European Nucleotide Archive accession number SRR8179913 (Lguy_SRR8179913) (http://www.ebi.ac.uk/ena) and the *L. panamensis* genome assembly from TriTrypDB http://tritrypdb.org: LPPSC1 (LpW).

### Somy Analysis and Gene Copy Number Variations

The chromosomal somy was estimated calculating initially the median read depth of each chromosome (di). Positions with read depth of >1 standard deviation were removed and the di recalculated. Later, we calculated the median depth (dm) of whole genome (35 chromosomes) and the somy (S-value) of each chromosome, for that we used the formula previously described S = 3 × di/dm (Patino et al., 2019a). The ranges of somy (mono-di-tri-tetra and penta somy) were defined, as previously described (Dumetz et al., 2017).

To evaluate the gene copy number variations (CNVs), we calculated and related the average haploid depth per gene without somy effect (d_HG_) and the full cell depth with somy effect (d_FG_) using the formula: (*d_FG_ = S × d_HG_*). The statistical significance used in this study was set at a z-score cutoff of >2 and an adjusted p-value (Student’s t-test) of <0.05. Heatmaps were created using the Heatmap3 package in R (Zhao et al., 2014). Finally, we used the Gene Ontology enrichment analyses from TriTrypDB tools (http://tritrypdb.org) to evaluate the genes with CNVs. *P-values* were adjusted for multiple testing with Benjamini-Hochberg method with a false-discovery rate (FDR) of <0.05. The GO terms were submitted to REVIGO (Supek et al., 2011).

### SNP Estimations and Analysis

Initially, the reads were mapped to the reference MHOMBR75/M2904_2019 *L. braziliensis* genome sequence assembly using the SMALT program (version 0.7.4), different options of this program were used to search random mapping of multiple hit reads and obtained optimal alignments. The merging and sorting of bam files and marking duplicated reads were implemented with the Picard program (version 1.85) (http://broadinstitute.github.io/picard/) as described previously (Dumetz et al., 2017). The SNPs were called with the population-based Unified Genotyper method in the Genome Analysis Toolkit (GATK) (version 3.4; https://software.broadinstitute.org/gatk/), where SNPs were called among all the samples simultaneously. Later, we realigned around indels to remove these and retrieved only the SNPs. GATK Variant Filtration was used to filter Low-quality SNPs, according to the following criteria: QD < 2.0 || MQ < 40 || FS > 60.0 || ReadPosRankSum < −8.0. The SNP quality cutoff was set at 3000. Later, the Integrative Genomic Viewer program (IGV_2_3_47) was used to visualize all SNPs identified and the SnpEff program (version v4.1) to classify the SNPs based on their functional impact (Dumetz et al., 2017). Once the SNPs were independently detected, these were extracted from nuclear and mitochondrial alignments of *L. braziliensis* genomes using snp-sites program (Page et al., 2016). Based on the total number of SNPs and using snp-distans program (https://github.com/tseemann/snp-dists), we generated a pairwise distance matrix; the results obtained were graphically represented. Lastly, we selected the SNPs that have a potential effect on gene function (high and moderate impact); from this selection, we identified the unique/shared SNPs between and within the clades and between the Colombian genomes, the data were included in an Excel matrix, which was used to perform the comparative analysis. Finally, to measure nucleotide diversity (π), we used the DnaSP software v.5.0.

#### Heterozygosity/Homozygosity Analysis

From allele frequency estimation data, we determined the Homozygous and heterozygous variants. Allele shifts of < 0.33 or > 0.66 were considered as homozygous variants while allele shifts between 0.33 and 0.66 as heterozygous variants (Tihon et al., 2017). Once homozygous and heterozygous variants were identified, these were counted using an Excel matrix. Later, the heterozygosity/homozygosity for each chromosome from each sample was extracted and calculated using the variant call formats through a Perl script. The identified heterozygous and homozygous SNPs for each chromosome per sample were plotted using the packages: Stringr, Ape, and Phangorn in R (Paradis et al., 2004; Schliep et al., 2019; Wickham, 2019).

#### Phasing of Heterozygous SNP Sites

Heterozygous SNPs were phased using BEAGLE v5.1 (https://faculty.washington.edu/browning/beagle/beagle.html) over 30 iterations. The algorithm also imputes missing genotypes from identity-by-state segments found in the data (Schwabl et al., 2019). A custom Perl script was used to retrieve the sequence from the phased region and replace consensus bases with SNPs, generating thus two sequences for each isolate (denote as A and B), substituting base predictions for each haplotype into each sequence. This was conducted with whole genome data and by chromosome.

## Results

### Identification of *Leishmania* Species

**Figure 1:**
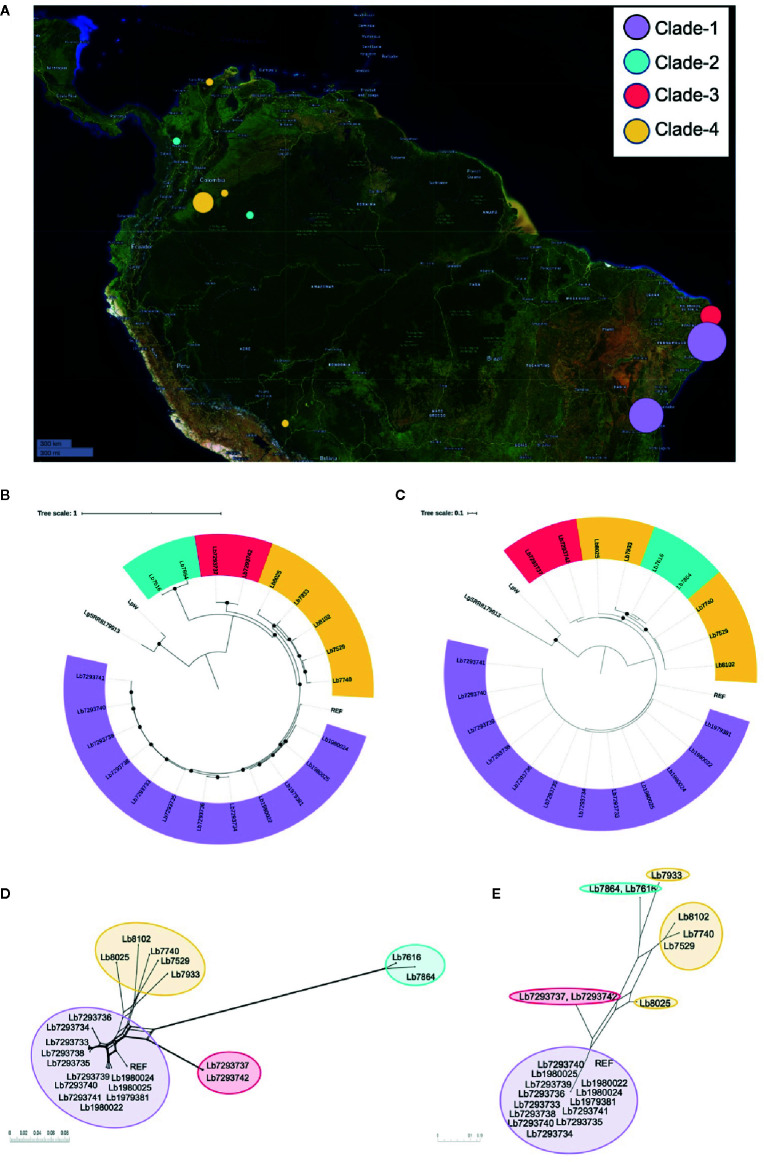
Geographical distribution of genomes included in the study and *Leishmania braziliensis* phylogenomic relationships among nuclear and mitochondrial genomes. **(A)** Geographical location of the *L. braziliensis* genomes analyzed. The map was built using Microreact online tool (https://microreact.org/showcase) based on the GPS coordinates of each isolation (QGIS Geographic Information System, Open Source Geospatial Foundation Project, http://qgis.osgeo.org). Phylogenomic analysis based on whole nuclear genome **(B)** and mitochondrial genome: maxicircle **(C)**. MHOM/BR75/M2904_2019 *L. braziliensis* (REF) was included as reference genome and Lguy_SRR8179913 (*L. guyanensis*) and LpW (*L. panamensis*) were used as outgroup. Black dots represent well-supported nodes (Bootstrap ≥ 90). **(D, E)** represent the phylogenetic network (Neighbor-Net) constructed in SplitsTree 5, based on nuclear and mitochondrial (maxicircle) genomes respectively.

The **Table 1** and **Figure 1A** describe the clinical characteristics and geographical distribution of the Colombian/Bolivian isolates analyzed in this study. The amplification and Sanger sequencing of *Cytb* and *Hsp70* genes identified that the isolates herein included corresponded to *L. braziliensis* (**Supplementary Table S1**).

**Table 1:** Clinical characteristic of each Colombian clinical isolate analyzed in the study.

Database accession number	Genome ID	Gender	Age (years)	Origin	Number of lesions	Lesion type	Treatment
ERS4385934	Lb7616	Male	20	Vaupés	3	Ulcerative	Glucantime intralesional
ERS4385933	Lb7864	Male	16	Antioquia	1	Nodular	ND
ERS4385937	Lb7529	Male	22	Meta	1	Ulcerative	Glucantime systemic
ERS4385938	Lb7740	Male	57	Meta	1	Ulcerative	Glucantime intralesional
ERS4385939	Lb7933	Male	31	Bolivia	1	Nodular	Thermotherapy
ERS4385935	Lb8025	Male	36	Meta	1	Ulcerative	Miltefosine/thermotherapy
ERS4385936	Lb8102	Male	49	Guajira	9	Ulcerative	Glucantime systemic

ND, No Data.

#### Phylogenomic Analysis

Two alignments were used to conduct phylogenomic analyses for 21 genome sequences. The first corresponded to single nucleotide polymorphisms (SNPs) from nuclear genome (1,320,593 nucleotide positions). The second corresponded to SNPs from mitochondrial genomes (167 nucleotide positions). For both cases MHOM/BR75/M2904_2019 *L. braziliensis* genome as the reference and *L. guyanensis* (Lguy_SRR8179913) and *L. panamensis* LP-PSC1 (LpW) genomes as outgroups. The approximately-maximum-likelihood phylogenetic trees built in FastTree double precision version 2.1.10 (Price et al., 2009) allowed initially to confirm that all the genomes evaluated were closely related to the reference genome and evidencing that the genomes analyzed here corresponding to *L. braziliensis* (**Figures 1B, C**). In addition, tree topologies (**Figures 1B, C**) describe the presence of four subpopulations clustered in well-supported nodes (with bootstrap ≥ 90.0). Clade 1 (highlighted in purple) included most of the Brazilian genomes (Lb1979381, Lb1980022, Lb1980024, Lb1980025, Lb7293733 Lb7293734, Lb7293735, Lb7293736, Lb7293738, Lb7293739, Lb7293740, and Lb7293741), clade 2 (highlighted in sky blue) two Colombian genomes (Lb7616 and Lb7864), clade 3 (highlighted in red) the remaining Brazilian genomes (Lb7293737 and Lb7293742), and clade 4 (highlighted in yellow) included the remaining Colombian genomes (Lb7529, Lb7740, Lb8025, and Lb8102) and the Bolivian genome (Lb7933). Interestingly, the two Colombian genomes belong to clade 2, collected from Vaupés and Antioquia were closer to the Brazilian genomes belonging to clade 3 than to genomes of clade 4, which included the other Colombian genomes collected from other regions of the country (Meta and Guajira). These findings were additionally supported by phylogenetic tree topologies obtained in SplitsTree5 (Huson and Bryant, 2006) (using neighbor-net method), where the members of these Clades were consistently clustered together and the four divergent between them (**Figures 1D, E**). The profiles were consistent between nuclear and mitochondrial analyses except for three remarkable events of clustering change that were defined as swapping events. These events were present in two relatively distant sub-clades within clade 4 (sub-clade 1 formed by Lb8025 and Lb7933 and sub-clade 2 by Lb7529, Lb7740, and Lb8102) (**Supplementary Figure S1**). Remarkably, one of the isolates that formed a possible subclade (Lb7933) was from Bolivia. Finally, we did not observe any relationship between the clustering patterns with the clinical or therapeutic characteristics of each genome analyzed.

### Evaluation of Chromosome and Gene CNVs

**Figure 2:**
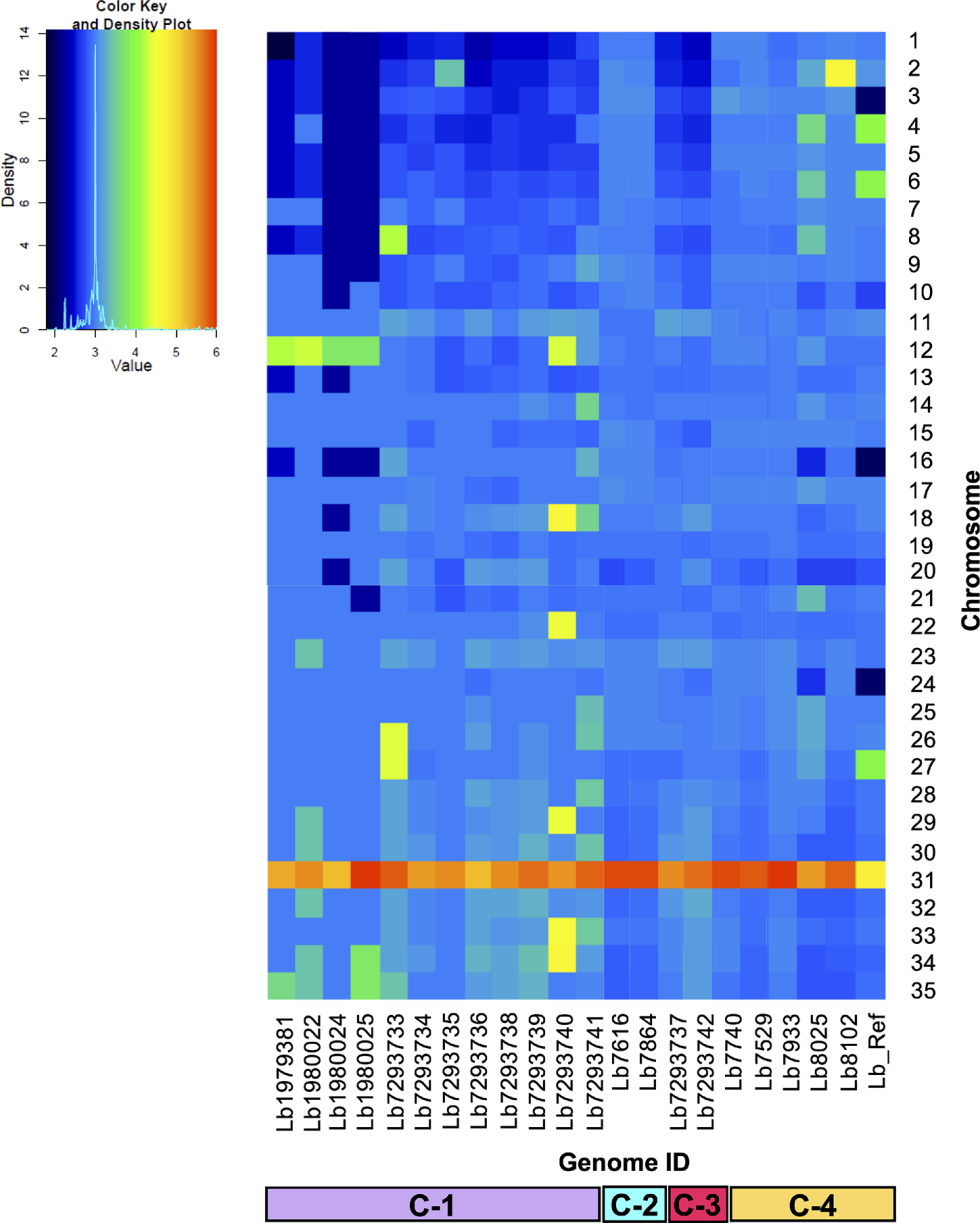
Dynamic of somy between the 21 *Leishmania braziliensis* genomes analyzed. The heatmap shows the copy number of the 35 chromosomes (y-axis) for the 21 genomes analyzed, included the reference genome (Lb_Ref) (x-axis). Trisomic (3, blue), tetrasomic (4, green), pentasomic (5, orange).

We analyzed and compared the chromosome copy numbers in the 21 genomes included in this study. The results revealed that in some genomes the karyotype remained unchanged, most of the chromosomes presented a *S* value between 2.5 and 3.5, which indicate its trisomic state, with the consistent exception of chromosome 31, however, in other genomes we observed a moderate aneuploidy. In clade 1, the Lb7293740 genome presented six chromosomes (12, 18, 22, 29, 33, and 34) with extra copies, while the Lb7293733 genome presented three chromosomes (8, 26, and 27) with extra copies. Additionally, we observed that in one clade 4 genome (Lb8102), there was an extra copy of chromosome 2 compared with the other genomes of the same clade (**Figure 2**). We confirmed the accuracy of somy value obtained by not observing discordance in the allele frequency counts for each predicted heterozygous SNP and the value obtained between the read depths and allele frequencies.

**Figure 3:**
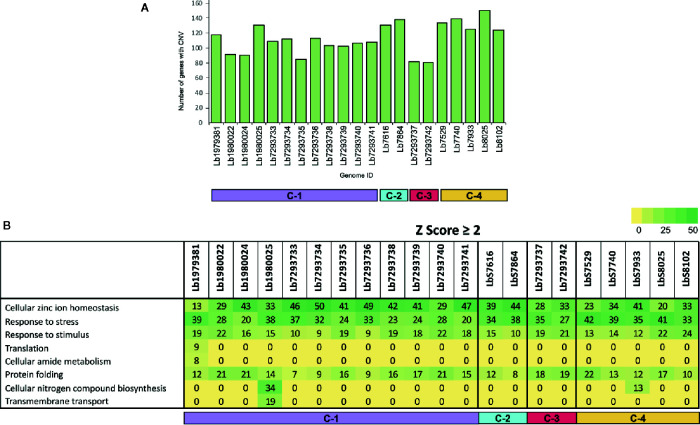
Genes with copy number variation (CNV) and gene ontology enrichment analyses. **(A)** The bar graph represents the number of genes that presented CNVs in the 21 genomes included in the study. **(B)** The table represent the number of genes with CNVs in each genome analyzed, that were involved in different biological processes.

To evaluate the genes with CNVs and compared their occurrence in the genomes analyzed, we observed that the genome with the highest number of genes with CNVs was Lb8025 (147 genes), and the genomes with the lowest numbers were Lb7293737 and Lb7293742 (83 and 80 genes, respectively) (**Figure 3A**). The genes that presented the highest CNVs were found in the Lb8025 genome and encoded: β-tubulin, heat shock protein 83-1 and amastin protein. The genes that exhibit CNVs in the Lb7293737 and Lb7293742 genomes were mainly associated with cell adhesion (e.g. amastin and GP63 leishmanolysin), cell transport (e.g. ABC transporter and glucose transporter 3) and cytoskeletal proteins (e.g. β-tubulin). Additionally, we observed 25 genes with CNVs that were shared between all the genomes analyzed, 22 (88%) of which had known functions and three of which (12%) were annotated as hypothetical proteins. The genes with known function encoded to heat shock protein 83 and 70, α- and β-tubulin, amastin, zinc transporter, and NADH-dependent fumarate reductase (**Supplementary Table S2**). Finally, the Gene Ontology enrichment analysis revealed that the genes with CNVs were mainly associated with cellular zinc ion homeostasis, response to stress, response to stimulus, and protein folding (**Figure 3B**).

### SNPs Estimations

**Figure 4:**
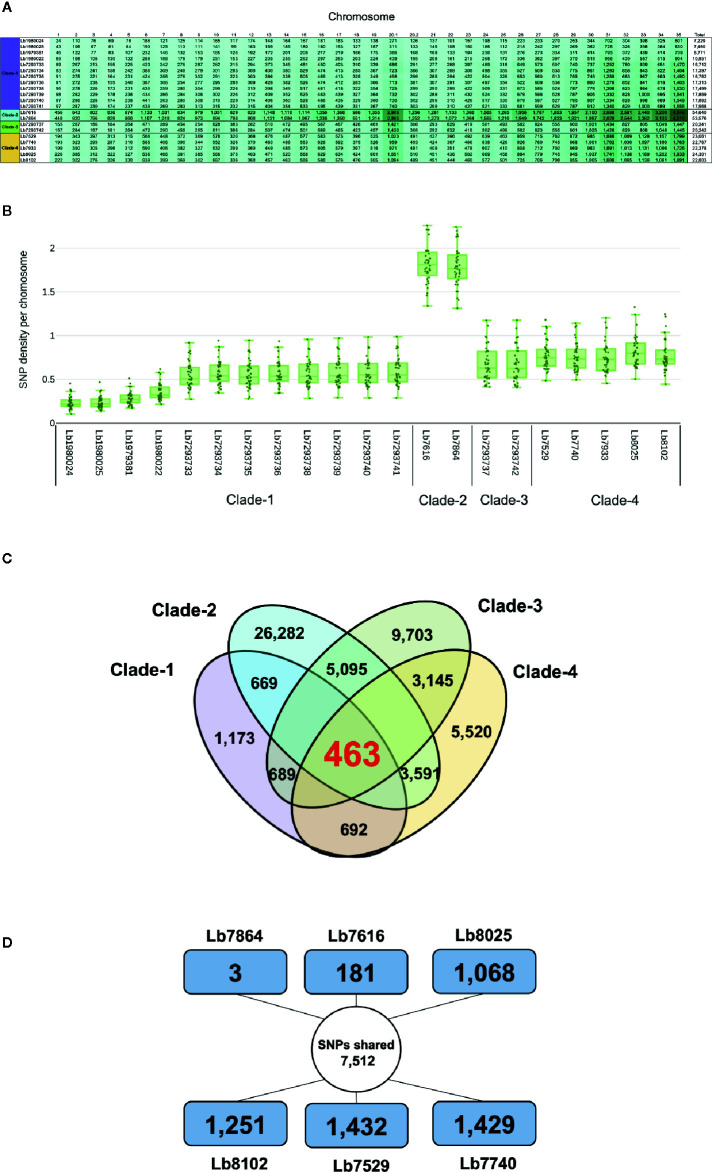
Overview of SNPs identified in *L. braziliensis* genomes. **(A)** Number of SNPs per chromosome, that have a potential effect on gene function (high and moderate impact) in each of the genomes analyzed. The clade is indicated at left. **(B)** SNP density of 21 *L. braziliensis* genomes analyzed. **(C)** Number of SNPs unique and shared between and within the four clades identified. The colors represent each clade: clade 1 (Purple), clade 2 (sky blue), clade 3 (green) and clade 4 (light orange). **(D)** Summary of the number of SNPs unique and shared between the six Colombian genomes analyzed, the external square represents the unique SNPs and the inner circle the shared SNPs.

Comparison of each genome with the reference *L. braziliensis* sequence revealed a significant difference in the total number of SNPs per genome in each clade analyzed. The genomes of clade 1 contain between 42,617 and 68,989 SNPs, the genomes in clade 2 contain ~ 423,200 SNPs and the genomes belonging to clades 3 and 4 contain between 104,316 and 150,639 SNPs (**Supplementary Table S3**). Of the total number of SNPs identified ~20% had a potential effect on gene function (high and moderate impact). The genomes with the highest number of SNPs with functional impact were Lb7616 (54,640) and Lb7864 (53,576), belonging to clade 2, followed by the clade 4 genomes (~23,000 SNPs) (**Figure 4A**). Considering the SNP density between the genomes analyzed, we observed that this density was variable, ranging from less than 1 SNP/Kb in genomes belonging to cluster 1, 3 and 4 to 1.7 SNPs/Kb in genomes belonging to cluster 2. The least polymorphic genomes were Lb1980024 and Lb1980025 belonging to cluster 1 (0.23 and 0.24 SNPs/Kb, respectively), and the genomes with greatest variability were the Lb7616 and Lb7864 genomes belonging to cluster 2 (1.7 SNPs/Kb) (**Figure 4B**). Additionally, we evaluated and compared the nucleotide diversity between Colombian and Brazilian genomes and observed a low diversity in Brazilian genomes (π = 0.072) compared with the Colombian genomes (π = 0.30).

When we analyzed all genomes for SNP variants, we identify ~ 85% were synonymous variants; however, some variants had high functional impact (e.g. stop_gained and stop_lost) (**Table 2**). The SNPs associated to stop gained were identified in both genes encoding hypothetical proteins and in genes encoding to known function proteins such as surface proteins (amastin surface protein and phosphoglycans), multidrug resistance proteins, transporter proteins (pteridine transporter, ABC1 transporter, and iron/zinc transporter protein), and cytoskeletal proteins (β-tubulin), while the SNPs associated to stop lost were identified mainly in genes encoding hypothetical proteins and some of them in genes encoding transmembrane proteins (Meckelin transmembrane protein 67 putative) or transporter proteins (pteridine transporter) (**Supplementary Table S4**).

**Table 2:** Number of SNPs with potential effect on gene function, in each genome analyzed.

Database accession number	Genome ID	Number of Stop_lost	Number of Stop_gained	Synonymous variant
SRR1979381	Lb1979381	8	23	8722
SRR1980022	Lb1980022	9	28	10793
SRR1980024	Lb1980024	6	18	6294
SRR1980025	Lb1980025	4	21	7420
SRR7293733	Lb7293733	15	44	16626
SRR7293734	Lb7293734	11	56	17298
SRR7293735	Lb7293735	15	45	16677
SRR7293737	Lb7293737	11	57	7059
SRR7293738	Lb7293738	13	46	17413
SRR7293739	Lb7293739	15	49	17777
SRR7293740	Lb7293740	15	51	17788
SRR7293741	Lb7293741	15	49	17866
ERS4385934	Lb7616	30	121	54407
ERS4385933	Lb7864	35	118	53348
SRR7293736	Lb7293736	16	57	17213
SRR7293742	Lb7293742	18	56	20139
ERS4385937	Lb7529	19	57	22943
ERS4385938	Lb7740	20	60	22647
ERS4385939	Lb7933	20	55	22264
ERS4385935	Lb8025	18	58	24183
ERS4385936	Lb8102	19	61	22691

The next step was to evaluate the shared SNPs (with high and moderate functional impact) within and between each clade. The comparison of genomes within each clade revealed that the genomes of clade 2 had the highest number of shared SNPs (26,282) compared with clades 1, 3, and 4, where the number of SNPs shared was 1,173 and 9,703, and 5,520, respectively (**Figure 4C**). Fifty-two percent of shared SNPs in each clade were in genes encoding to hypothetical protein, and the remaining 48% were in genes encoding to proteins with known function. The comparison between the four clades allowed to identify 463 SNPs shared with known function (**Figure 4C**). Most of them identified in genes encoding transporter proteins (ABC transporter, nucleoside transporter, zinc transporter, and sugar transporter), host-pathogen interaction-associated proteins (amastin surface protein), kinetoplast-associated protein, or intracellular degradation-associated proteins (ubiquitin-conjugating enzyme putative) (**Supplementary Table S5**). Regarding the shared SNPs between the clades, we observed a high number of shared SNPs between clade 2 and clade 3 (5,095) followed by the clades 2 and 4 (3,591) and clades 3 and 4 (3,145); additionally, we observed that the genomes belonging to clade 1 presented a low number of shared SNPs with the other clades (clades 1 and 2: 669; clades 1 and 3: 689; clades 1 and 4: 692) (**Figure 4C**).

Lastly, we evaluated the unique/shared SNPs (with high and moderate functional impact) between the Colombian genomes included in the study. To analyze the unique SNPs in each genome, we observed that Lb7864 and Lb7616 were the genomes with the lowest number of SNPs (3 and 181, respectively), which represent less than 1% of the total, compared with the other genomes analyzed where the number of unique SNPs ranged between 9 and 13% (**Figure 4D**). Regarding the shared SNPs, the results revealed 7,512 SNPs between the genomes analyzed, 48% of them in genes encoding proteins associated with hypothetical function and the remaining 52% in genes with known function (**Figure 4D**). Later, we made a pairwise comparison considering not only the SNPs with high and moderate functional impact but also those located in genes with known function. The results revealed that the genomes with the highest number of shared SNPs between them were the genomes Lb7864 and Lb7616 (26,763 SNPs), followed by the genomes Lb7529, Lb8025, and Lb7740, which presented a number of shared SNPs ranging from 7,700 to 7,897 (**Supplementary Table S6**).

### LOH and Hybridization in *L. braziliensis*

**Figure 5:**
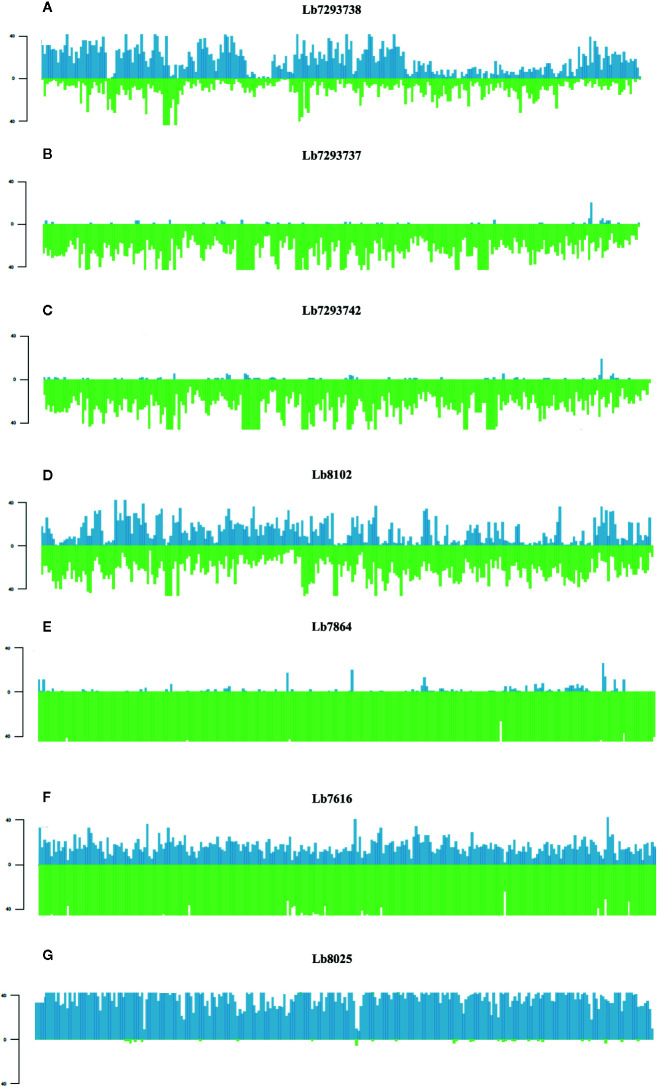
Homozygosity/heterozygosity profile of chromosome 35 in *L. braziliensis* Colombian/Brazilian genomes. The distribution of homozygous (green color) and heterozygous (blue color) SNPs along chromosome 35. The X axis represents 10 kb windows of chromosome 35 and the Y axis indicates the total number of SNPs. Each panel represents a different genome; **(A–C)** represent the Brazilian genomes and **(D–G)** the Colombian genomes.

Finally, of the SNPs with high and moderate functional impact, 73% were homozygous and 27% heterozygous. The most interesting result was the whole genome loss of heterozygosity (LOH) observed in some genomes (**Figure 5**). One Colombian genome (Lb7864) and two Brazilian genomes (Lb7293737 and Lb7293742) presented LOH in all 35 chromosomes (**Supplementary Figure S2** and **Supplementary Table S7**). To explain graphically these results, we selected one of the largest chromosomes comprising the *L. braziliensis* genome (Chromosome 35) and compared the heterozygosity/homozygosity (blue/green, respectively) along this chromosome among seven genomes (**Figure 5**). **Figures 5A–C** represent the Brazilian genomes and clearly show the absence of heterozygous SNPs (blue color) along chromosome 35 in the Lb7293737 and Lb7293742 genomes compared with Lb7293738 genome. Panels D–F represent Colombian genomes and show the high levels of homozygous SNPs (green color) in the Lb7616 and Lb7864 genomes (**Figure 5**).

**Figure 6:**
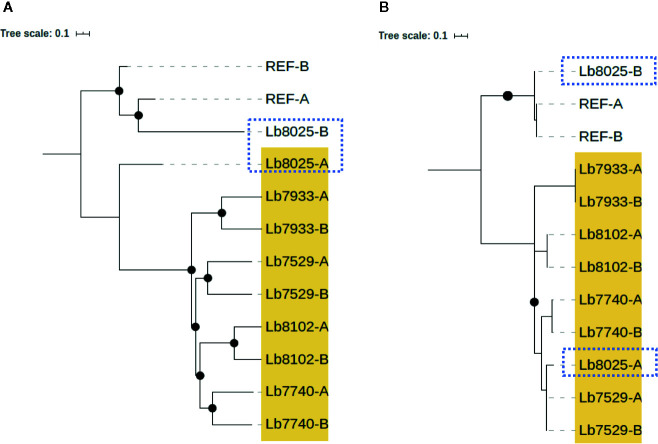
Phylogenetic reconstruction based on genomic SNP alignments for phased haplotypes belonging to Clade 4. The trees represent phylogenetic analysis from whole nuclear **(A)** and mitochondrial (maxicircle)  **(B)** single-nucleotide polymorphism (SNP) alignments based on phased haplotypes of five genome sequences belonging clade 4 (highlighted in yellow). MHOM/BR75/M2904_2019 *L. braziliensis* (REF) was included as reference genome. Black dots represent well-supported nodes (Bootstrap ≥ 90) and the dotted squares show the location of Lb8025 haplotypes.

In contrast, we identified one genome of clade 4 (Lb8025) to be highly heterozygous throughout the genome (99% heterozygous SNPs) (**Supplementary Table S7**). This finding, which was consistent in all chromosomes (**Figure 5G** and **Supplementary Figure S2**), together with the divergence observed when compared with the others belonging to the same clade (**Supplementary Figure S3**), suggest a possible event of hybridization. With the purpose to analyze this possible event, we selected the genomes closely related to Lb8025 (Lb7933, Lb8102, Lb7529, and Lb7740) (**Figure 1**) and reconstructed haplotypes of each of them from both nuclear and mitochondrial genomes and by chromosome through phasing the genotype calls. The results obtained when analyzing the topologies of trees from the nuclear (**Figure 6A**) and mitochondrial genomes (**Figure 6B**) and the results obtained by chromosome (**Supplementary Figure S4**) showed that across the genome, the haplotype A and the haplotype B of Lb8025 (Lb8025-A and Lb8025-B) are grouped in distinct clusters, which contrast with the observed in the other genomes analyzed. This is a strong genomic evidence of hybridization with alleles from independent ancestries.

## Discussion

To understand the genetic heterogeneity of *L. braziliensis*, we used DNA-seq technology and bioinformatic analyses to investigate the genetic structure and the phylogenomic relationships among *L. braziliensis* genomes from Colombian/Bolivian clinical isolates and among genomes of Brazilian *L. braziliensis* isolates archived in public databases. The phylogenetic analysis of nuclear and mitochondrial genomes revealed the presence of two different *L. braziliensis* populations in Colombia (clade 2 and clade 4) (**Figures 1B–E**). We considered that the presence of these clades within Colombia can be associated with (i): the geographical localization of each clade; clade 4 genomes are located outside of mountain ecosystems (east Andes), while the clade 2 genome (Lb7864) occurs in Colombian mountain ecosystems (central cordillera) (**Figure 1A**), indicating that the Andes might generate intra-species diversification, as has been proposed for different arthropods, such as bees (Dick et al., 2004), butterflies (De-Silva et al., 2016; Chazot et al., 2018), arachnids (Salgado-Roa et al., 2018), Triatominae (Gomez-Palacio and Triana, 2014; Monsalve et al., 2016), *Lutzomyia* species (Gonzalez et al., 2014; Ferro et al., 2015) and plants, such as *Phlegmariurus* (Testo et al., 2019). (ii): Constant human displacement due to violence, armed conflict, or the deployment of military troops from areas of high endemicity (Ore et al., 2015). This could promote the distribution of this parasite to other geographical regions, as has been observed for *L. guyanensis*, which broke out of its native geographical distribution and is now detected in two different habitats (Ferro et al., 2011; Ramirez et al., 2016). Finally, (iii): migration of animal reservoirs or sand fly vectors responsible for transmission of the parasite within the national territory. *L. braziliensis* is associated with a wide variety of sand fly vectors (Bejarano et al., 2002; Testa et al., 2002; Ovallos et al., 2013; Ovalle-Bracho et al., 2019).

Another interesting finding was associated with the clear swapping event (incongruence in the tree topologies) identified in clade 4, when tree topology of nuclear genome was compared with the mitochondrial genome (**Figure 1** and **Supplementary Figure S1**). This finding was mainly observed in the Lb8025 genome, which may represent evidence of introgression, probably by interspecific hybridization, phenomena that has been described as a possible way for rapid evolution, which is key in species responses to environmental change or as a strategy of parasites to escape from Muller’s ratchet (irreversible accumulation of deleterious mutations) (Harrison and Larson, 2014; Messenger and Miles, 2015). This was expected because of recent evidence of hybridization and meiosis-like behavior in *Leishmania* and other trypanosomatids, such as *Trypanosoma cruzi* (Inbar et al., 2019; Schwabl et al., 2019; Broeck et al., 2020). We were able to identify a potential hybrid; however, considered that additional studies are necessary to understand not only the population structure but also to describe the evolutionary forces that possibly have promoted it. Therefore, studies that involved a larger population size from different countries, as well as crosses in sandflies should be performed.

When we then evaluated the somy value; observed that the Brazilian genomes had high karyotype instability compared with the Colombian/Bolivian genomes, of which only two of the seven genomes showed somy change (Lb8102 and Lb8025 genomes) (**Figure 2**). Aneuploidy is considered one of the most important processes for *Leishmania* adaptation; therefore, these results indicate that Colombian strains could be changing to adapt to the eco-epidemiological conditions of the country as the Brazilian strains and that the amplification of some chromosomes may provide fitness advantages during host adaptation, which is consistent with the observed in other *Leishmania* species (*L. donovani*, *L. major*, *L. tropica*, *L. infantum*, and *L. amazonensis*) (Dumetz et al., 2017; Iantorno et al., 2017; Bussotti et al., 2018; Patino et al., 2019b). Additionally, the somy dynamic observed reveals changes in an individualized manner, suggesting that environment-independent intrinsic genetic factors can affect *Leishmania* karyotypic adaptation, as has been described in Old-Word *Leishmania* species (Bussotti et al., 2018). Despite moderate karyotype heterogeneity observed in the Colombian/Bolivian genomes, we highlight an interesting somy dynamic observed in the Lb8025 genome (**Figure 2**), in which the genetic content was doubled in various chromosomes. This, together with the possible introgression event (**Figure 1** and **Supplementary Figure S1**), the elevated heterozygosity (**Figure 5G**, **Supplementary Figure S2**, and **Supplementary Table S7**), the divergence compared with other genomes of the same clade (**Supplementary Figure S3**), and the grouping of each haplotype in two different clusters (**Figure 6** and **Supplementary Figure S4**) indicate that this strain is a hybrid (via intra or interspecific hybridization or genomic recombination). The genetic exchange product of hybridization event could generate evolutionary advantages that can impact vector permissiveness, the adaptation to different environments/niches, to increase or alter the virulence and generate recombinant progeny that are capable of widespread clonal propagation. Likewise, the pathological implications of hybrid genotypes in human infections, could promote the spread of virulent strains to affect the transmissibility and the resistance to chemotherapeutics (Cortes et al., 2012; Ramirez et al., 2012; Messenger and Miles, 2015).

Another evaluated parameter was local copy number variation (CNV). The results did not reveal important intraspecies variations in the numbers of genes with CNV (**Figure 3A**) either in genomes collected within or outside of Colombia. Of the genes showing CNV in all analyzed genomes (25 in total), we highlight those involved in stress resistance, infectivity and virulence (Heat shock protein 70 and 83, α and β tubulin, amastin, and zinc transporter) (**Supplementary Table S2**); curiously, these genes were previously reported in other studies (Rastrojo et al., 2013; Bifeld and Clos, 2015; Bussotti et al., 2018; Urrea et al., 2018; Restrepo et al., 2019). These results together with the ontology enrichment analysis (**Figure 3B**), indicate that these genes may drive or be the result of rapid adaptation in *L. braziliensis*, results previously described in other *Leishmania* species (Rogers et al., 2011; Bussotti et al., 2018; Urrea et al., 2018) and suggest that genome plasticity in *L. braziliensis* not only at the level of whole chromosome but also of specific regions promotes important processes associated with the replication, infectivity, and virulence.

Likewise, the local copy number variation may represent an evolutionarily important adaptation, to confer a degree of plasticity in the regulation and functional expression of genes or gene clusters involved in drug resistance, adaptation to different environmental, including survival in a variety of mammalian hosts and phenotypic diversity as has been observed in other species (*L. donovani, L. tropica*, *L. infantum*, and *L. panamensis*) (Laffitte et al., 2016; Iantorno et al., 2017; Sinha et al., 2018; Patino et al., 2020).

Our final strategy to evaluate genomic variability among *L. braziliensis* genomes involved analysis of nucleotide-level variations (SNPs). Comparing the number of SNPs found in this study (42,617 to 435,529; n = 21) (**Supplementary Table S3**) and the recent report in *L. braziliensis* isolates from Peru (Broeck et al., 2020), with the number of variants described in other Old/New World *Leishmania* species, such as *L. donovani* (3,549 SNPs; n = 17) (Downing et al., 2011); *L. infantum* (17,333 SNPs; n = 12) (Rogers et al., 2014) (~3,000 SNPs; n = 20) (Teixeira et al., 2017), *L. amazonensis/L. mexicana* vs. *L. infantum* (~21,000 SNPs; n = 2) (Valdivia et al., 2017), *L. panamensis;* (~62,000 SNPs; n = 22) (Patino et al., 2020), *L. amazonensis* vs. reference genome of *L. mexicana* (~40,000 SNPs) (Patino et al., 2019b) and *L. peruviana* vs. reference genome of *L. braziliensis* (~112,000 SNPs; n = 2) (Valdivia et al., 2015), we can confirm that *L. braziliensis*, is the *Leishmania (Viannia)* species with the highest genetic variability circulating in some regions of South America (Brazil, Colombia and Perú). Additionally, to analyze the genetic variability of *L. braziliensis* between the Colombian and Brazilian isolates, we observed that Colombian genomes were much more diverse (π = 0.30; n = 7), than the Brazilian genomes (π = 0.072; n = 14) (**Supplementary Table S3**). This diversity is advantageous for the parasite because it favors survival in diverse ecological systems/niches, determines the distribution of the observed clinical forms of the disease, and influences the anti-leishmanial therapy response; aspects that enable this species to be a successful human pathogen. Despite our results, we consider that additional studies, with a greater number of Colombian samples, are necessary to understand the reasons why *L. braziliensis* is such a highly diverse species.

To evaluate unique/shared SNPs among the four identified clades, we identified a high number of unique SNPs (**Figure 4C**), mainly in the clades with low heterozygosity (**Figure 5** and **Supplementary Table S7**) (genomes belonging to clades 2 and 3). Consistently, we observed a low number of shared SNPs among the four clades (463 SNPs) (**Figure 4C**). The analysis of shared SNPs between clades revealed the low similarity between the genomes belonging to clade 1 and the genomes belonging to other clades, contrary to observed with the genomes of clades 2, 3, and 4, which presented a high similarity between them (high number of shared SNPs), those results confirm the close relationship between the Colombian genomes with some Brazilian genomes (**Figure 4C**). Finally, we analyzed the unique/shared SNPs, but in this case between the six Colombian genomes. The results revealed in some genomes a low number of unique SNPs and a high number of shared SNPs (**Figure 4D**; **Supplementary Table S6**); two of the genomes (Lb7864 and Lb7616) had 99.5% of identity, and this close inter-strain relationship could indicate a recent shared ancestor between them.

In addition to the findings associated with the elevated number of SNPs in the genomes herein analyzed, we highlight the drastic whole genome loss of heterozygosity (LOH) observed in some Brazilian/Colombian genomes (specifically in those belonging to clades 2 and 3) (**Figure 5** and **Supplementary Table S7**). This large-scale of LOH, has been observed in fungi (*Candida albicans* and *Saccharomyces cerevisiae)* as a mechanism for introducing diversity into a population and as a strategy to survive in stressful conditions (response to treatment, DNA damage and host passage) (Dunkel and Morschhauser, 2011; Hirakawa et al., 2015; Wertheimer et al., 2016; Hoffert and Strome, 2019). In *Leishmania*, studies published to date, reveal LOH to short scale (loss of some heterozygous SNPs in a block) (Samarasinghe et al., 2018) or low-level heterozygosity (Downing et al., 2011; Rogers et al., 2014) mainly in Old World *Leishmania* parasites (*L. infantum*, *L. major* and *L. donovani*). Therefore, this is the first study using Colombian clinical isolates and next generation sequencing to identify patterns of homozygosity in the whole genome of one of the most important New World *Leishmania* species, *L. braziliensis*. This pattern of diversity could be explained by a substantial bottleneck of adaptation that occurred in an environment/niche-dependent manner, which favored and fixed certain genotypes in the population and promoted the stochastic loss of others, as has been described for *T. brucei* (Oberle et al., 2010) and recently for *T. terrestris* (Perez et al., 2019). Another possible hypothesis for these findings is associated with gene conversion, which may have produced unidirectional transfer of genetic material between members of the same species sharing the same ecological niche. This would contribute to the genetic diversity and possible evolutionary success of the species, as has been suggested for this and other *Leishmania* species (*L. donovani* complex) (Mauricio et al., 2007; Rougeron et al., 2009), as well as other eukaryotic organisms (*Theileria parva* and *Saccharomyces cerevisiae)* (Qi et al., 2009; Henson et al., 2012; Sriswasdi et al., 2016). The structure of the *L. braziliensis* genome is complex, and additional studies are needed to unveil the existence of homozygous and heterozygous strains and the exact mechanism by which *Leishmania* can fix its heterozygosity including the biological consequences of this plasticity.

In conclusion, the findings in this study demonstrate the high intra-species genetic diversity of *L. braziliensis* in Colombia and the occurrence of distinct phylogenetic groups in the sampled regions. The genomic changes, until date unknown in *L. braziliensis*, such as moderate changes in somy, CNVs, the introgression event, the increase of the heterozygosity and the duplication of genetic content in various chromosomes, identified in the Lb8025 genome (Potential hybrid), as well as the high number of homozygous SNPs (most of them unique for each clade) and the whole genome LOH, identified in Colombian/Brazilian genomes, suggest a striking genomic plasticity of this species, which could be genomic strategies used by *L. braziliensis* to favor its survival and adaptation to different ecological niches. These genomic findings can influence *L. braziliensis* epidemiology and future clinical and therapeutic outcomes.

Although our study was focused to analyze and compare the genomic structure of Colombian/Bolivian isolates with Brazilian isolates, we consider that there are necessary additional studies that include genomes from other regions of South America where *L. braziliensis* is endemic. This at the end will permit not only to make a broader comparative genomic analysis of our Colombian isolates with genomes from other regions but also to expand the knowledge about of genetic variability of this species in South America.

## Data Availability Statement

The datasets presented in this study can be found in online repositories. The names of the repository/repositories and accession number(s) can be found at: https://www.ebi.ac.uk/ena, ERS4385933, ERS4385934, ERS4385935, ERS4385936, ERS4385937, ERS4385938 and ERS4385939.

## Ethics Statement

The studies involving human participants were reviewed and approved by: This study was approved by the Ethics Committee of the Universidad de Antioquia (number VRI3445/2010) in accordance with resolution number 36836. Written informed consent was obtained from the patients from which the strains were isolated. The patients/participants provided their written informed consent to participate in this study.

## Author Contributions

LP conceived and designed the study, analyzed and interpreted the data, and prepared the manuscript. MM interpreted the data and critically revised the manuscript and made important suggestions. LC-S critically revised the manuscript and made important suggestions. CM critically revised the manuscript and made important suggestions. JR conceived and designed the study and revised the manuscript. All authors contributed to the article and approved the submitted version.

## Funding

This work was funded by DIRECCIÓN DE INVESTIGACIÓN E INNOVACIÓN from Universidad del Rosario. JR is a Latin American fellow in Biomedical Sciences, supported by The Pew Charitable Trusts.

## Conflict of Interest

The authors declare that the research was conducted in the absence of any commercial or financial relationships that could be construed as a potential conflict of interest.

## Supplementary Material

Supplementary Figure 1 Tree comparisson of phylogenetic reconstruction from nuclear and mitochondrial alignments for *Leishmania braziliensis* genomes. Phylogenetic tree built based on a distance matrix, derived from nuclear SNPs **(A)** and mitochondrial (maxicircle) SNPs **(B)**, shared among 21 clinical isolates of *L. braziliensis.* Tree topologies were compared to identify swapping events, characterized by changes in clustering patterns. Colors indicate similarity to most common node. A score of 1 denotes the subtree structure of the node is identical to the subtree structure of its best corresponding node. This comparison was performed using Phylo.io interactive tool (http://phylo.io/). Lguy_SRR8179913 (*L. guyanensis*) and LpW (*L. panamensis*) were used as outgroup and MHOM/BR75/M2904_2019 *L. braziliensis* (REF) as reference genome.

Supplementary Figure 2 Homozygosity/heterozygosity profile to all 35 chromosomes in *L braziliensis* Colombian/Brazilian genomes. The figure represents the distribution of homozygous (green color) and heterozygous (blue color) SNPs along chromosome. The X axis represents 10 kb windows of chromosome and the Y axis indicates the total number of SNPs. Each panel represents a different genome.

Supplementary Figure 3 Phylogenetic network based on nuclear SNPs alignments for the five genomes belonging Cluster-4. Neighbour-joining network based on genome-wide SNPs for the five genomes belonging clade 4. MHOM/BR75/M2904_2019 *L. braziliensis* (REF) was used as reference genome. The network was constructed using SplitsTree 5.

Supplementary Figure 4 Phylogenetic reconstruction based on genomic SNP variation of phased haplotypes belonging Clade 4 per chromosome. The trees represent phylogenetic analysis, per chromosome, of nuclear single-nucleotide polymorphism (SNP) alignments based on phased haplotypes of five genome sequences belonging clade 4 (highlighted in yellow). MHOM/BR75/M2904_2019 *L. braziliensis* (REF) was included as reference genome. Black dots represent well-supported nodes (Bootstrap ≥ 90) and the dotted squares show the ubication of Lb8025 haplotypes.

## References

[B1] Alves-Ferreira E. V. Toledo J. S. De Oliveira A. H. Ferreira T. R. Ruy P. C. Pinzan C. F. (2015). Differential Gene Expression and Infection Profiles of Cutaneous and Mucosal Leishmania braziliensis Isolates from the Same Patient. PLoS Negl. Trop. Dis. 9, e0004018. 10.1371/journal.pntd.0004018 26366580 PMC4569073

[B2] Banu S. S. Meyer W. Ferreira-Paim K. Wang Q. Kuhls K. Cupolillo E. (2019). A novel multilocus sequence typing scheme identifying genetic diversity amongst Leishmania donovani isolates from a genetically homogeneous population in the Indian subcontinent. Int. J. Parasitol. 49, 555–567. 10.1016/j.ijpara.2019.02.010 31108098

[B3] Bejarano E. E. Uribe S. Rojas W. Dario Velez I. (2002). Phlebotomine sand flies (Diptera: Psychodidae) associated with the appearance of urban Leishmaniasis in the city of Sincelejo, Colombia. Mem. Inst. Oswaldo Cruz. 97, 645–647. 10.1590/S0074-02762002000500010 12219128

[B4] Bifeld E. Clos J. (2015). The genetics of Leishmania virulence. Med. Microbiol. Immunol. 204, 619–634. 10.1007/s00430-015-0422-1 26047933

[B5] Brilhante A. F. Lima L. Zampieri R. A. Nunes V. L. B. Dorval M. E. C. Malavazi P. (2019). Leishmania (Viannia) braziliensis type 2 as probable etiological agent of canine cutaneous leishmaniasis in Brazilian Amazon. PLoS One 14, e0216291. 10.1371/journal.pone.0216291 31039202 PMC6490954

[B6] Broeck F. V. D. Savill N. J. Imamura H. Sanders M. Maes I. Cooper S. (2020). Ecological divergence and hybridization of Neotropical Leishmania parasites. 10.1073/pnas.1920136117 PMC7547230 32958676

[B7] Bruna S. Rezende A. M. de Melo Neto O. P. de Brito M. E. F. Brandao Filho S. P. (2019). Identification of divergent Leishmania (Viannia) braziliensis ecotypes derived from a geographically restricted area through whole genome analysis. PLoS Negl. Trop. Dis. 13, e0007382. 10.1371/journal.pntd.0007382 31170148 PMC6581274

[B8] Bussotti G. Gouzelou E. Cortes Boite M. Kherachi I. Harrat Z. Eddaikra N. (2018). Leishmania Genome Dynamics during Environmental Adaptation Reveal Strain-Specific Differences in Gene Copy Number Variation, Karyotype Instability, and Telomeric Amplification. mBio 9. 10.1128/mBio.01399-18 PMC6222132 30401775

[B9] Chazot N. De-Silva D. L. Willmott K. R. Freitas A. V. L. Lamas G. Mallet J. (2018). Contrasting patterns of Andean diversification among three diverse clades of Neotropical clearwing butterflies. Ecol. Evol. 8, 3965–3982. 10.1002/ece3.3622 29721272 PMC5916281

[B10] Cortes S. Esteves C. Mauricio I. Maia C. Cristovao J. M. Miles M. (2012). In vitro and in vivo behaviour of sympatric Leishmania (V.) braziliensis, L. (V.) peruviana and their hybrids. Parasitology 139, 191–199. 10.1017/S0031182011001909 22054424

[B11] Cupolillo E. Brahim L. R. Toaldo C. B. De Oliveira-Neto M. P. De Brito M. E. Falqueto A. (2003). Genetic polymorphism and molecular epidemiology of Leishmania (Viannia) braziliensis from different hosts and geographic areas in Brazil. J. Clin. Microbiol. 41, 3126–3132. 10.1128/jcm.41.7.3126-3132.2003 12843052 PMC165365

[B12] Cysne-Finkelstein L. Silva-Almeida M. Pereira B. A. S. Dos Santos Charret K. Bertho A. L. Bastos L. S. (2018). Evidence of Subpopulations with Distinct Biological Features Within a Leishmania (Viannia) braziliensis Strain. Protist 169, 107–121. 10.1016/j.protis.2017.11.004 29482071

[B13] De Oliveira G. M. Madeira Mde F. Oliveira F. S. Pires M. Q. Pacheco Rda S. (2013). Canine Cutaneous Leishmaniasis: Dissemination and Tissue Tropism of Genetically Distinct Leishmania (Viannia) braziliensis Populations. Vet. Med. Int. 2013, 982183. 10.1155/2013/982183 23844317 PMC3694552

[B14] De-Silva D. L. Elias M. Willmott K. Mallet J. Day J. J. (2016). Diversification of clearwing butterflies with the rise of the Andes. J. Biogeogr. 43, 44–58. 10.1111/jbi.12611 27546953 PMC4973677

[B15] Dick C. W. Roubik D. W. Gruber K. F. Bermingham E. (2004). Long-distance gene flow and cross-Andean dispersal of lowland rainforest bees (Apidae: Euglossini) revealed by comparative mitochondrial DNA phylogeography. Mol. Ecol. 13, 3775–3785. 10.1111/j.1365-294X.2004.02374.x 15548290

[B16] Downing T. Imamura H. Decuypere S. Clark T. G. Coombs G. H. Cotton J. A. (2011). Whole genome sequencing of multiple Leishmania donovani clinical isolates provides insights into population structure and mechanisms of drug resistance. Genome Res. 21, 2143–2156. 10.1101/gr.123430.111 22038251 PMC3227103

[B17] Dumetz F. Imamura H. Sanders M. Seblova V. Myskova J. Pescher P. (2017). Modulation of Aneuploidy in Leishmania donovani during Adaptation to Different In Vitro and In Vivo Environments and Its Impact on Gene Expression. MBio 8. 10.1128/mBio.00599-17 PMC5442457 28536289

[B18] Dunkel N. Morschhauser J. (2011). Loss of heterozygosity at an unlinked genomic locus is responsible for the phenotype of a Candida albicans sap4Delta sap5Delta sap6Delta mutant. Eukaryot. Cell. 10, 54–62. 10.1128/EC.00281-10 21097666 PMC3019794

[B19] Ferro C. Marin D. Gongora R. Carrasquilla M. C. Trujillo J. E. Rueda N. K. (2011). Phlebotomine vector ecology in the domestic transmission of American cutaneous leishmaniasis in Chaparral, Colombia. Am. J. Trop. Med. Hyg. 85, 847–856. 10.4269/ajtmh.2011.10-0560 22049038 PMC3205630

[B20] Ferro C. Lopez M. Fuya P. Lugo L. Cordovez J. M. Gonzalez C. (2015). Spatial Distribution of Sand Fly Vectors and Eco-Epidemiology of Cutaneous Leishmaniasis Transmission in Colombia. PLoS One 10, e0139391. 10.1371/journal.pone.0139391 26431546 PMC4592259

[B21] Fotouhi-Ardakani R. Dabiri S. Ajdari S. Alimohammadian M. H. Alaeenovin E. Taleshi N. (2016). Assessment of nuclear and mitochondrial genes in precise identification and analysis of genetic polymorphisms for the evaluation of Leishmania parasites. Infect. Genet. Evol. 46, 33–41. 10.1016/j.meegid.2016.10.011 27765638

[B22] Franssen S. U. Durrant C. Stark O. Moser B. Downing T. Imamura H. (2020). Global genome diversity of the Leishmania donovani complex. Elife 9. 10.7554/eLife.51243 PMC7105377 32209228

[B23] Ghouila A. Guerfali F. Z. Atri C. Bali A. Attia H. Sghaier R. M. (2017). Comparative genomics of Tunisian Leishmania major isolates causing human cutaneous leishmaniasis with contrasting clinical severity. Infect. Genet. Evol. 50, 110–120. 10.1016/j.meegid.2016.10.029 27818279 PMC5376240

[B24] Gomez-Palacio A. Triana O. (2014). Molecular evidence of demographic expansion of the chagas disease vector Triatoma dimidiata (Hemiptera, Reduviidae, Triatominae) in Colombia. PLoS Negl. Trop. Dis. 8, e2734. 10.1371/journal.pntd.0002734 24625572 PMC3953067

[B25] Gonzalez C. Paz A. Ferro C. (2014). Predicted altitudinal shifts and reduced spatial distribution of Leishmania infantum vector species under climate change scenarios in Colombia. Acta Trop. 129, 83–90. 10.1016/j.actatropica.2013.08.014 23988300

[B26] Harrison R. G. Larson E. L. (2014). Hybridization, introgression, and the nature of species boundaries. J. Hered. 105 (Suppl 1), 795–809. 10.1093/jhered/esu033 25149255

[B27] Henson S. Bishop R. P. Morzaria S. Spooner P. R. Pelle R. Poveda L. (2012). High-resolution genotyping and mapping of recombination and gene conversion in the protozoan Theileria parva using whole genome sequencing. BMC Genomics 13, 503. 10.1186/1471-2164-13-503 22998600 PMC3575351

[B28] Herrera G. Hernandez C. Ayala M. S. Florez C. Teheran A. A. Ramirez J. D. (2017). Evaluation of a Multilocus Sequence Typing (MLST) scheme for Leishmania (Viannia) braziliensis and Leishmania (Viannia) panamensis in Colombia. Parasit. Vectors 10, 236. 10.1186/s13071-017-2175-8 28499458 PMC5429539

[B29] Hirakawa M. P. Martinez D. A. Sakthikumar S. Anderson M. Z. Berlin A. Gujja S. (2015). Genetic and phenotypic intra-species variation in Candida albicans. Genome Res. 25, 413–425. 10.1101/gr.174623.114 25504520 PMC4352881

[B30] Hoffert K. M. Strome E. D. (2019). Single-Gene Deletions Contributing to Loss of Heterozygosity in Saccharomyces cerevisiae: Genome-Wide Screens and Reproducibility. G3 (Bethesda) 9, 2835–2850. 10.1534/g3.119.400429 31270132 PMC6723133

[B31] Huson D. H. Bryant D. (2006). Application of phylogenetic networks in evolutionary studies. Mol. Biol. Evol. 23, 254–267. 10.1093/molbev/msj030 16221896

[B32] Iantorno S. A. Durrant C. Khan A. Sanders M. J. Beverley S. M. Warren W. C. (2017). Gene Expression in Leishmania Is Regulated Predominantly by Gene Dosage. mBio 8. 10.1128/mBio.01393-17 PMC5596349 28900023

[B33] Imamura H. Downing T. Van Den Broeck F. Sanders M. J. Rijal S. Sundar S. (2016). Evolutionary genomics of epidemic visceral leishmaniasis in the Indian subcontinent. Elife 5. 10.7554/eLife.12613 PMC4811772 27003289

[B34] Inbar E. Shaik J. Iantorno S. A. Romano A. Nzelu C. O. Owens K. (2019). Whole genome sequencing of experimental hybrids supports meiosis-like sexual recombination in Leishmania. PLoS Genet. 15, e1008042. 10.1371/journal.pgen.1008042 31091230 PMC6519804

[B35] Jirmanus L. Glesby M. J. Guimaraes L. H. Lago E. Rosa M. E. Machado P. R. (2012). Epidemiological and clinical changes in American tegumentary leishmaniasis in an area of Leishmania (Viannia) braziliensis transmission over a 20-year period. Am. J. Trop. Med. Hyg. 86, 426–433. 10.4269/ajtmh.2012.11-0378 22403312 PMC3284357

[B36] Kuhls K. Cupolillo E. Silva S. O. Schweynoch C. Boite M. C. Mello M. N. (2013). Population structure and evidence for both clonality and recombination among Brazilian strains of the subgenus Leishmania (Viannia). PLoS Negl. Trop. Dis. 7, e2490. 10.1371/journal.pntd.0002490 24205418 PMC3814519

[B37] Laffitte M. N. Leprohon P. Papadopoulou B. Ouellette M. (2016). Plasticity of the Leishmania genome leading to gene copy number variations and drug resistance. F1000Res 5, 2350. 10.12688/f1000research.9218.1 27703673 PMC5031125

[B38] Letunic I. Bork P. (2019). Interactive Tree Of Life (iTOL) v4: recent updates and new developments. Nucleic Acids Res. 47, W256–W259. 10.1093/nar/gkz239 30931475 PMC6602468

[B39] Marco J. D. Barroso P. A. Locatelli F. M. Cajal S. P. Hoyos C. L. Nevot M. C. (2015). Multilocus sequence typing approach for a broader range of species of Leishmania genus: describing parasite diversity in Argentina. Infect. Genet. Evol. 30, 308–317. 10.1016/j.meegid.2014.12.031 25558029

[B40] Marlow M. A. Boite M. C. Ferreira G. E. Steindel M. Cupolillo E. (2014). Multilocus sequence analysis for Leishmania braziliensis outbreak investigation. PLoS Negl. Trop. Dis. 8, e2695. 10.1371/journal.pntd.0002695 24551258 PMC3923721

[B41] Mauricio I. L. Gaunt M. W. Stothard J. R. Miles M. A. (2007). Glycoprotein 63 (gp63) genes show gene conversion and reveal the evolution of Old World Leishmania. Int. J. Parasitol. 37, 565–576. 10.1016/j.ijpara.2006.11.020 17280675

[B42] Meireles C. B. Maia L. C. Soares G. C. Teodoro I. P. P. Gadelha M. Da Silva C. G. L. (2017). Atypical presentations of cutaneous leishmaniasis: A systematic review. Acta Trop. 172, 240–254. 10.1016/j.actatropica.2017.05.022 28526427

[B43] Messenger L. A. Miles M. A. (2015). Evidence and importance of genetic exchange among field populations of Trypanosoma cruzi. Acta Trop. 151, 150–155. 10.1016/j.actatropica.2015.05.007 26188331 PMC4644990

[B44] Monsalve Y. Panzera F. Herrera L. Triana-Chavez O. Gomez-Palacio A. (2016). Population differentiation of the Chagas disease vector Triatoma maculata (Erichson 1848) from Colombia and Venezuela. J. Vector Ecol. 41, 72–79. 10.1111/jvec.12196 27232127

[B45] Nolder D. Roncal N. Davies C. R. Llanos-Cuentas A. Miles M. A. (2007). Multiple hybrid genotypes of Leishmania (viannia) in a focus of mucocutaneous Leishmaniasis. Am. J. Trop. Med. Hyg. 76, 573–578. 17360886

[B46] Oberle M. Balmer O. Brun R. Roditi I. (2010). Bottlenecks and the maintenance of minor genotypes during the life cycle of Trypanosoma brucei. PLoS Pathog. 6, e1001023. 10.1371/journal.ppat.1001023 20686656 PMC2912391

[B47] Ore M. Saenz E. Cabrera R. Sanchez J. F. De Los Santos M. B. Lucas C. M. (2015). Outbreak of Cutaneous Leishmaniasis in Peruvian Military Personnel Undertaking Training Activities in the Amazon Basin 2010. Am. J. Trop. Med. Hyg. 93, 340–346. 10.4269/ajtmh.15-0107 26078320 PMC4530758

[B48] Ovalle-Bracho C. Londono-Barbosa D. Salgado-Almario J. Gonzalez C. (2019). Evaluating the spatial distribution of Leishmania parasites in Colombia from clinical samples and human isolates, (1999 to 2016). PLoS One 14, e0214124. 10.1371/journal.pone.0214124 30917177 PMC6436702

[B49] Ovallos F. G. Silva Y. R. Fernandez N. Gutierrez R. Galati E. A. Sandoval C. M. (2013). The sandfly fauna, anthropophily and the seasonal activities of Pintomyia spinicrassa (Diptera: Psychodidae: Phlebotominae) in a focus of cutaneous leishmaniasis in northeastern Colombia. Mem. Inst. Oswaldo Cruz. 108. 10.1590/S0074-02762013000300007 PMC4005568 23778653

[B50] Page A. J. Taylor B. Delaney A. J. Soares J. Seemann T. Keane J. A. (2016). SNP-sites: rapid efficient extraction of SNPs from multi-FASTA alignments. Microb. Genom. 2, e000056. 10.1099/mgen.0.000056 28348851 PMC5320690

[B51] Paradis E. Claude J. Strimmer K. (2004). APE: Analyses of Phylogenetics and Evolution in R language. Bioinformatics 20, 289–290. 10.1093/bioinformatics/btg412 14734327

[B52] Patino L. H. Mendez C. Rodriguez O. Romero Y. Velandia D. Alvarado M. (2017). Spatial distribution, Leishmania species and clinical traits of Cutaneous Leishmaniasis cases in the Colombian army. PLoS Negl. Trop. Dis. 11, e0005876. 10.1371/journal.pntd.0005876 28850603 PMC5593196

[B53] Patino L. H. Imamura H. Cruz-Saavedra L. Pavia P. Muskus C. Mendez C. (2019a). Major changes in chromosomal somy, gene expression and gene dosage driven by Sb(III) in Leishmania braziliensis and Leishmania panamensis. Sci. Rep. 9, 9485. 10.1038/s41598-019-45538-9 31263131 PMC6603004

[B54] Patino L. H. Muskus C. Munoz M. Ramirez J. D. (2019b). Genomic analyses reveal moderate levels of ploidy, high heterozygosity and structural variations in a Colombian isolate of Leishmania (Leishmania) amazonensis. Acta Trop. 203, 105296. 10.1016/j.actatropica.2019.105296 31836281

[B55] Patino L. H. Munoz M. Muskus C. Mendez C. Ramirez J. D. (2020). Intraspecific Genomic Divergence and Minor Structural Variations in Leishmania (Viannia) panamensis. Genes (Basel) 11. 10.3390/genes11030252 PMC7140786 32120946

[B56] Perez S. D. Grummer J. A. Fernandes-Santos R. C. Jose C. T. Medici E. P. Marcili A. (2019). Phylogenetics, patterns of genetic variation and population dynamics of Trypanosoma terrestris support both coevolution and ecological host-fitting as processes driving trypanosome evolution. Parasit. Vectors 12, 473. 10.1186/s13071-019-3726-y 31604471 PMC6790053

[B57] Perez-Franco J. E. Cruz-Barrera M. L. Robayo M. L. Lopez M. C. Daza C. D. Bedoya A. (2016). Clinical and Parasitological Features of Patients with American Cutaneous Leishmaniasis that Did Not Respond to Treatment with Meglumine Antimoniate. PLoS Negl. Trop. Dis. 10, e0004739. 10.1371/journal.pntd.0004739 27243811 PMC4887049

[B58] Price M. N. Dehal P. S. Arkin A. P. (2009). FastTree: computing large minimum evolution trees with profiles instead of a distance matrix. Mol. Biol. Evol. 26, 1641–1650. 10.1093/molbev/msp077 19377059 PMC2693737

[B59] Qi J. Wijeratne A. J. Tomsho L. P. Hu Y. Schuster S. C. Ma H. (2009). Characterization of meiotic crossovers and gene conversion by whole-genome sequencing in Saccharomyces cerevisiae. BMC Genomics 10, 475. 10.1186/1471-2164-10-475 19832984 PMC2770529

[B60] Quaresma P. F. De Brito C. F. A. Rugani J. M. N. Freire J. M. Baptista R. P. Moreno E. C. (2018). Distinct genetic profiles of Leishmania (Viannia) braziliensis associate with clinical variations in cutaneous-leishmaniasis patients from an endemic area in Brazil. Parasitology 145, 1161–1169. 10.1017/S0031182018000276 29526166

[B61] Ramirez J. D. Guhl F. Messenger L. A. Lewis M. D. Montilla M. Cucunuba Z. (2012). Contemporary cryptic sexuality in Trypanosoma cruzi. Mol. Ecol. 21, 4216–4226. 10.1111/j.1365-294X.2012.05699.x 22774844

[B62] Ramirez J. D. Hernandez C. Leon C. M. Ayala M. S. Florez C. Gonzalez C. (2016). Taxonomy, diversity, temporal and geographical distribution of Cutaneous Leishmaniasis in Colombia: A retrospective study. Sci. Rep. 6, 28266. 10.1038/srep28266 27328969 PMC4916406

[B63] Rastrojo A. Carrasco-Ramiro F. Martin D. Crespillo A. Reguera R. M. Aguado B. (2013). The transcriptome of Leishmania major in the axenic promastigote stage: transcript annotation and relative expression levels by RNA-seq. BMC Genomics 14, 223. 10.1186/1471-2164-14-223 23557257 PMC3637525

[B64] Rego F. D. Da Rocha Lima A. Pereira A. A. S. Quaresma P. F. Pascoal-Xavier M. A. Shaw J. J. (2018). Genetic variant strains of Leishmania (Viannia) braziliensis exhibit distinct biological behaviors. Parasitol. Res. 117, 3157–3168. 10.1007/s00436-018-6014-4 30022292

[B65] Restrepo C. M. De La Guardia C. Sousa O. E. Calzada J. E. Fernandez P. L. Lleonart R. (2013). AFLP polymorphisms allow high resolution genetic analysis of American Tegumentary Leishmaniasis agents circulating in Panama and other members of the Leishmania genus. PLoS One 8, e73177. 10.1371/journal.pone.0073177 24039881 PMC3767818

[B66] Restrepo C. M. Llanes A. De La Guardia C. Lleonart R. (2015). Genome-wide discovery and development of polymorphic microsatellites from Leishmania panamensis parasites circulating in central Panama. Parasit. Vectors 8, 527. 10.1186/s13071-015-1153-2 26459121 PMC4603350

[B67] Restrepo C. M. Llanes A. Cedeno E. M. Chang J. H. Alvarez J. Rios M. (2019). Environmental Conditions May Shape the Patterns of Genomic Variations in Leishmania panamensis. Genes (Basel) 10. 10.3390/genes10110838 PMC6896075 31652919

[B68] Rogers M. B. Hilley J. D. Dickens N. J. Wilkes J. Bates P. A. Depledge D. P. (2011). Chromosome and gene copy number variation allow major structural change between species and strains of Leishmania. Genome Res. 21, 2129–2142. 10.1101/gr.122945.111 22038252 PMC3227102

[B69] Rogers M. B. Downing T. Smith B. A. Imamura H. Sanders M. Svobodova M. (2014). Genomic confirmation of hybridisation and recent inbreeding in a vector-isolated Leishmania population. PLoS Genet. 10, e1004092. 10.1371/journal.pgen.1004092 24453988 PMC3894156

[B70] Roque A. L. Jansen A. M. (2014). Wild and synanthropic reservoirs of Leishmania species in the Americas. Int. J. Parasitol. Parasites Wildl. 3, 251–262. 10.1016/j.ijppaw.2014.08.004 25426421 PMC4241529

[B71] Rougeron V. De Meeus T. Hide M. Waleckx E. Bermudez H. Arevalo J. (2009). Extreme inbreeding in Leishmania braziliensis. Proc. Natl. Acad. Sci. U. S. A. 106, 10224–10229. 10.1073/pnas.0904420106 19497885 PMC2700931

[B72] Salgado-Roa F. C. Pardo-Diaz C. Lasso E. Arias C. F. Solferini V. N. Salazar C. (2018). Gene flow and Andean uplift shape the diversification of Gasteracantha cancriformis (Araneae: Araneidae) in Northern South America. Ecol. Evol. 8, 7131–7142. 10.1002/ece3.4237 30073072 PMC6065347

[B73] Samarasinghe S. R. Samaranayake N. Kariyawasam U. L. Siriwardana Y. D. Imamura H. Karunaweera N. D. (2018). Genomic insights into virulence mechanisms of Leishmania donovani: evidence from an atypical strain. BMC Genomics 19, 843. 10.1186/s12864-018-5271-z 30486770 PMC6262978

[B74] Schliep K. Paradis E. De Oliveira Martins L. Potts A. Whit T. W. (2019). Phylogenetic Reconstruction and Analysis. Package "phangorn". Available at: https://academic.oup.com/bioinformatics/article/27/4/592/198887.

[B75] Schwabl P. Imamura H. Van Den Broeck F. Costales J. A. Maiguashca-Sanchez J. Miles M. A. (2019). Meiotic sex in Chagas disease parasite Trypanosoma cruzi. Nat. Commun. 10, 3972. 10.1038/s41467-019-11771-z 31481692 PMC6722143

[B76] Sinha R. Malar C M. Raghwan Das S. Das S. Shadab M. (2018). Genome Plasticity in Cultured Leishmania donovani: Comparison of Early and Late Passages. Front. Microbiol. 9, 1279. 10.3389/fmicb.2018.01279 30018594 PMC6037818

[B77] Sriswasdi S. Takashima M. Manabe R. Ohkuma M. Sugita T. Iwasaki W. (2016). Global deceleration of gene evolution following recent genome hybridizations in fungi. Genome Res. 26, 1081–1090. 10.1101/gr.205948.116 27440871 PMC4971771

[B78] Supek F. Bosnjak M. Skunca N. Smuc T. (2011). REVIGO summarizes and visualizes long lists of gene ontology terms. PloS One 6, e21800. 10.1371/journal.pone.0021800 21789182 PMC3138752

[B79] Teixeira D. G. Monteiro G. R. G. Martins D. R. A. Fernandes M. Z. Macedo-Silva V. Ansaldi M. (2017). Comparative analyses of whole genome sequences of Leishmania infantum isolates from humans and dogs in northeastern Brazil. Int. J. Parasitol. 47, 655–665. 10.1016/j.ijpara.2017.04.004 28606698 PMC5641220

[B80] Testa J. M. Montoya-Lerma J. Cadena H. Oviedo M. Ready P. D. (2002). Molecular identification of vectors of Leishmania in Colombia: mitochondrial introgression in the Lutzomyia townsendi series. Acta Trop. 84, 205–218. 10.1016/s0001-706x(02)00187-0 12443799

[B81] Testo W. L. Sessa E. Barrington D. S. (2019). The rise of the Andes promoted rapid diversification in Neotropical Phlegmariurus (Lycopodiaceae). New Phytol. 222, 604–613. 10.1111/nph.15544 30326543

[B82] Tihon E. Imamura H. Van Den Broeck F. Vermeiren L. Dujardin J. C. Van Den Abbeele J. (2017). Genomic analysis of Isometamidium Chloride resistance in Trypanosoma congolense. Int. J. Parasitol. Drugs Drug Resist. 7, 350–361. 10.1016/j.ijpddr.2017.10.002 29032180 PMC5645165

[B83] Urrea D. A. Duitama J. Imamura H. Alzate J. F. Gil J. Munoz N. (2018). Genomic Analysis of Colombian Leishmania panamensis strains with different level of virulence. Sci. Rep. 8, 17336. 10.1038/s41598-018-35778-6 30478412 PMC6255768

[B84] Valderrama-Ardila C. Alexander N. Ferro C. Cadena H. Marin D. Holford T. R. (2010). Environmental risk factors for the incidence of American cutaneous leishmaniasis in a sub-Andean zone of Colombia (Chaparral, Tolima). Am. J. Trop. Med. Hyg. 82, 243–250. 10.4269/ajtmh.2010.09-0218 20134000 PMC2813165

[B85] Valdivia H. O. Reis-Cunha J. L. Rodrigues-Luiz G. F. Baptista R. P. Baldeviano G. C. Gerbasi R. V. (2015). Comparative genomic analysis of Leishmania (Viannia) peruviana and Leishmania (Viannia) braziliensis. BMC Genomics 16, 715. 10.1186/s12864-015-1928-z 26384787 PMC4575464

[B86] Valdivia H. O. Almeida L. V. Roatt B. M. Reis-Cunha J. L. Pereira A. A. Gontijo C. (2017). Comparative genomics of canine-isolated Leishmania (Leishmania) amazonensis from an endemic focus of visceral leishmaniasis in Governador Valadares, southeastern Brazil. Sci. Rep. 7, 40804. 10.1038/srep40804 28091623 PMC5238499

[B87] Wertheimer N. B. Stone N. Berman J. (2016). Ploidy dynamics and evolvability in fungi. Philos. Trans. R Soc. Lond. B Biol. Sci. 371. 10.1098/rstb.2015.0461 PMC5095540 28080987

[B88] Wickham H. (2019). Simple, Consistent Wrappers for Common String Operations: Package "stringr". Available at: https://joss.theoj.org/papers/10.21105/joss.01686.pdf.

[B89] Yan L. Yang M. Guo H. Yang L. Wu J. Li R. (2013). Single-cell RNA-Seq profiling of human preimplantation embryos and embryonic stem cells. Nat. Struct. Mol. Biol. 20, 1131–1139. 10.1038/nsmb.2660 23934149

[B90] Zhao S. Guo Y. Sheng Q. Shyr Y. (2014). Advanced heat map and clustering analysis using heatmap3. BioMed. Res. Int. 2014, 986048. 10.1155/2014/986048 25143956 PMC4124803

