# Supplementary figures and images for: Genomic Diversification, Structural Plasticity, and Hybridization in Leishmania (Viannia) braziliensis
Source: Front Cell Infect Microbiol. 2020 Oct 16;10:582192. doi: 10.3389/fcimb.2020.582192 (PMC7596589; doi:10.3389/fcimb.2020.582192)

**A**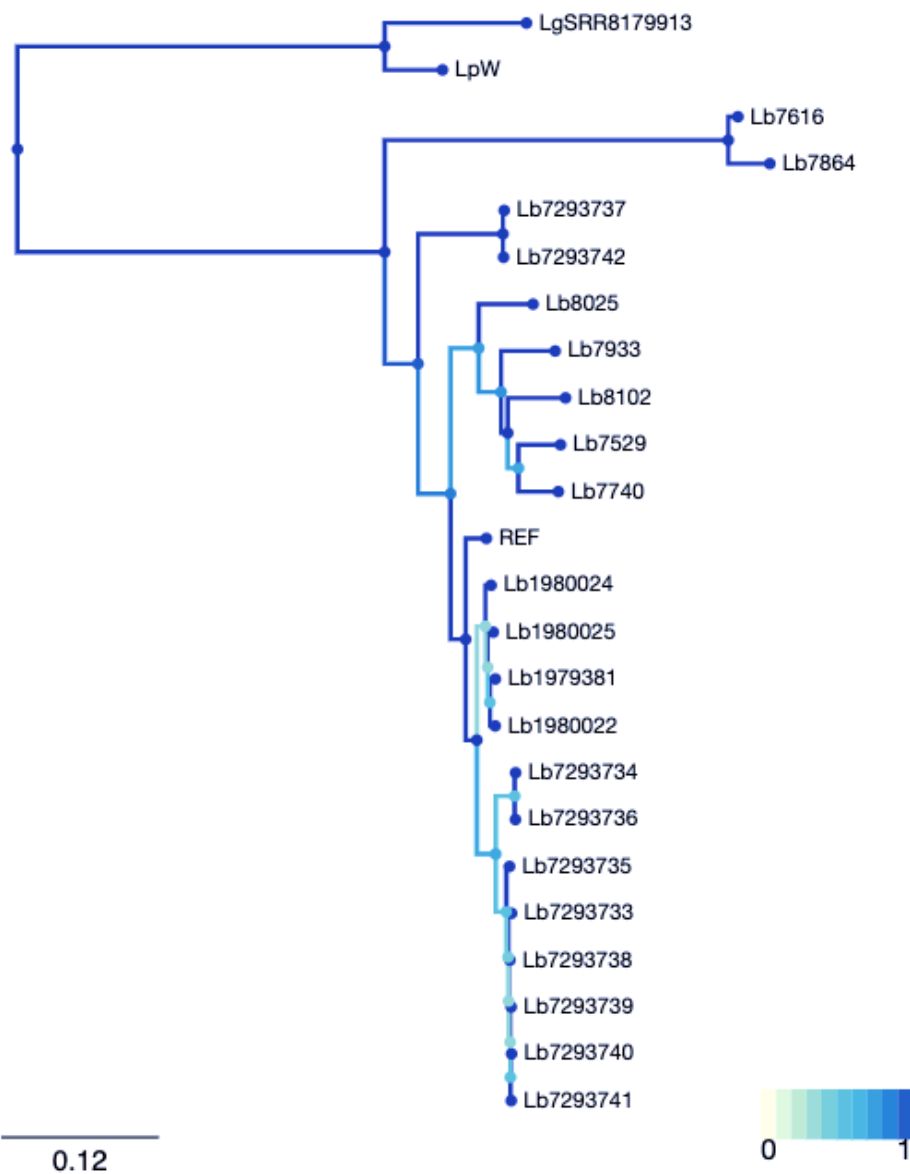**B**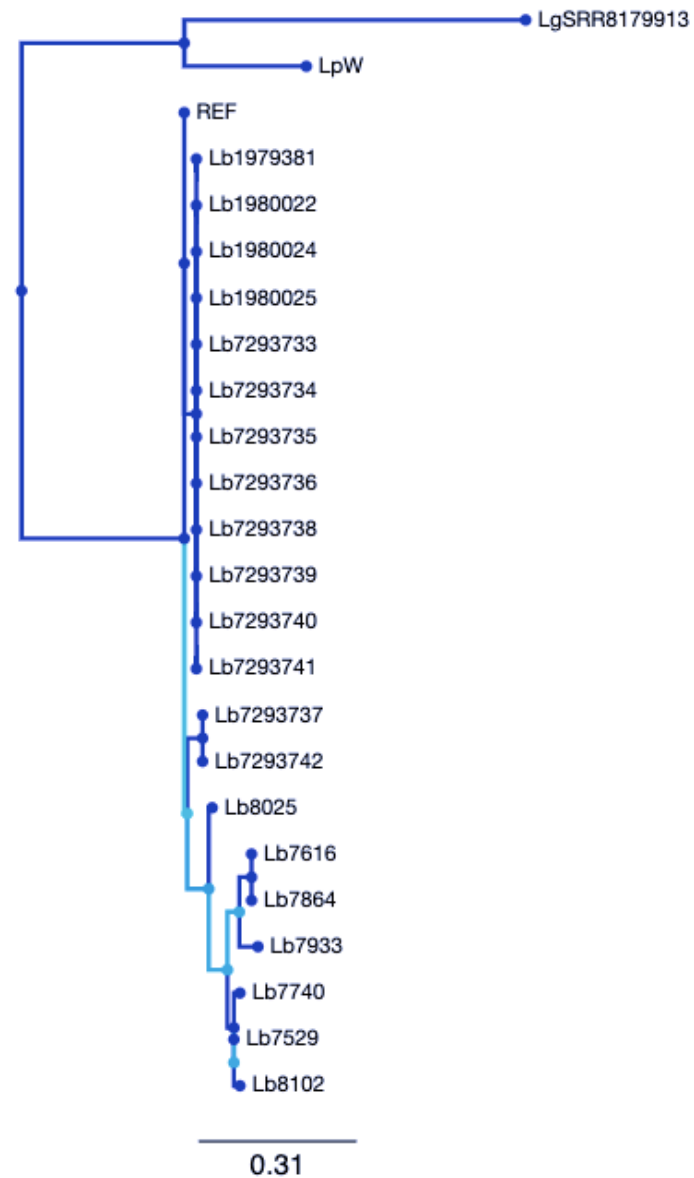

Supplement: Supplementary Figure 1 — Tree comparisson of phylogenetic reconstruction from nuclear and mitochondrial alignments for Leishmania braziliensis genomes. Phylogenetic tree built based on a distance matrix, derived from nuclear SNPs (A) and mitochondrial (maxicircle) SNPs (B), shared among 21 clinical isolates of L. braziliensis. Tree topologies were compared to identify swapping events, characterized by changes in clustering patterns. Colors indicate similarity to most common node. A score of 1 denotes the subtree structure of the node is identical to the subtree structure of its best corresponding node. This comparison was performed using Phylo.io interactive tool (http://phylo.io/). Lguy_SRR8179913 (L. guyanensis) and LpW (L. panamensis) were used as outgroup and MHOM/BR75/M2904_2019 L. braziliensis (REF) as reference genome. [file DataSheet_1.pdf]

0.01

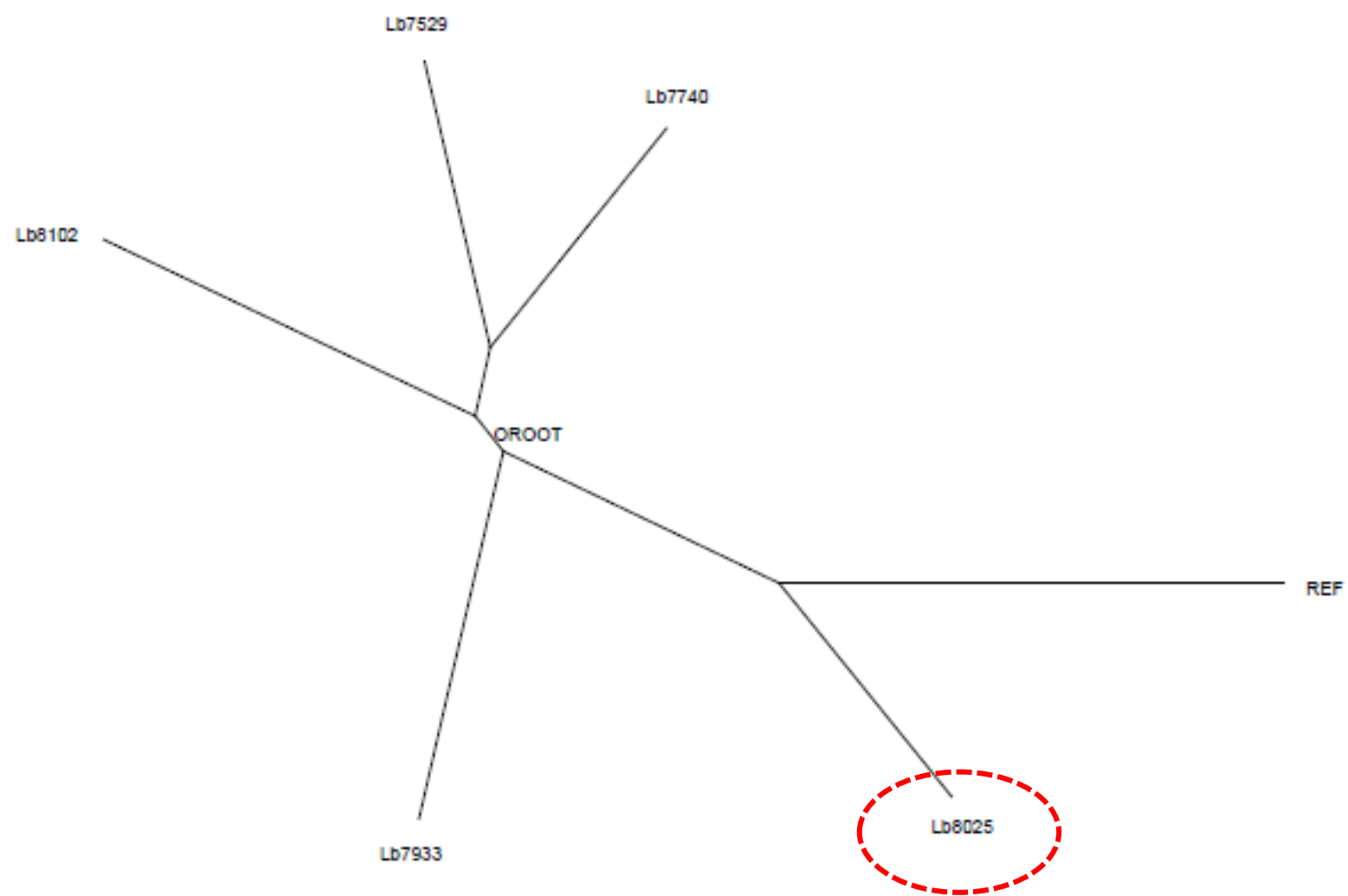

Supplement: Supplementary Figure 3 — Phylogenetic network based on nuclear SNPs alignments for the five genomes belonging Cluster-4. Neighbour-joining network based on genome-wide SNPs for the five genomes belonging clade 4. MHOM/BR75/M2904_2019 L. braziliensis (REF) was used as reference genome. The network was constructed using SplitsTree 5. [file DataSheet_3.pdf]

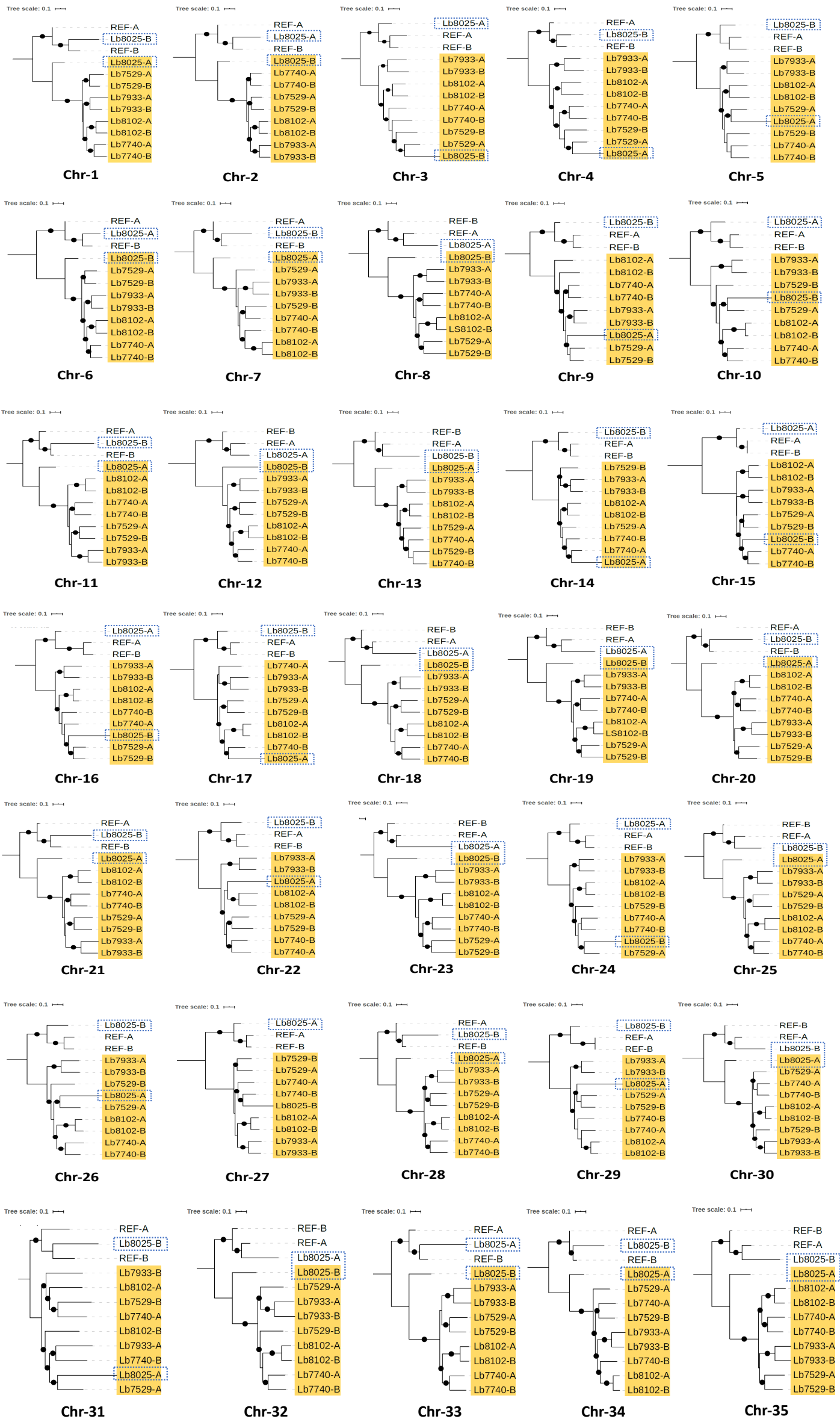

Supplement: Supplementary Figure 4 — Phylogenetic reconstruction based on genomic SNP variation of phased haplotypes belonging Clade 4 per chromosome. The trees represent phylogenetic analysis, per chromosome, of nuclear single-nucleotide polymorphism (SNP) alignments based on phased haplotypes of five genome sequences belonging clade 4 (highlighted in yellow). MHOM/BR75/M2904_2019 L. braziliensis (REF) was included as reference genome. Black dots represent well-supported nodes (Bootstrap ≥ 90) and the dotted squares show the ubication of Lb8025 haplotypes. [file DataSheet_4.pdf]
