# Supplementary material for: Genomic Diversification, Structural Plasticity, and Hybridization in Leishmania (Viannia) braziliensis
Source: Front Cell Infect Microbiol. 2020 Oct 16;10:582192. doi: 10.3389/fcimb.2020.582192 (PMC7596589; doi:10.3389/fcimb.2020.582192)
Supplement: Supplementary Figure 2 — Homozygosity/heterozygosity profile to all 35 chromosomes in L braziliensis Colombian/Brazilian genomes. The figure represents the distribution of homozygous (green color) and heterozygous (blue color) SNPs along chromosome. The X axis represents 10 kb windows of chromosome and the Y axis indicates the total number of SNPs. Each panel represents a different genome. [file DataSheet_2.pdf]

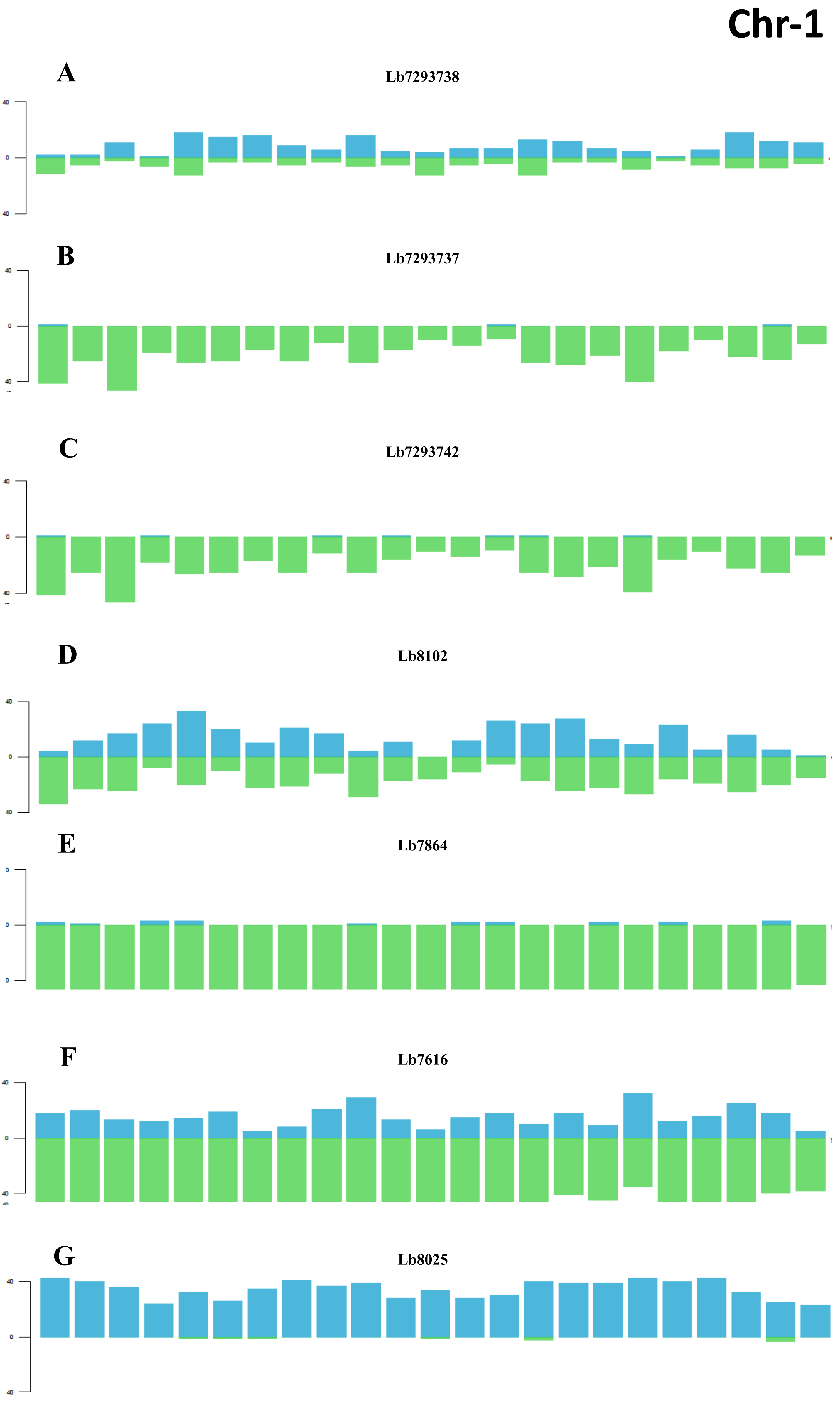
Chr-1
A
Lb7293738
B
Lb7293737
C
Lb7293742
D
Lb8102
E
Lb7864
F
Lb7616
G
Lb8025

Chr-2

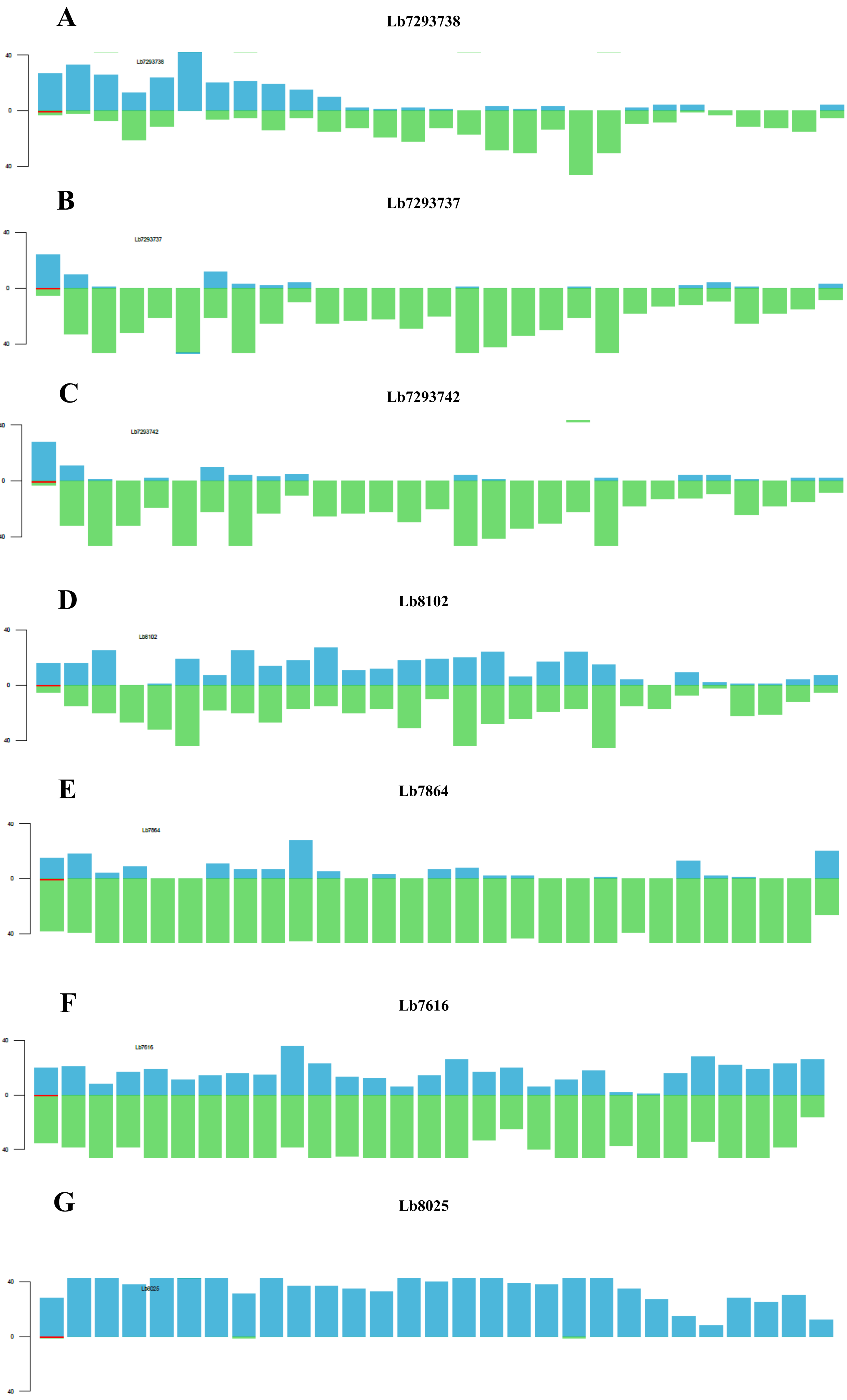
A
Lb7293738
B
Lb7293737
C
Lb7293742
D
Lb8102
E
Lb7864
F
Lb7616
G
Lb8025

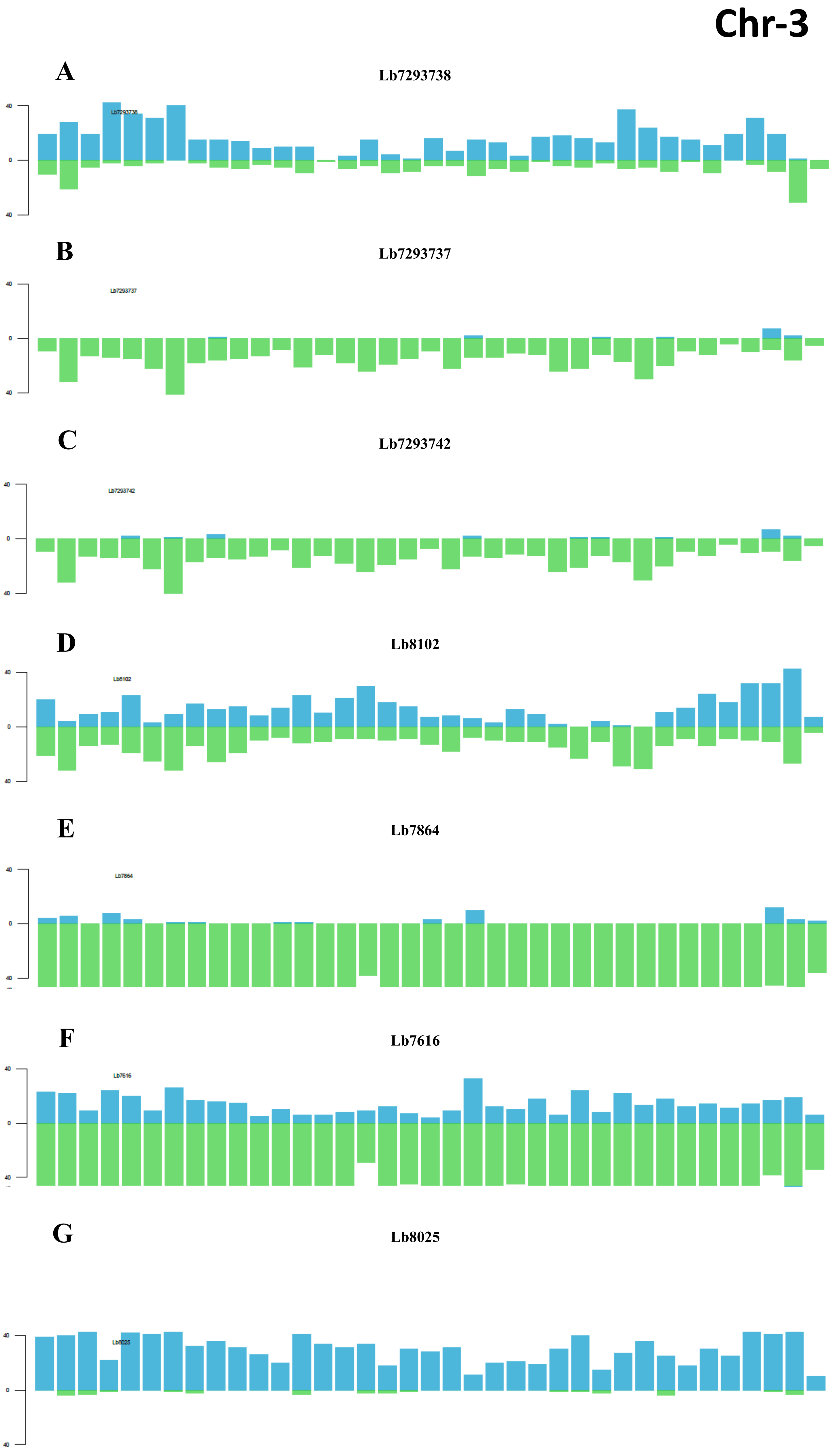
Chr-3
A
Lb7293738
40
0
40
Lb7293738
B
Lb7293737
40
0
40
Lb7293737
C
Lb7293742
40
0
40
Lb7293742
D
Lb8102
40
0
40
Lb8102
E
Lb7864
40
0
40
Lb7864
F
Lb7616
40
0
40
Lb7616
G
Lb8025
40
0
40
Lb8025

Chr-4

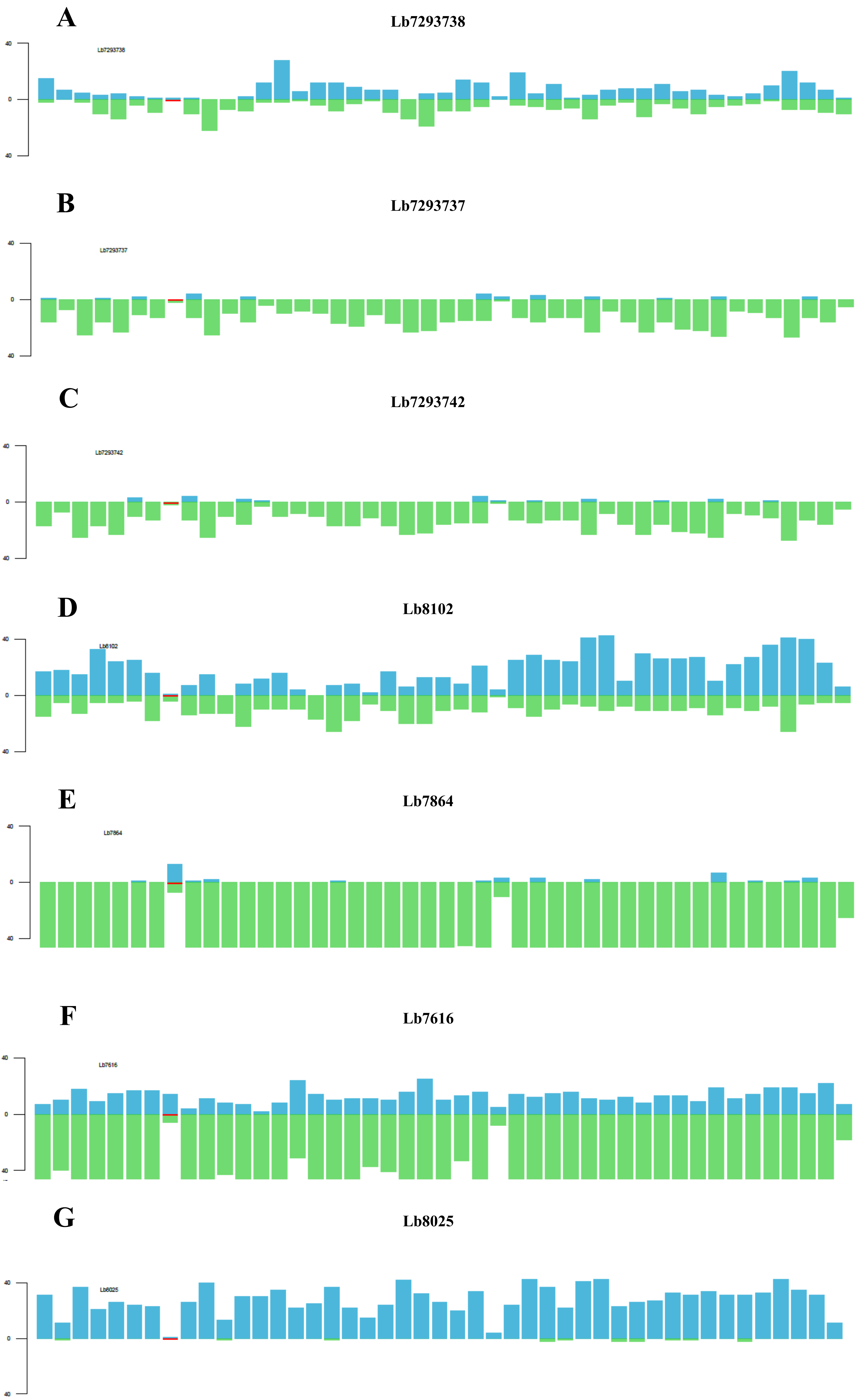

A
Lb7293738
40
0
40
Lb7293738
B
Lb7293737
40
0
40
Lb7293737
C
Lb7293742
40
0
40
Lb7293742
D
Lb8102
40
0
40
Lb8102
E
Lb7864
40
0
40
Lb7864
F
Lb7616
40
0
40
Lb7616
G
Lb8025
40
0
40
Lb8025

Chr-5

A **Lb7293738**

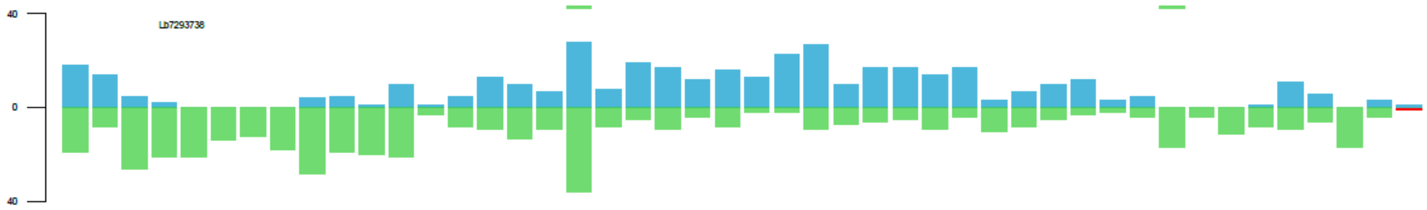

B **Lb7293737**

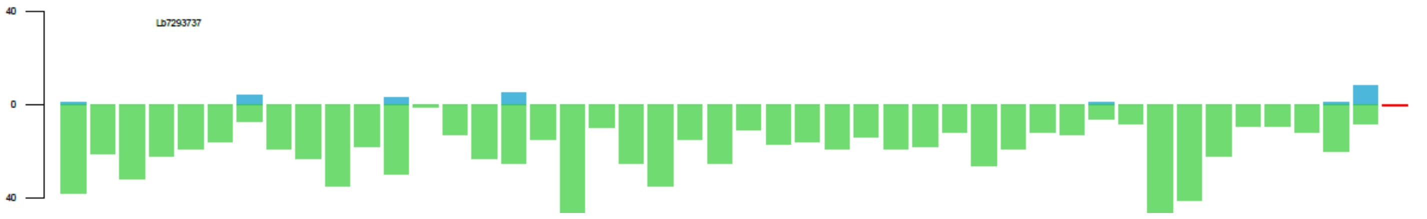

C **Lb7293742**

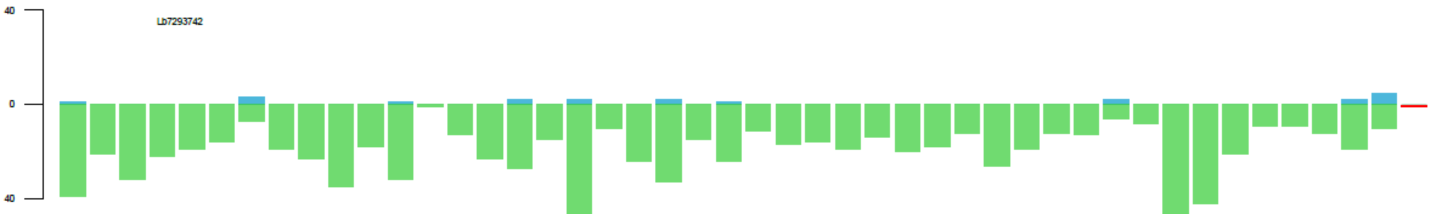

D **Lb8102**

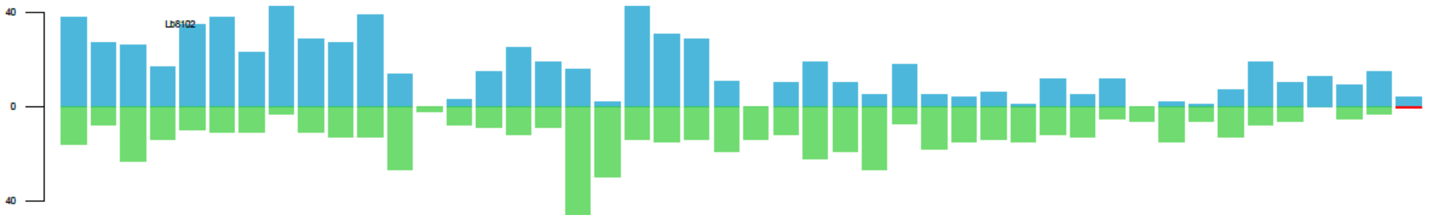

E **Lb7864**

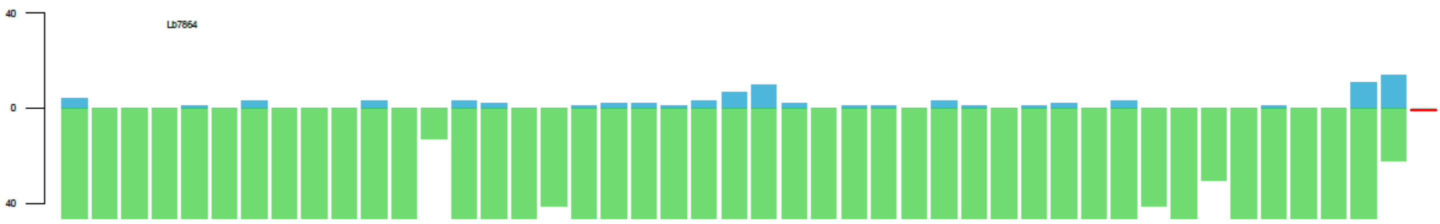

F **Lb7616**

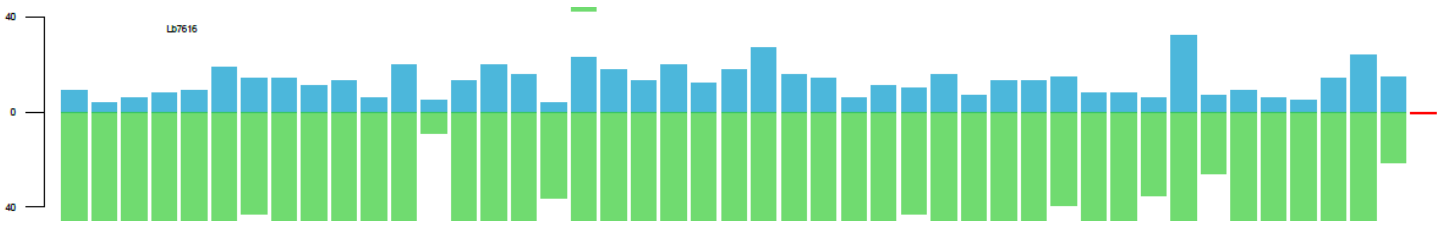

G **Lb8025**

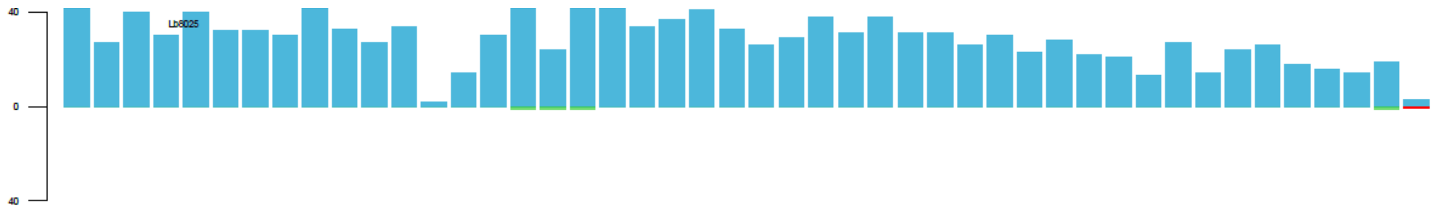

Chr-6

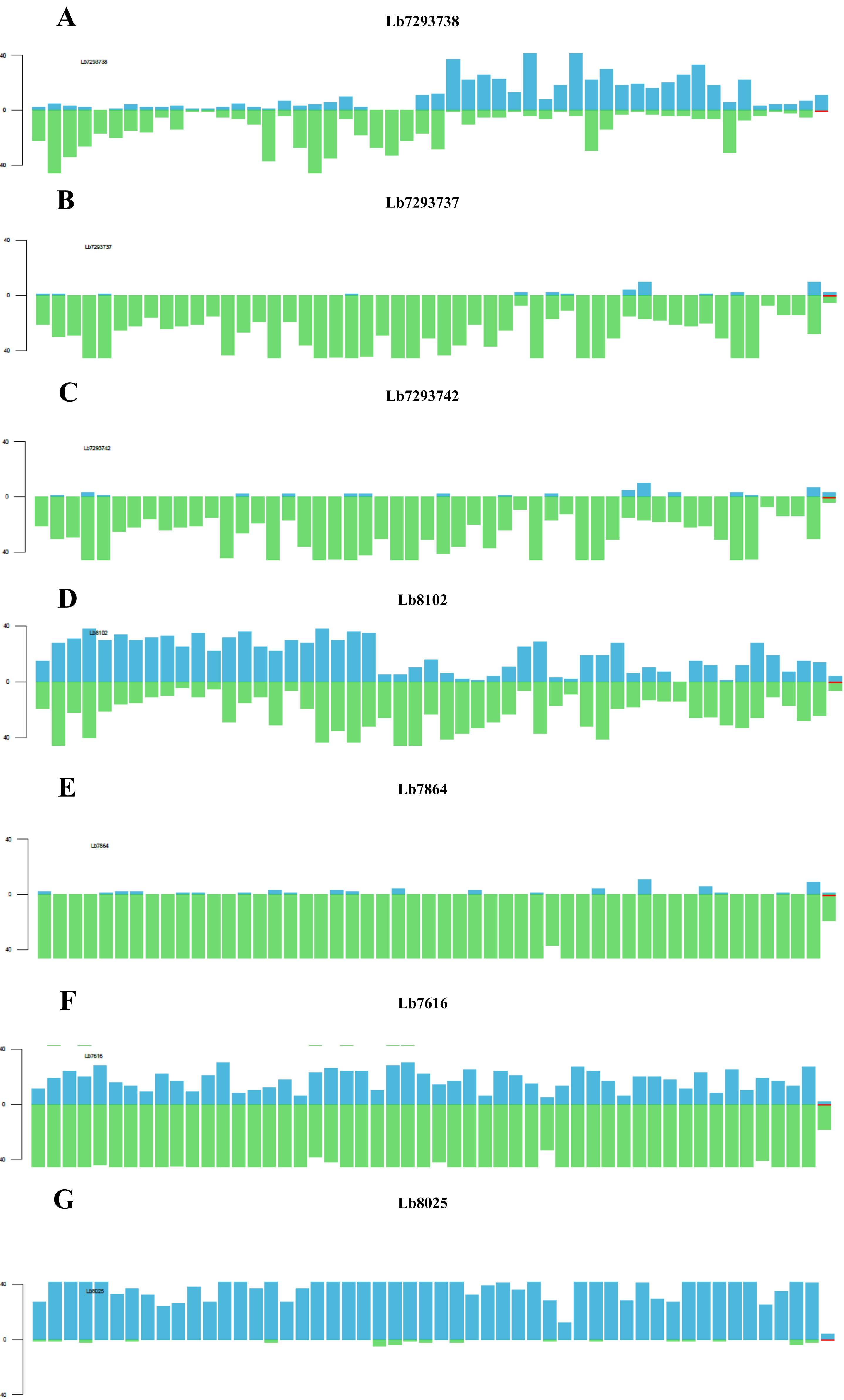
A
Lb7293738
B
Lb7293737
C
Lb7293742
D
Lb8102
E
Lb7864
F
Lb7616
G
Lb8025

Chr-7

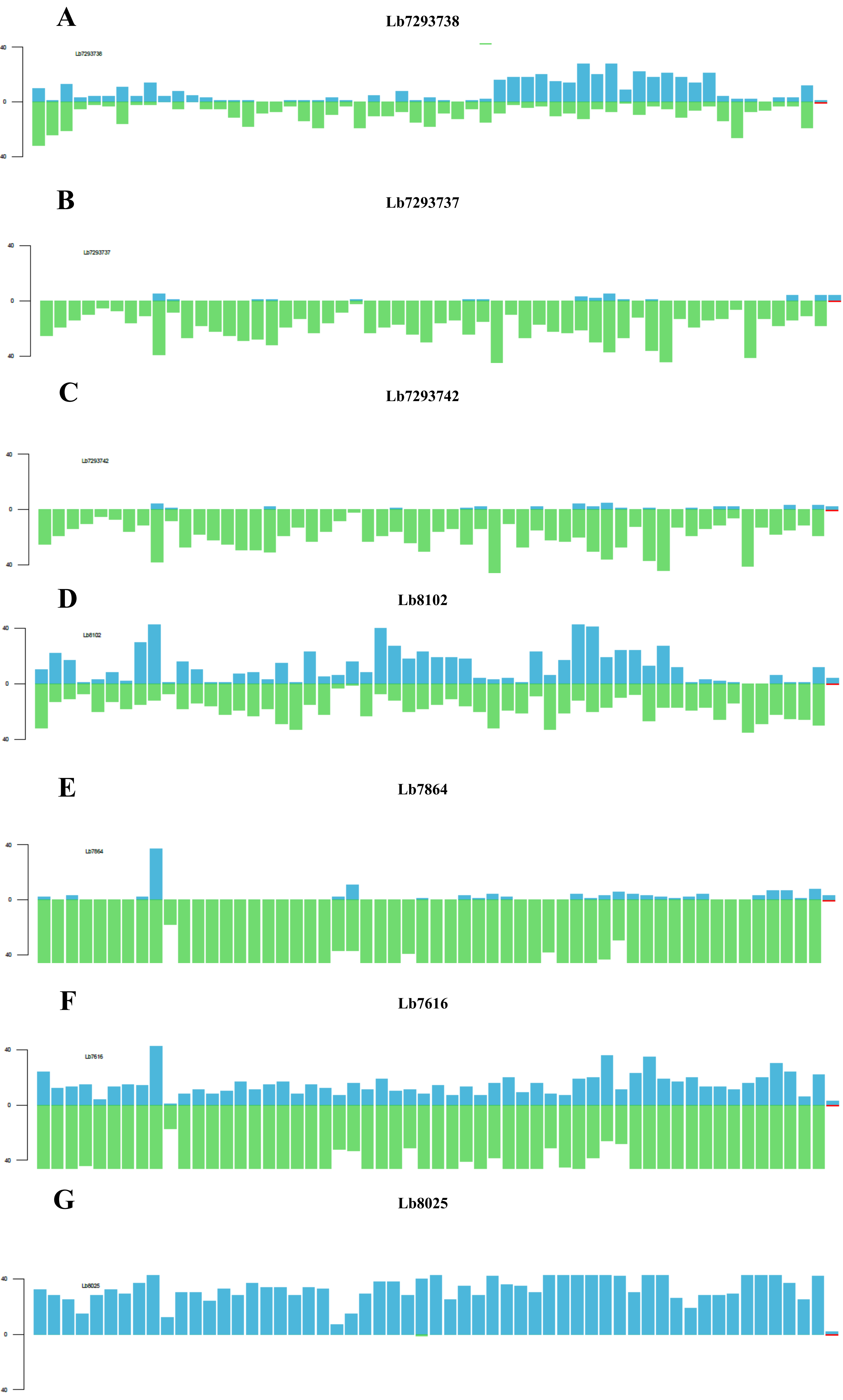
A
Lb7293738
40
0
40
B
Lb7293737
40
0
40
C
Lb7293742
40
0
40
D
Lb8102
40
0
40
E
Lb7864
40
0
40
F
Lb7616
40
0
40
G
Lb8025
40
0
40

Chr-8

A **Lb7293738**

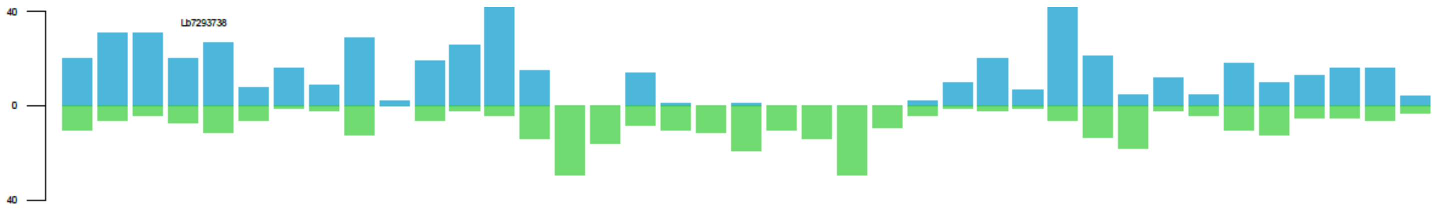

B **Lb7293737**

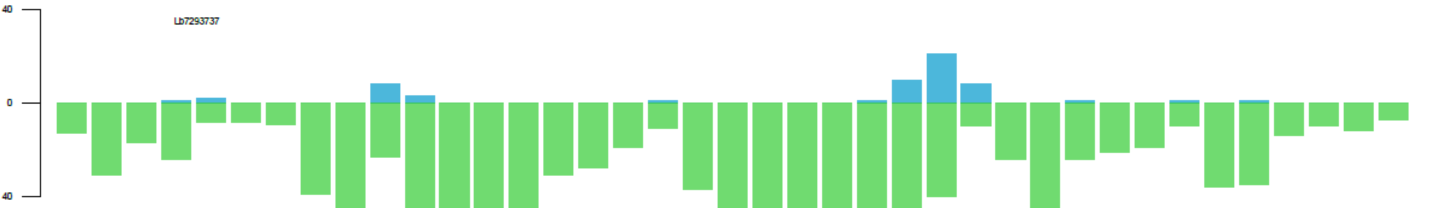

C **Lb7293742**

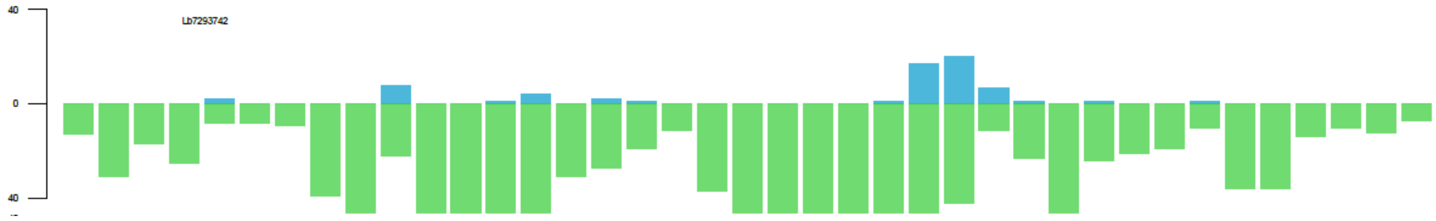

D **Lb8102**

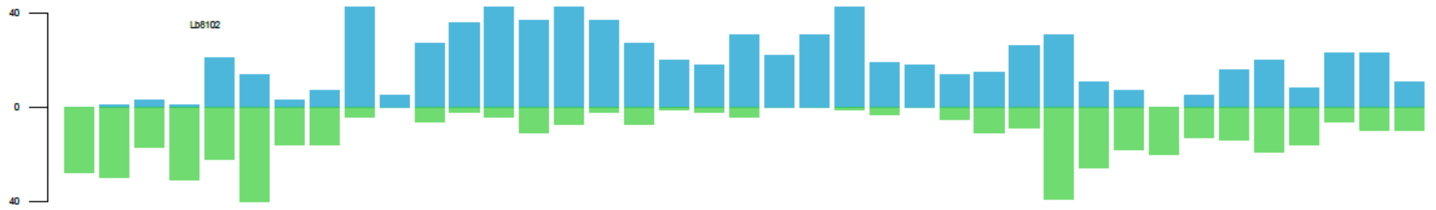

E **Lb7864**

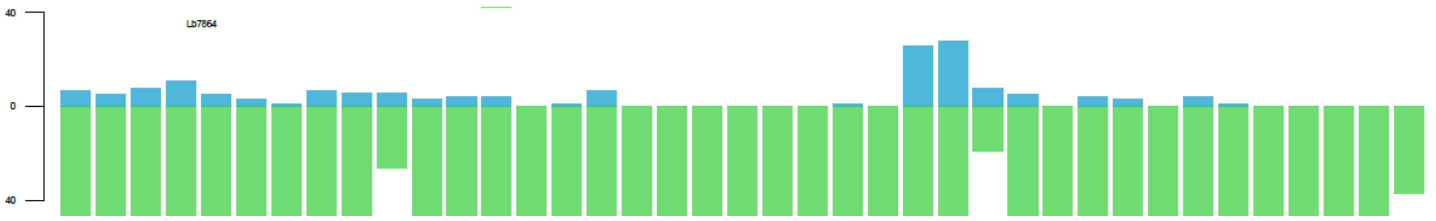

F **Lb7616**

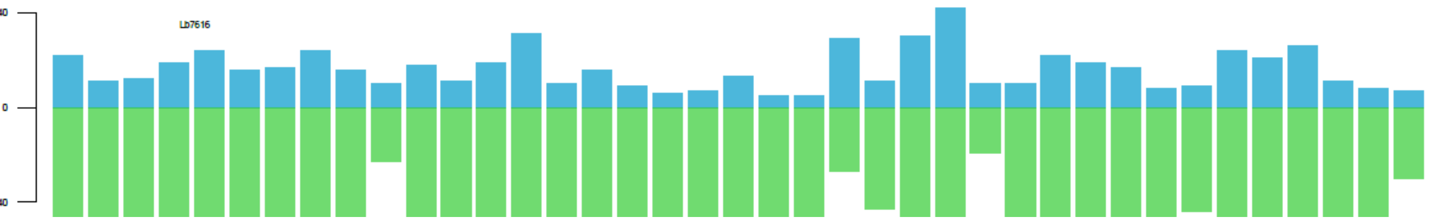

G **Lb8025**

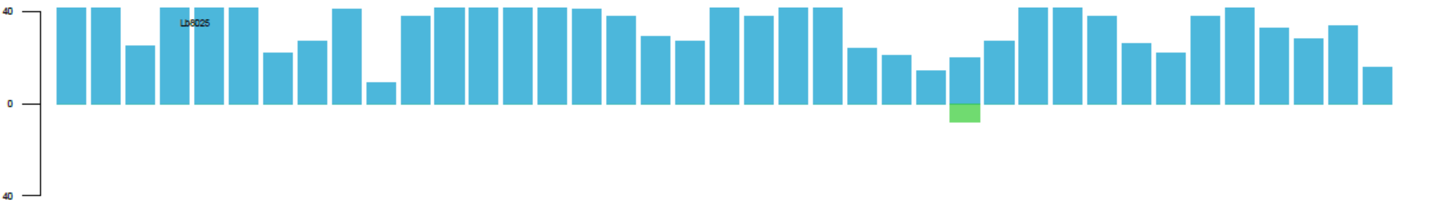

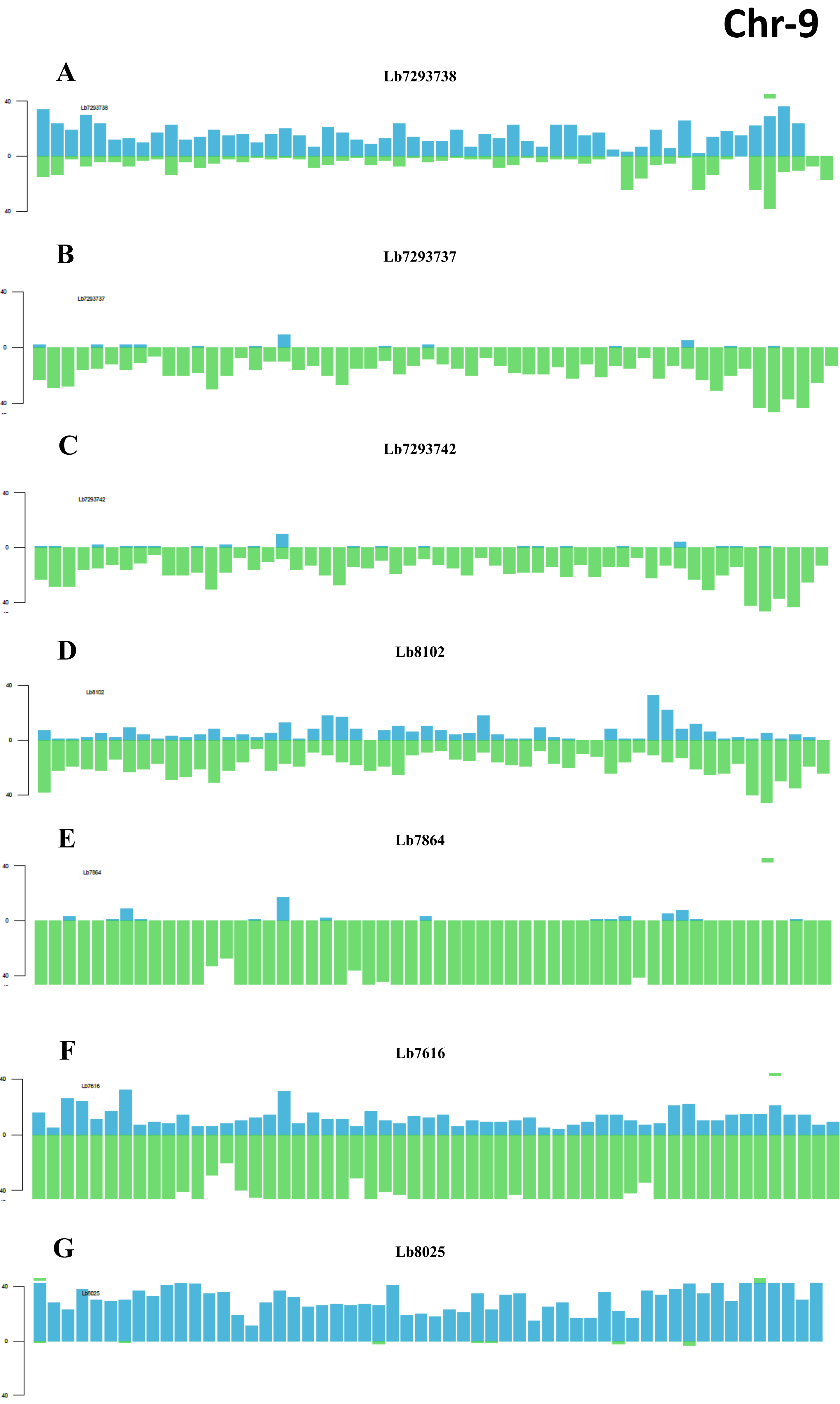

Chr-9
A
Lb7293738
B
Lb7293737
C
Lb7293742
D
Lb8102
E
Lb7864
F
Lb7616
G
Lb8025

Chr-10

A Lb7293738

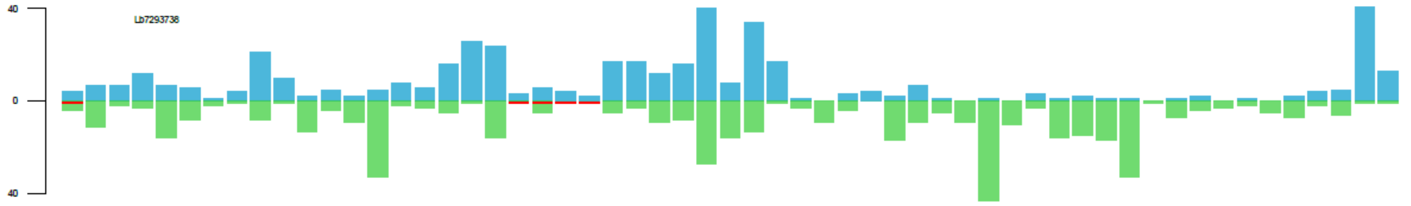

B Lb7293737

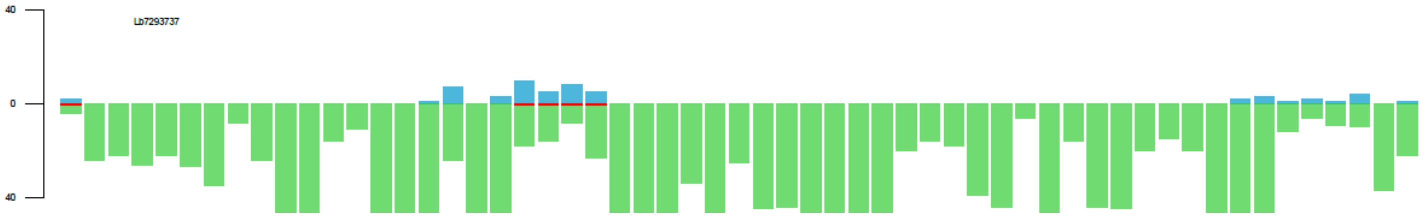

C Lb7293742

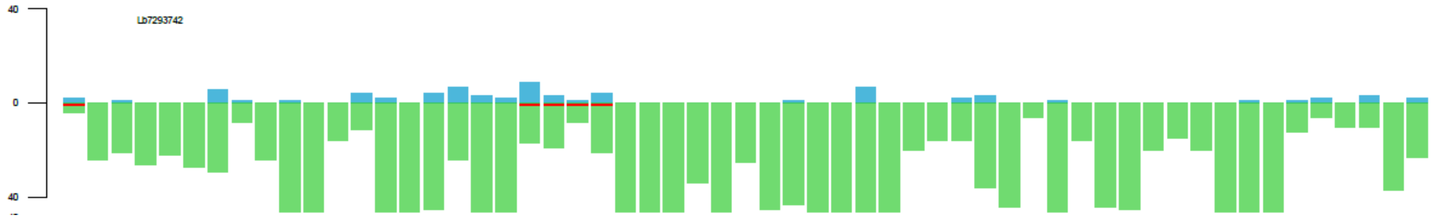

D Lb8102

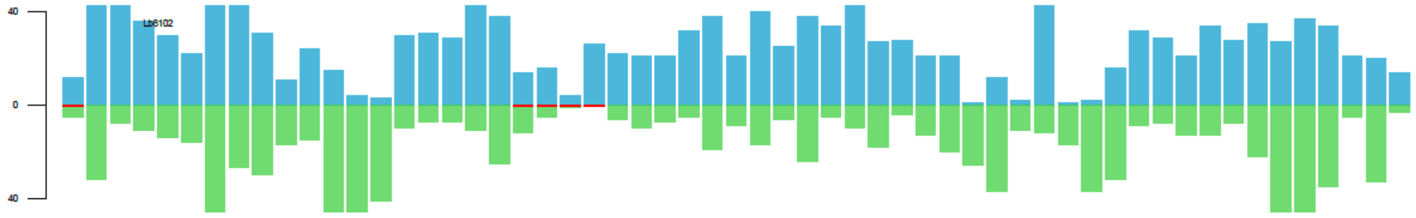

E Lb7864

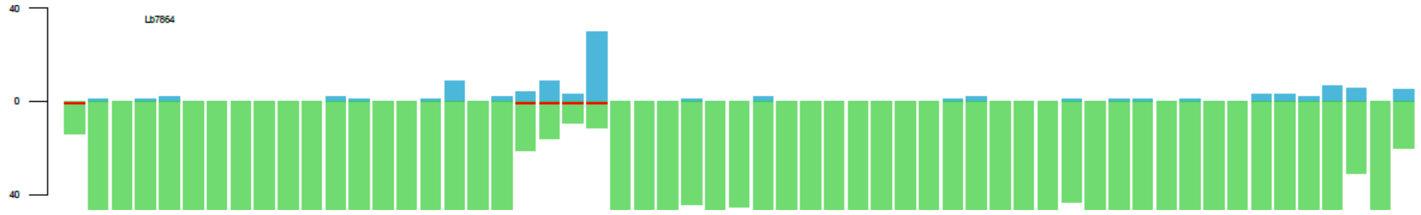

F Lb7616

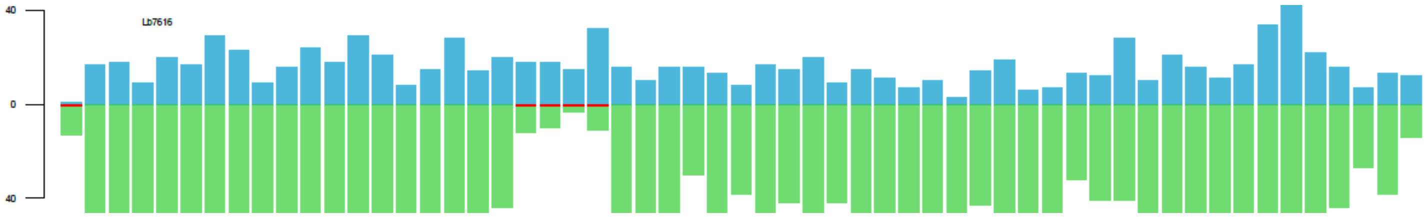

G Lb8025

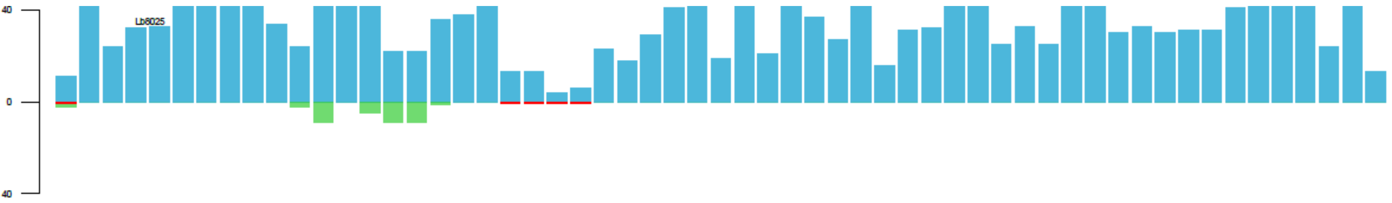

Chr-11

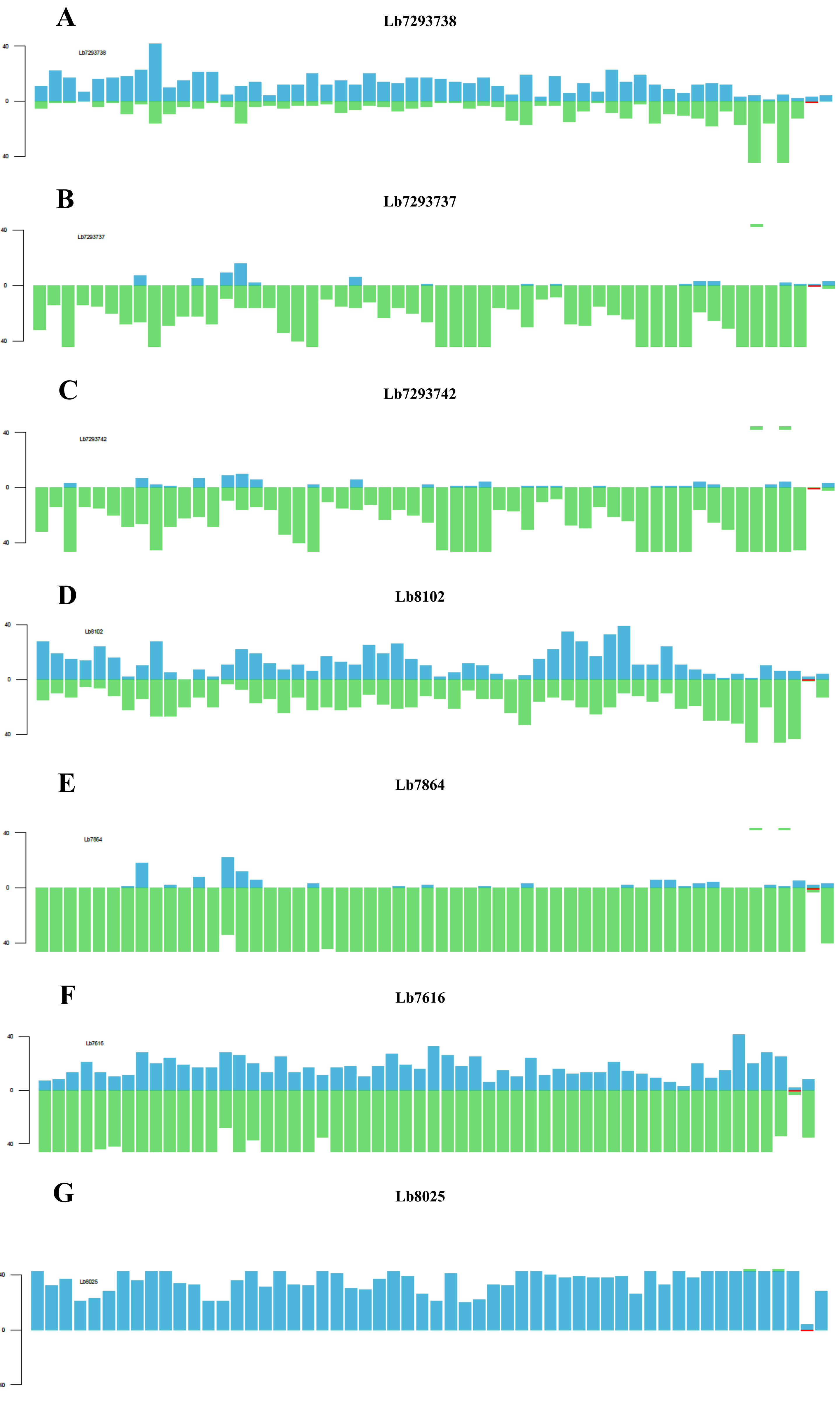
A
Lb7293738
B
Lb7293737
C
Lb7293742
D
Lb8102
E
Lb7864
F
Lb7616
G
Lb8025
40
0
40

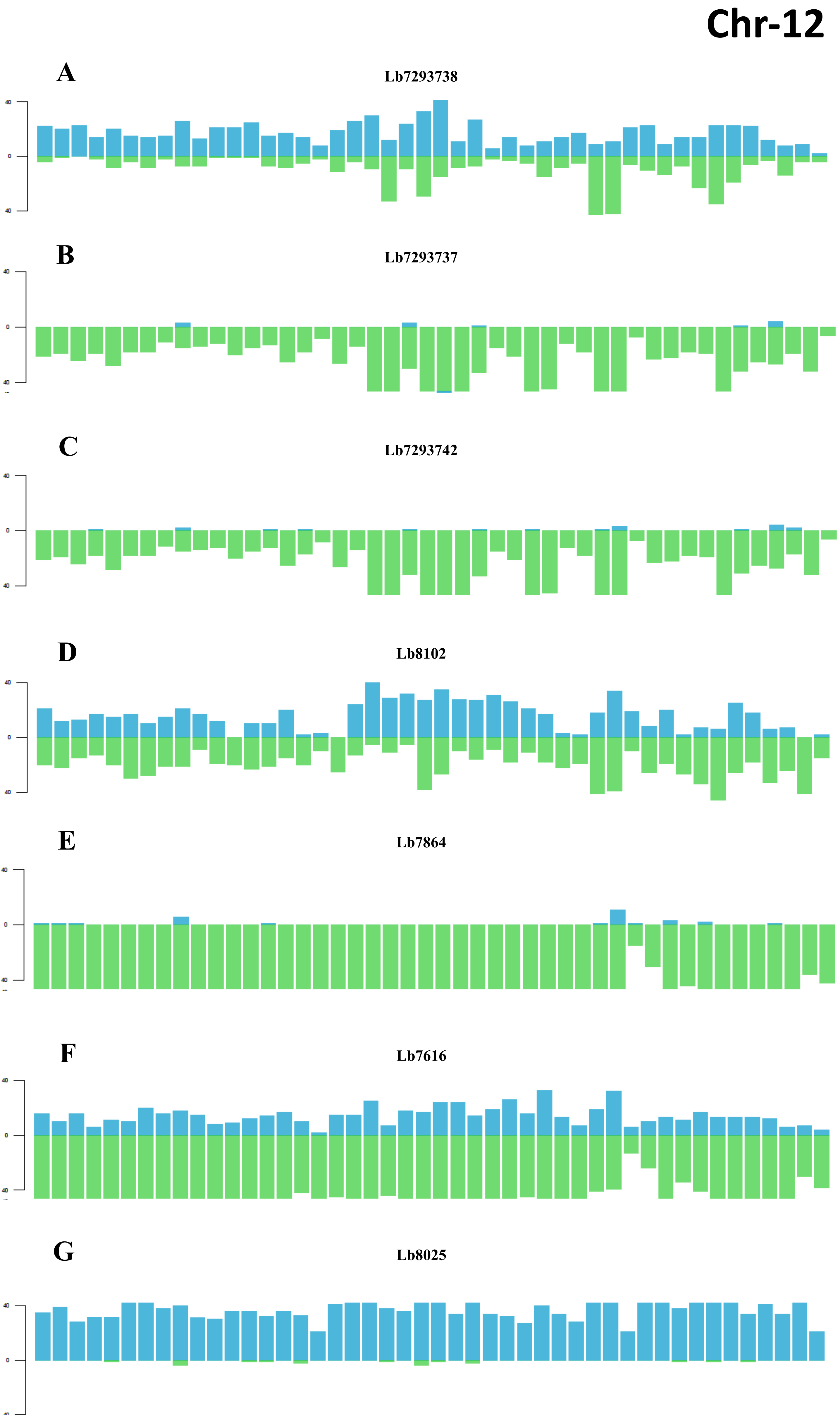
Chr-12
A
Lb7293738
B
Lb7293737
C
Lb7293742
D
Lb8102
E
Lb7864
F
Lb7616
G
Lb8025

Chr-13

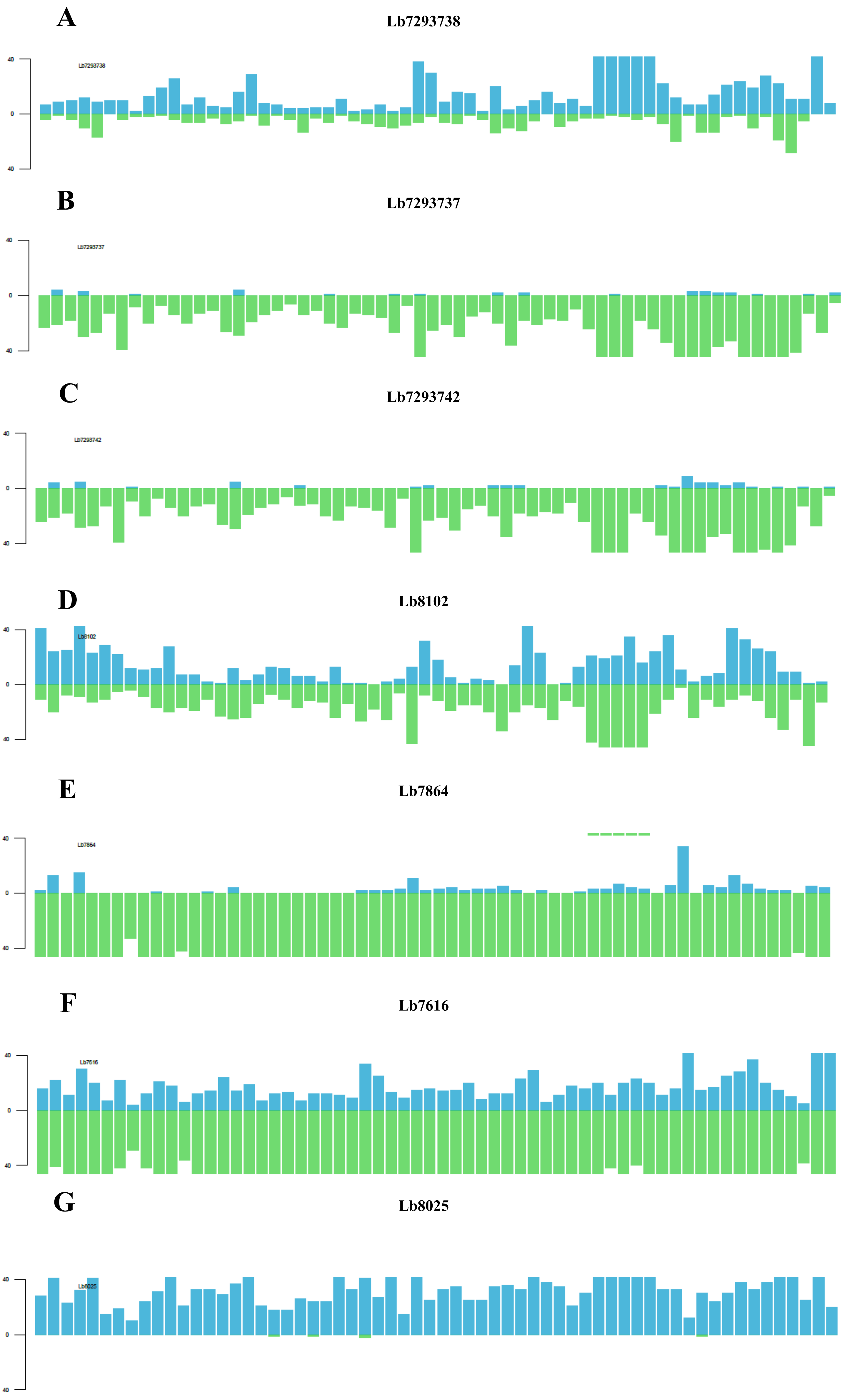
A
Lb7293738
B
Lb7293737
C
Lb7293742
D
Lb8102
E
Lb7864
F
Lb7616
G
Lb8025
40
0
40

Chr-14

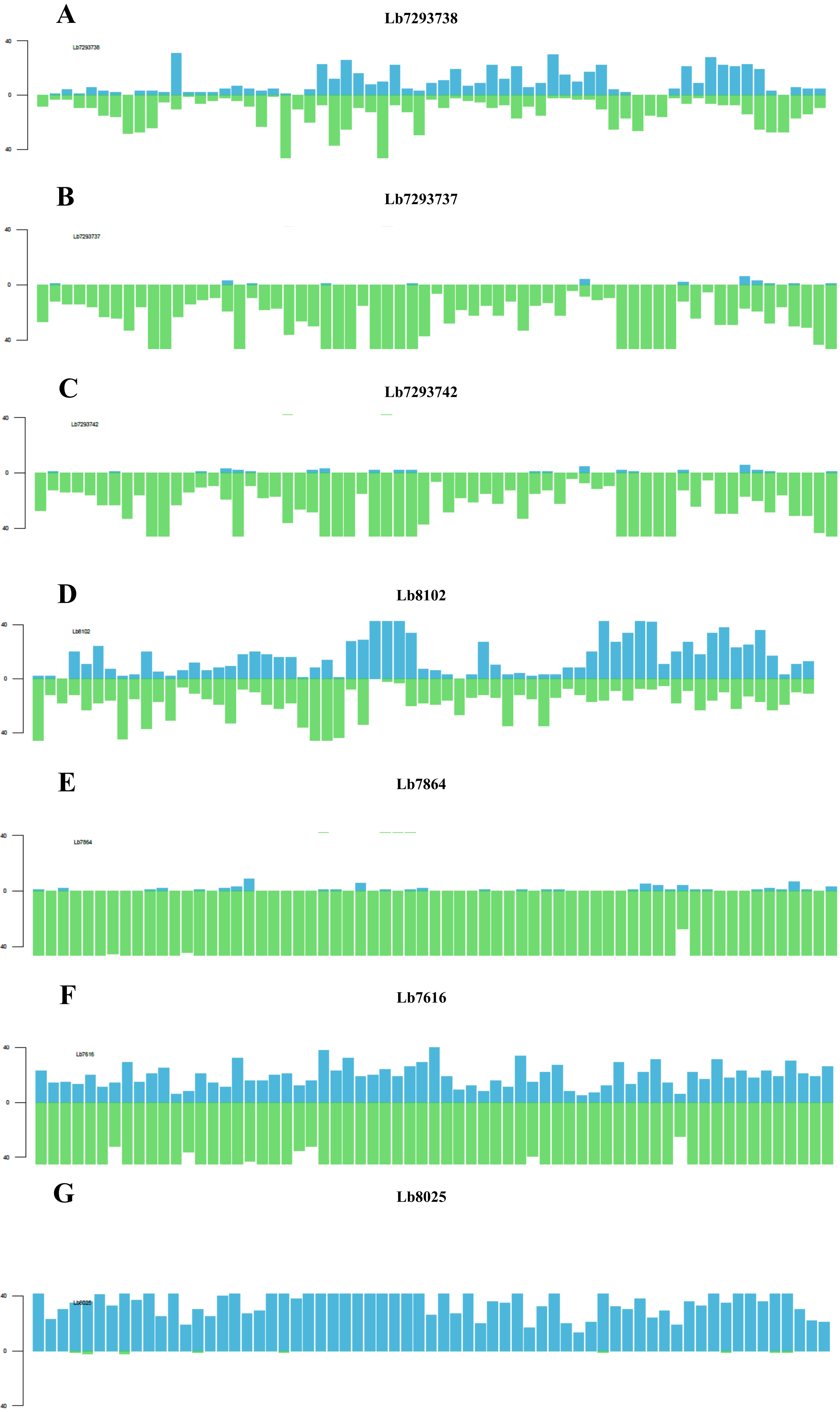
A
Lb7293738
B
Lb7293737
C
Lb7293742
D
Lb8102
E
Lb7864
F
Lb7616
G
Lb8025
40
0
40

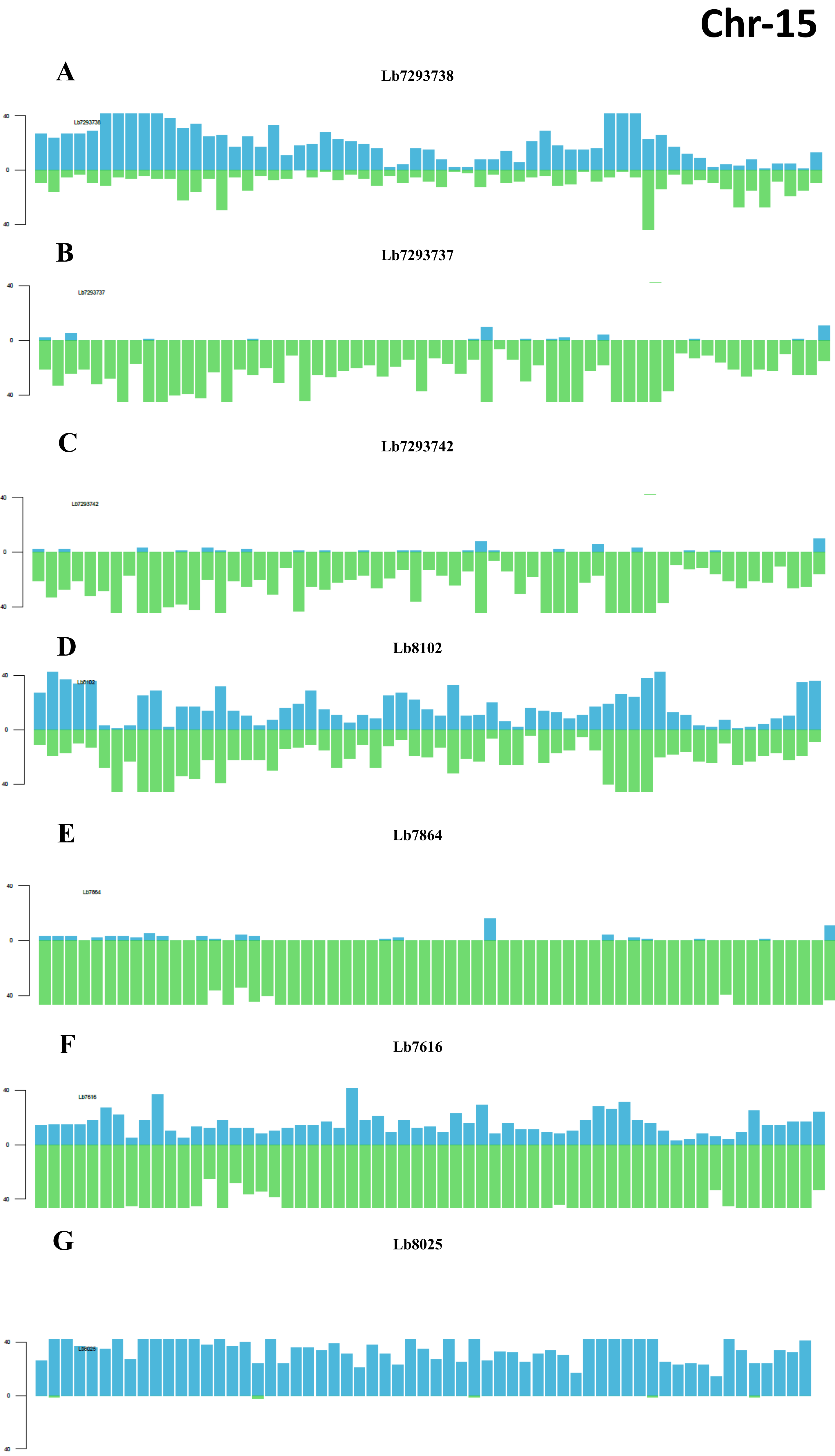
Chr-15
A
Lb7293738
40
0
40
B
Lb7293737
40
0
40
C
Lb7293742
40
0
40
D
Lb8102
40
0
40
E
Lb7864
40
0
40
F
Lb7616
40
0
40
G
Lb8025
40
0
40

Chr-16

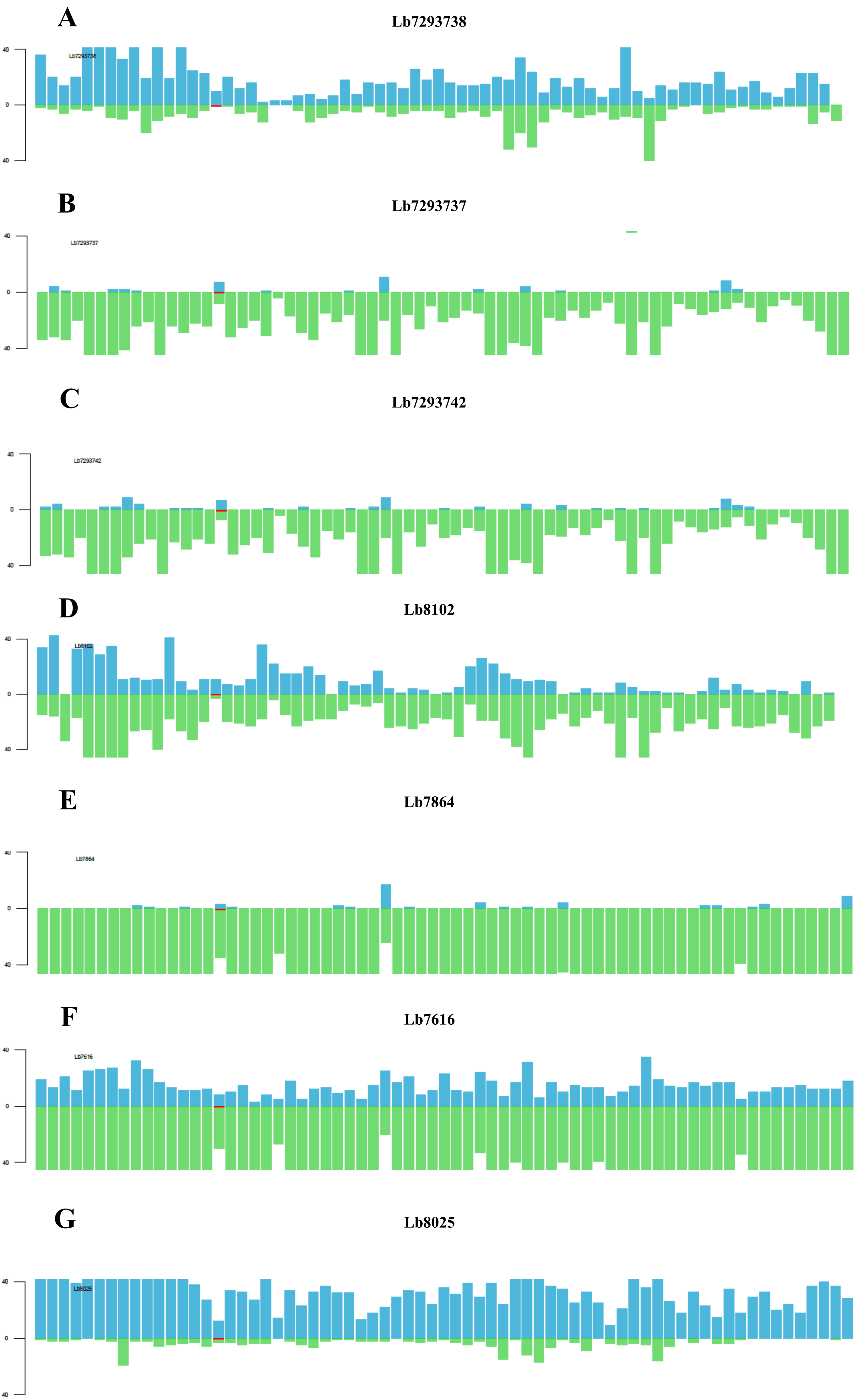
A
Lb7293738
B
Lb7293737
C
Lb7293742
D
Lb8102
E
Lb7864
F
Lb7616
G
Lb8025
40
0
40

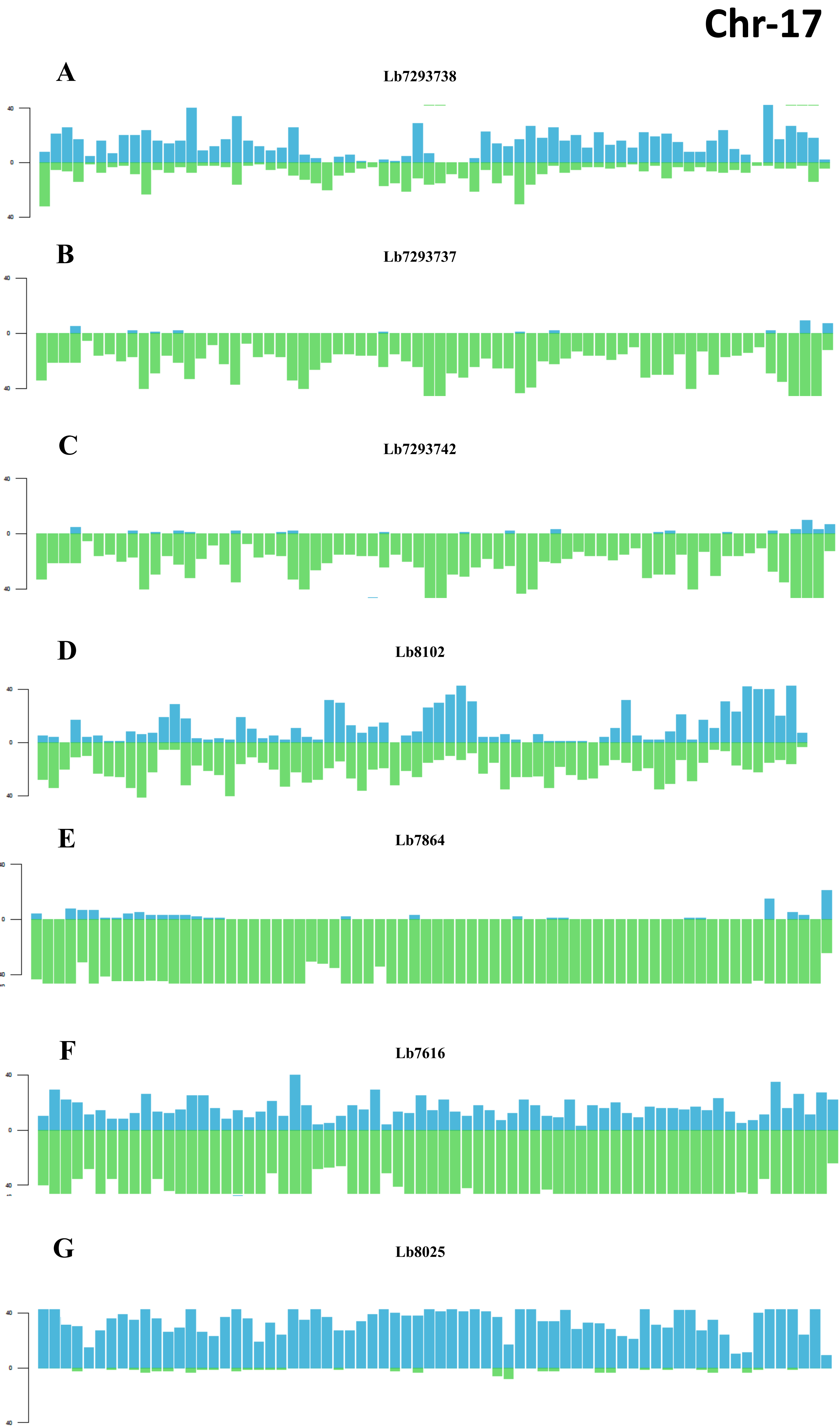
Chr-17
A
Lb7293738
B
Lb7293737
C
Lb7293742
D
Lb8102
E
Lb7864
F
Lb7616
G
Lb8025

Chr-18

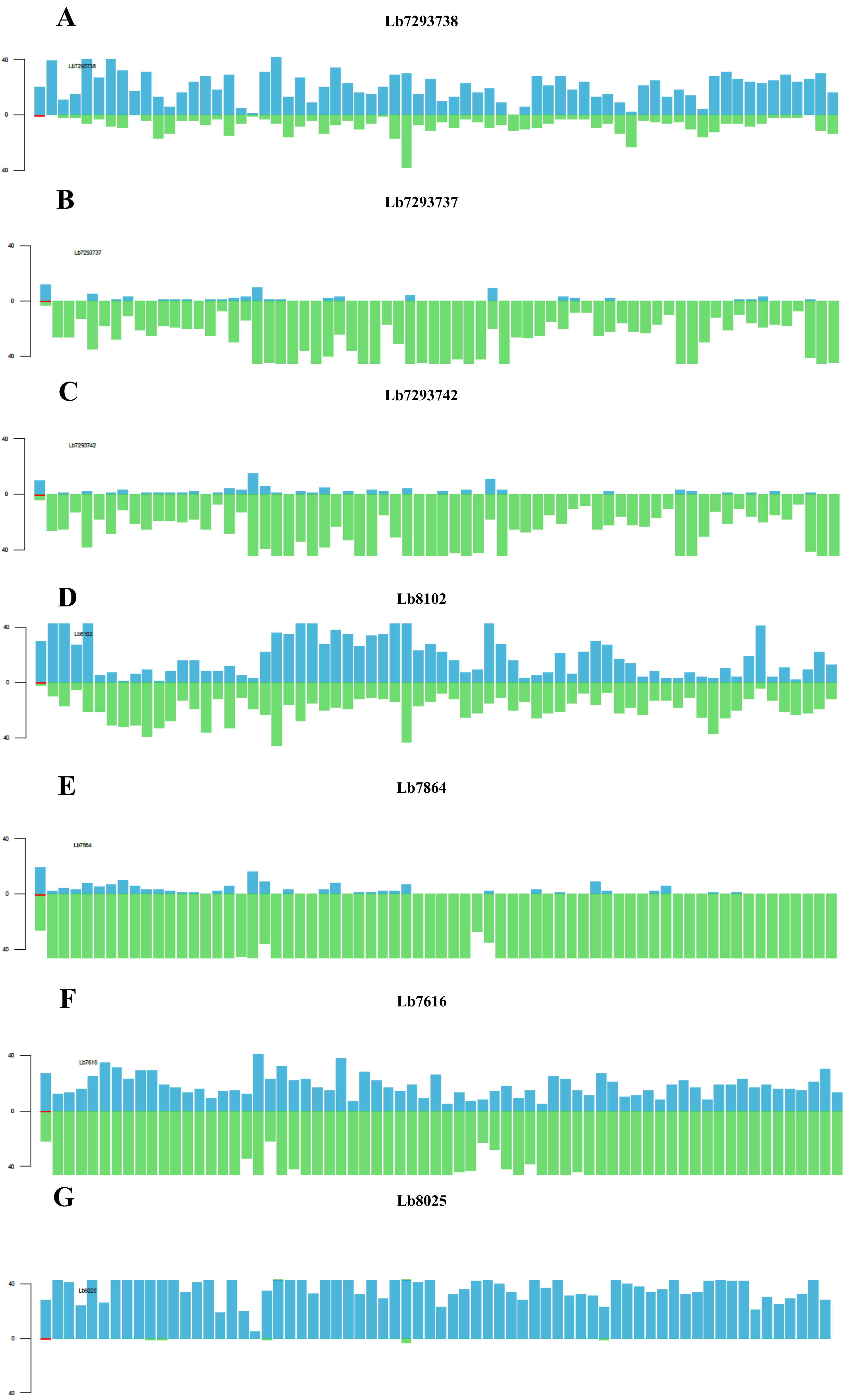
A
Lb7293738
B
Lb7293737
C
Lb7293742
D
Lb8102
E
Lb7864
F
Lb7616
G
Lb8025
40
0
40

Chr-19

A Lb7293738

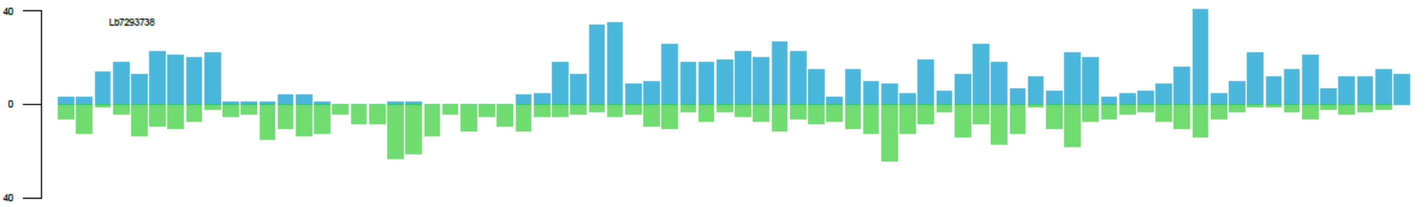

B Lb7293737

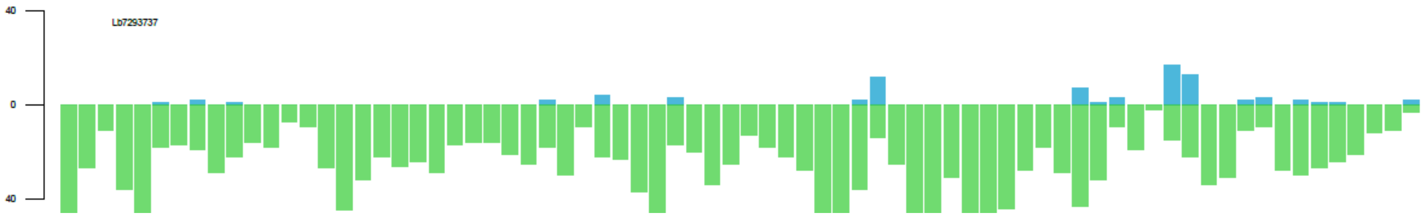

C Lb7293742

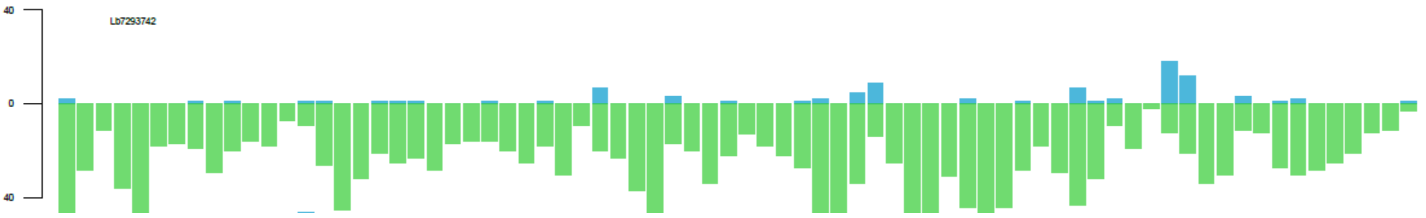

D Lb8102

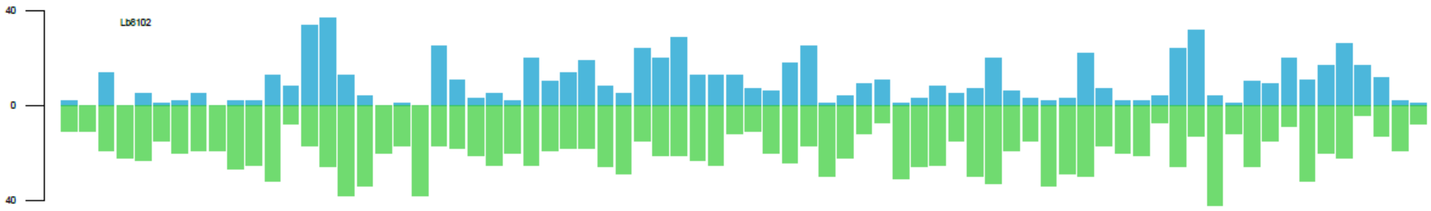

E Lb7864

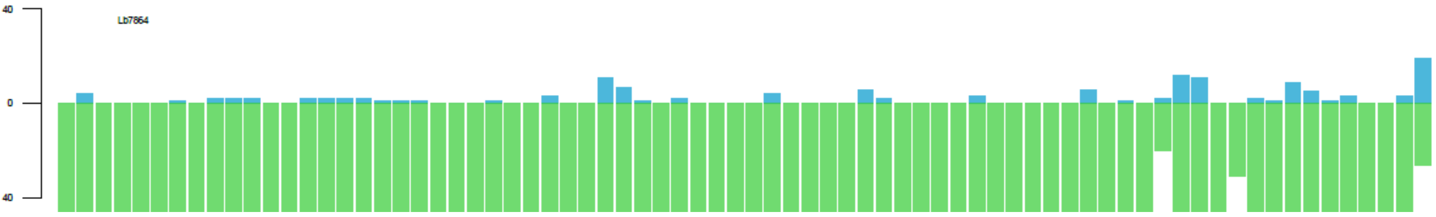

F Lb7616

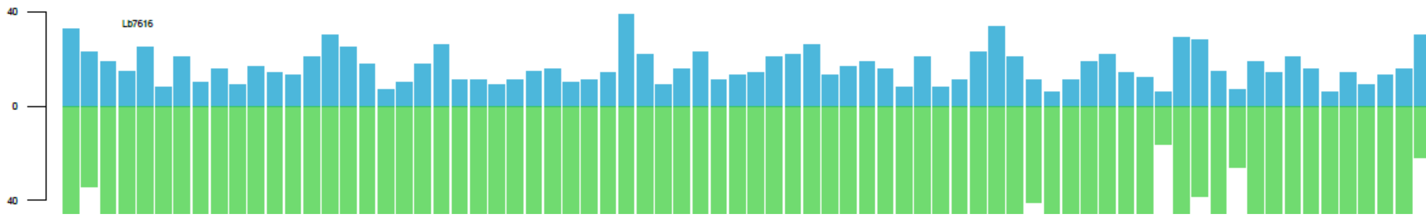

G Lb8025

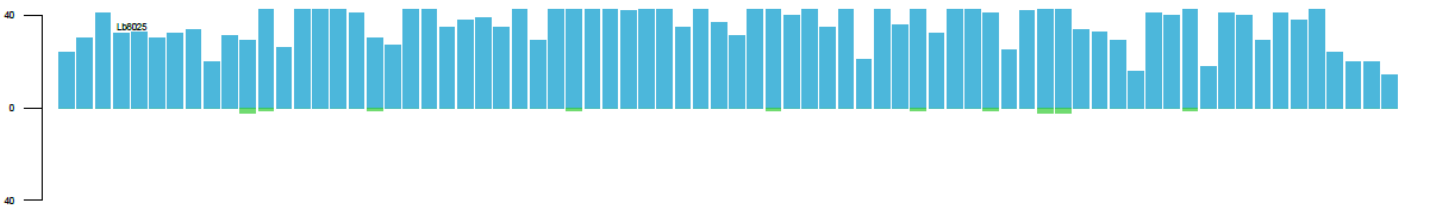

Chr-20

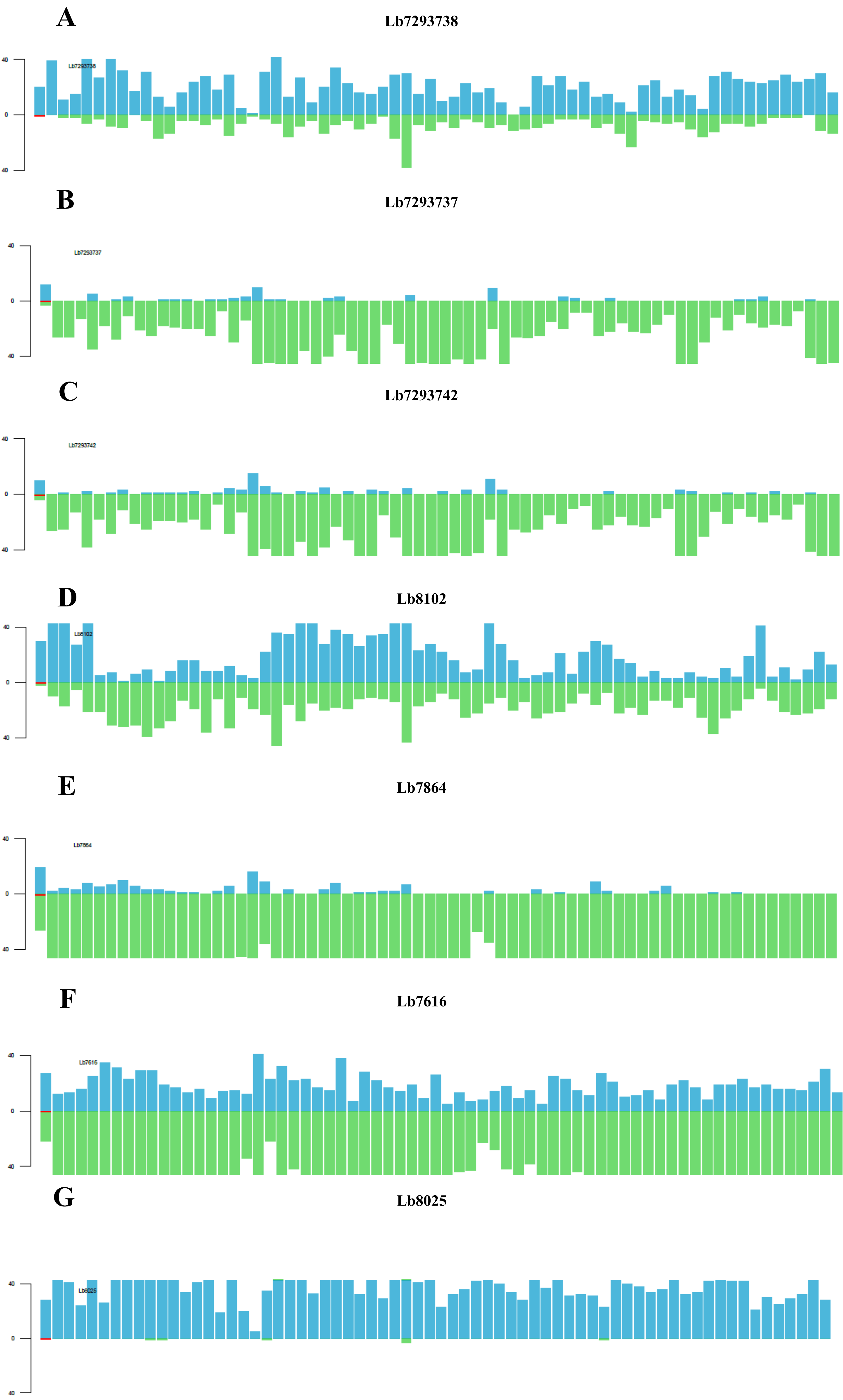
A
Lb7293738
40
Lb7293738
0
40
B
Lb7293737
40
Lb7293737
0
40
C
Lb7293742
40
Lb7293742
0
40
D
Lb8102
40
Lb8102
0
40
E
Lb7864
40
Lb7864
0
40
F
Lb7616
40
Lb7616
0
40
G
Lb8025
40
Lb8025
0
40

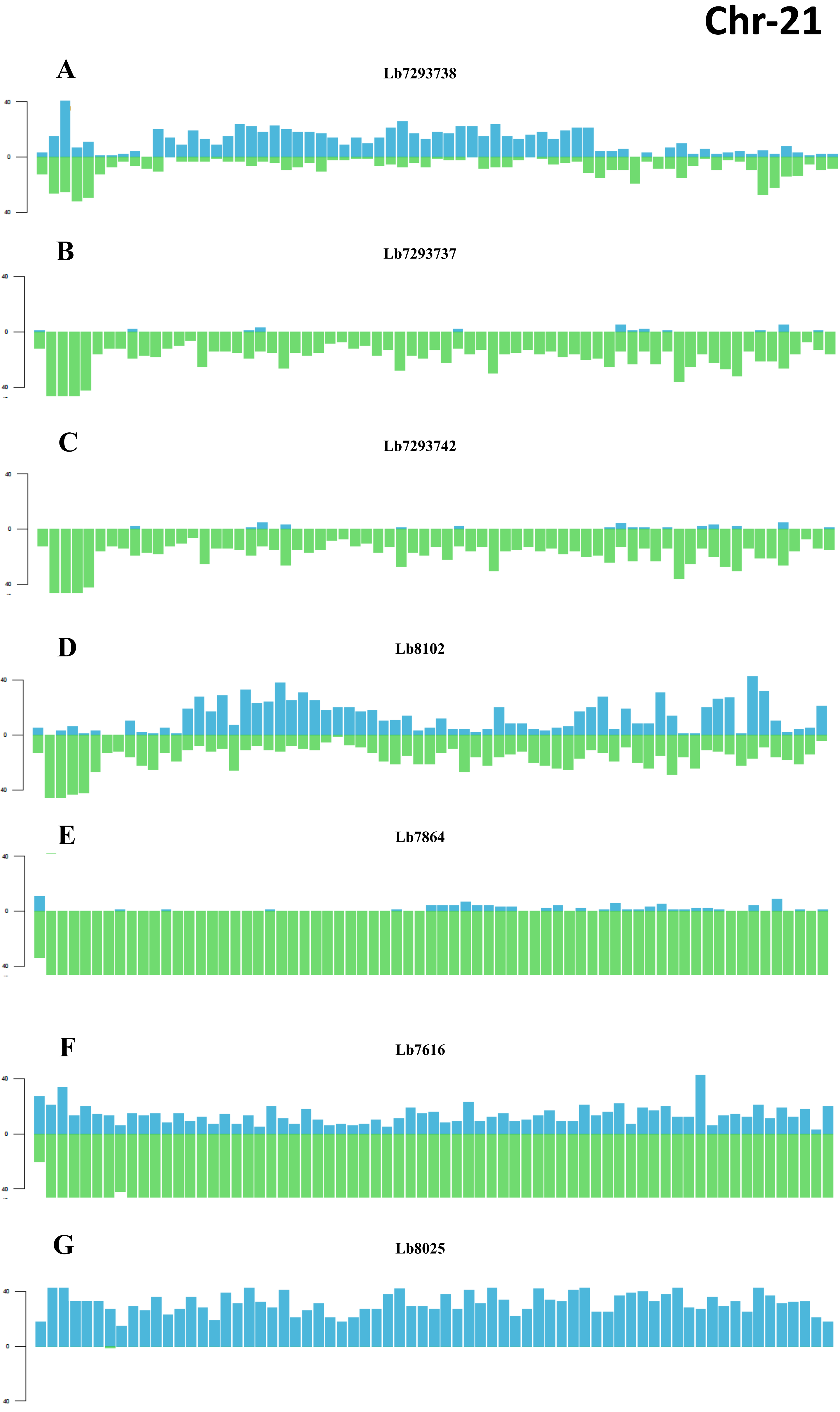
Chr-21
A
Lb7293738
B
Lb7293737
C
Lb7293742
D
Lb8102
E
Lb7864
F
Lb7616
G
Lb8025
40
0
40

Chr-22

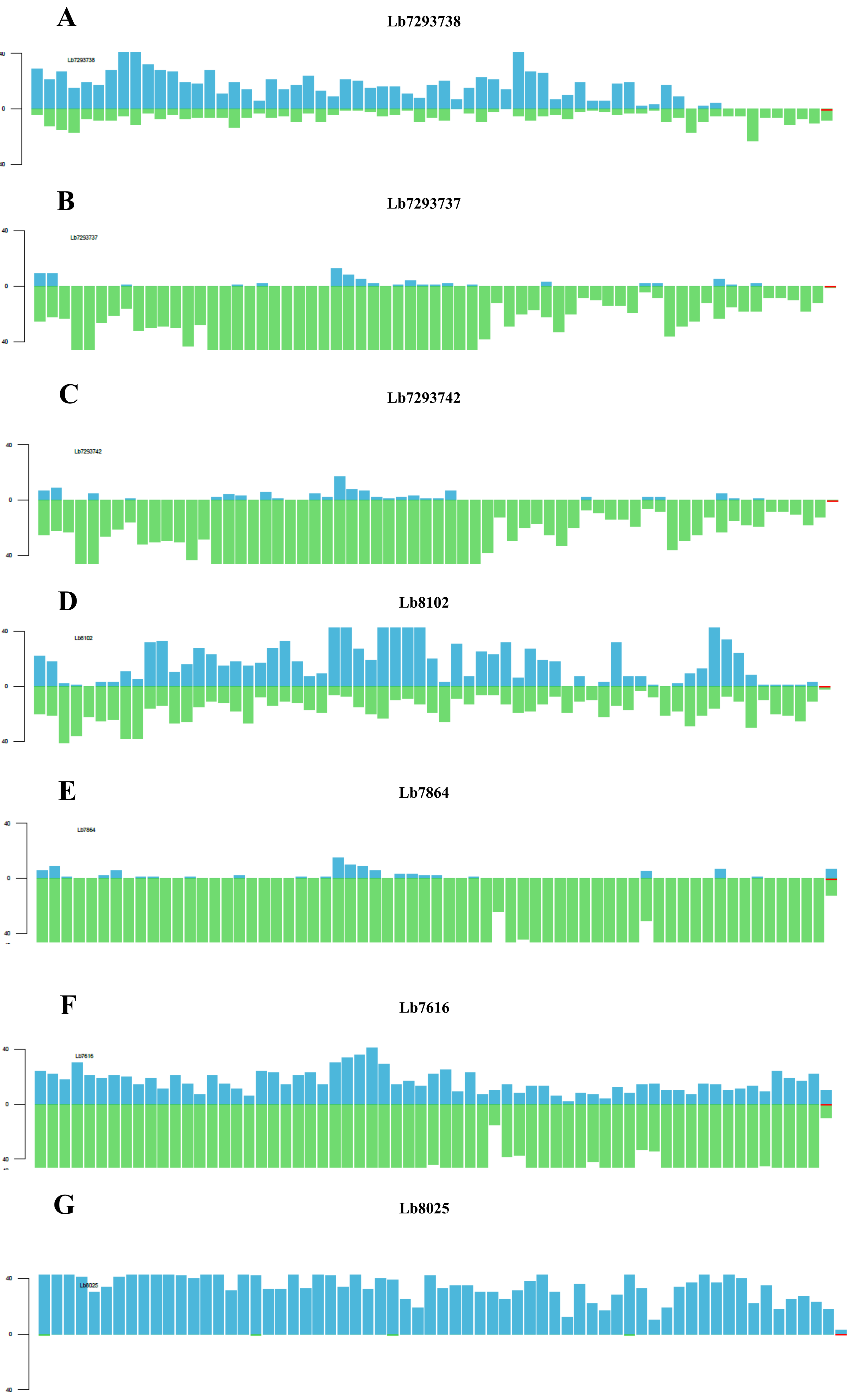
A
Lb7293738
B
Lb7293737
C
Lb7293742
D
Lb8102
E
Lb7864
F
Lb7616
G
Lb8025

Chr-23

A Lb7293738

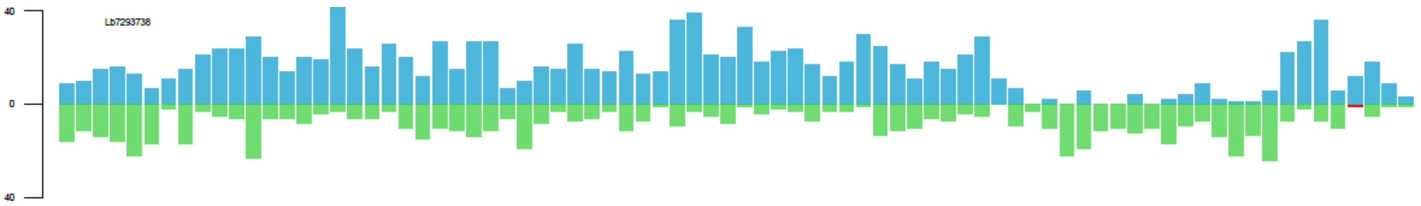

B Lb7293737

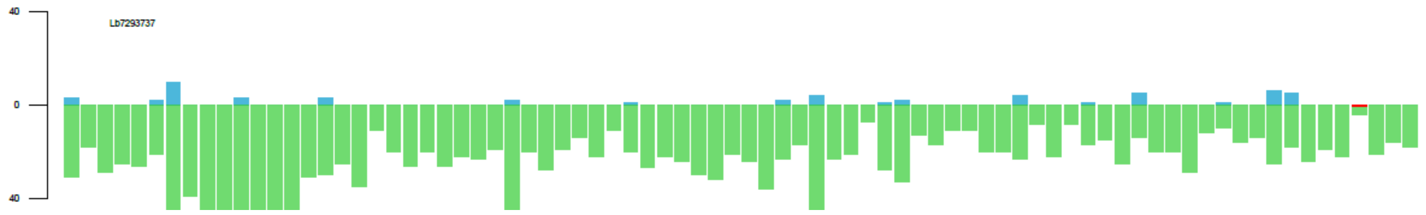

C Lb7293742

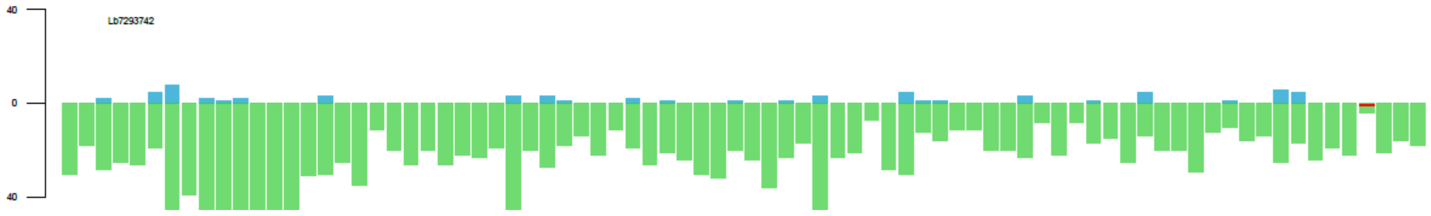

D Lb8102

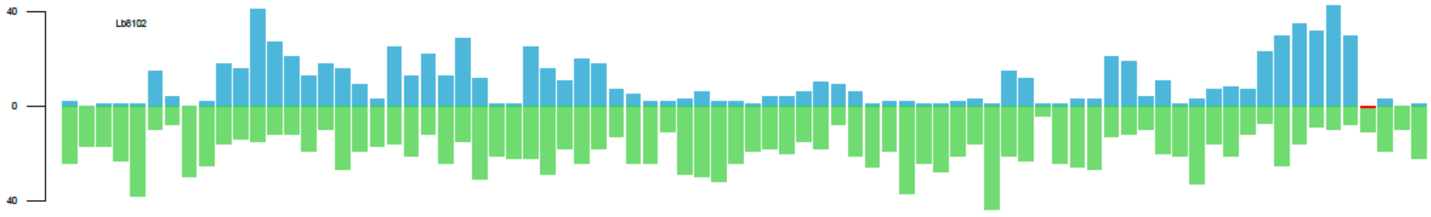

E Lb7864

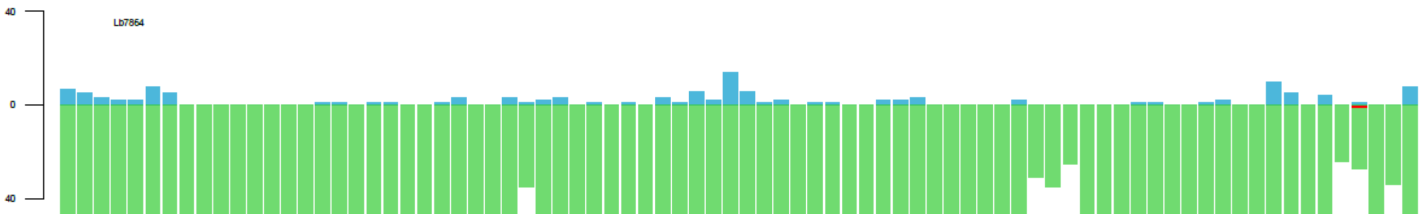

F Lb7616

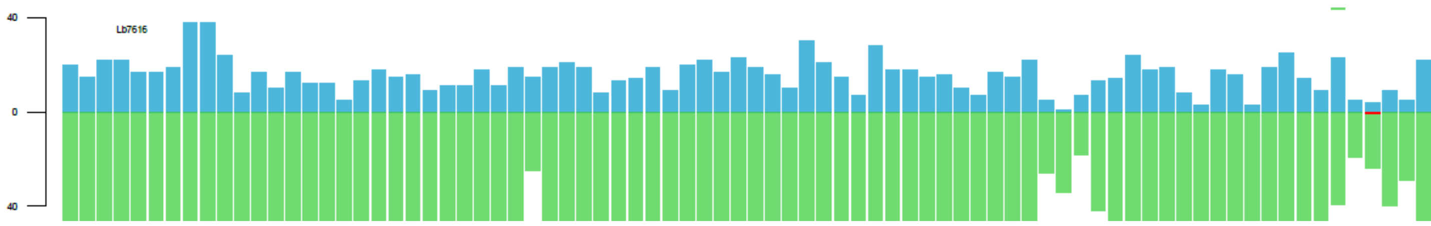

G Lb8025

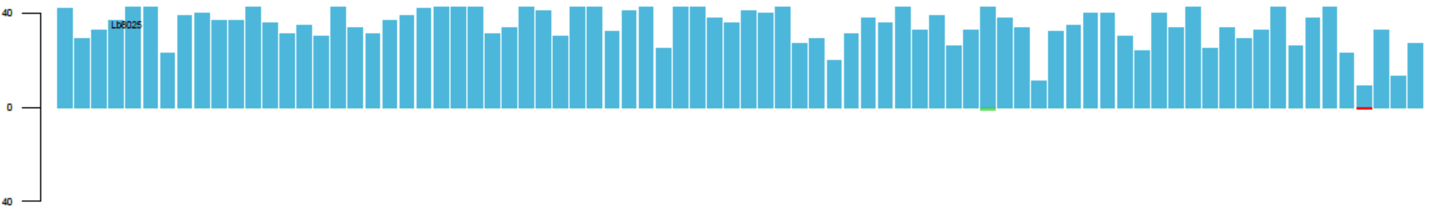

Chr-24

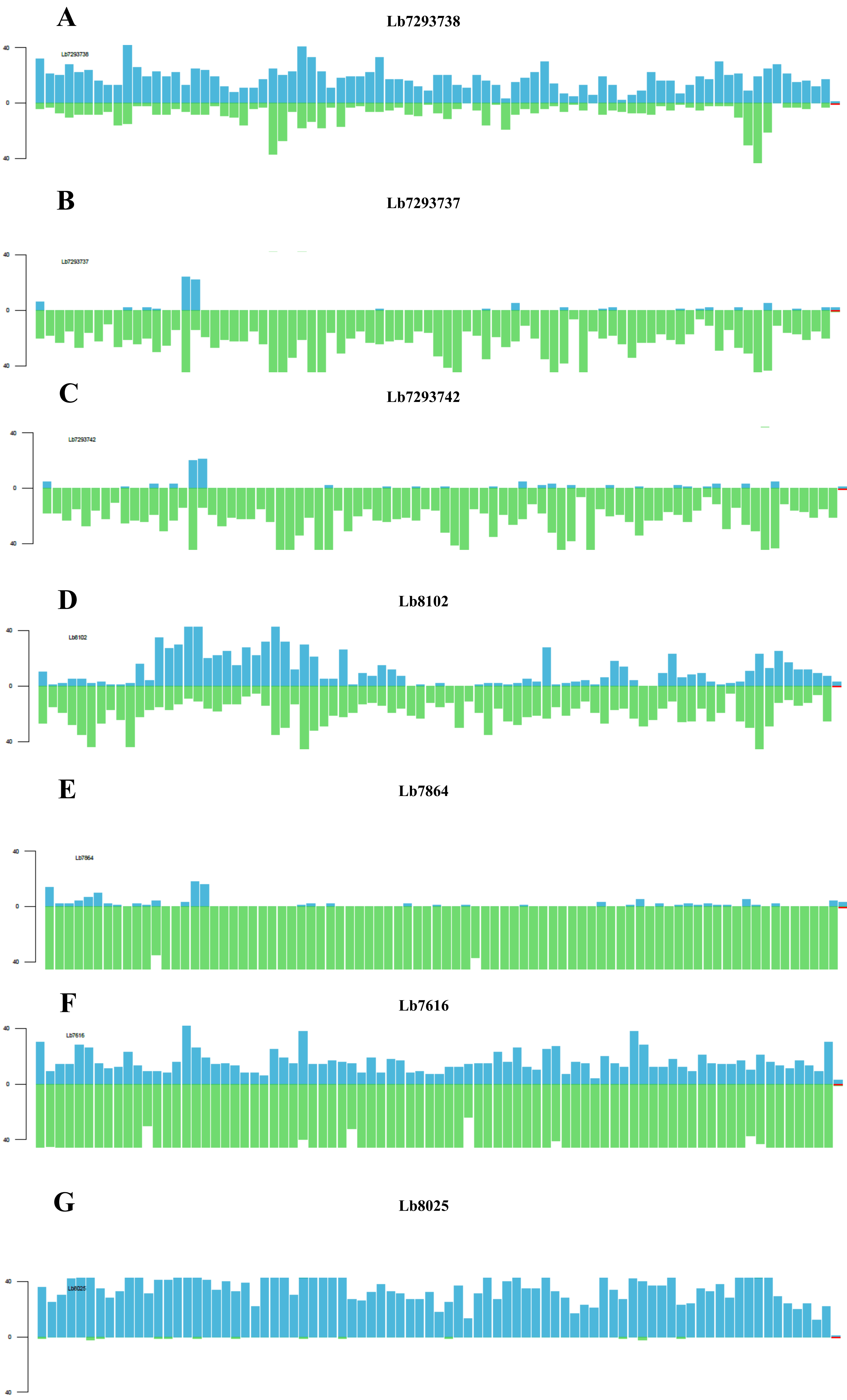

A
Lb7293738
B
Lb7293737
C
Lb7293742
D
Lb8102
E
Lb7864
F
Lb7616
G
Lb8025

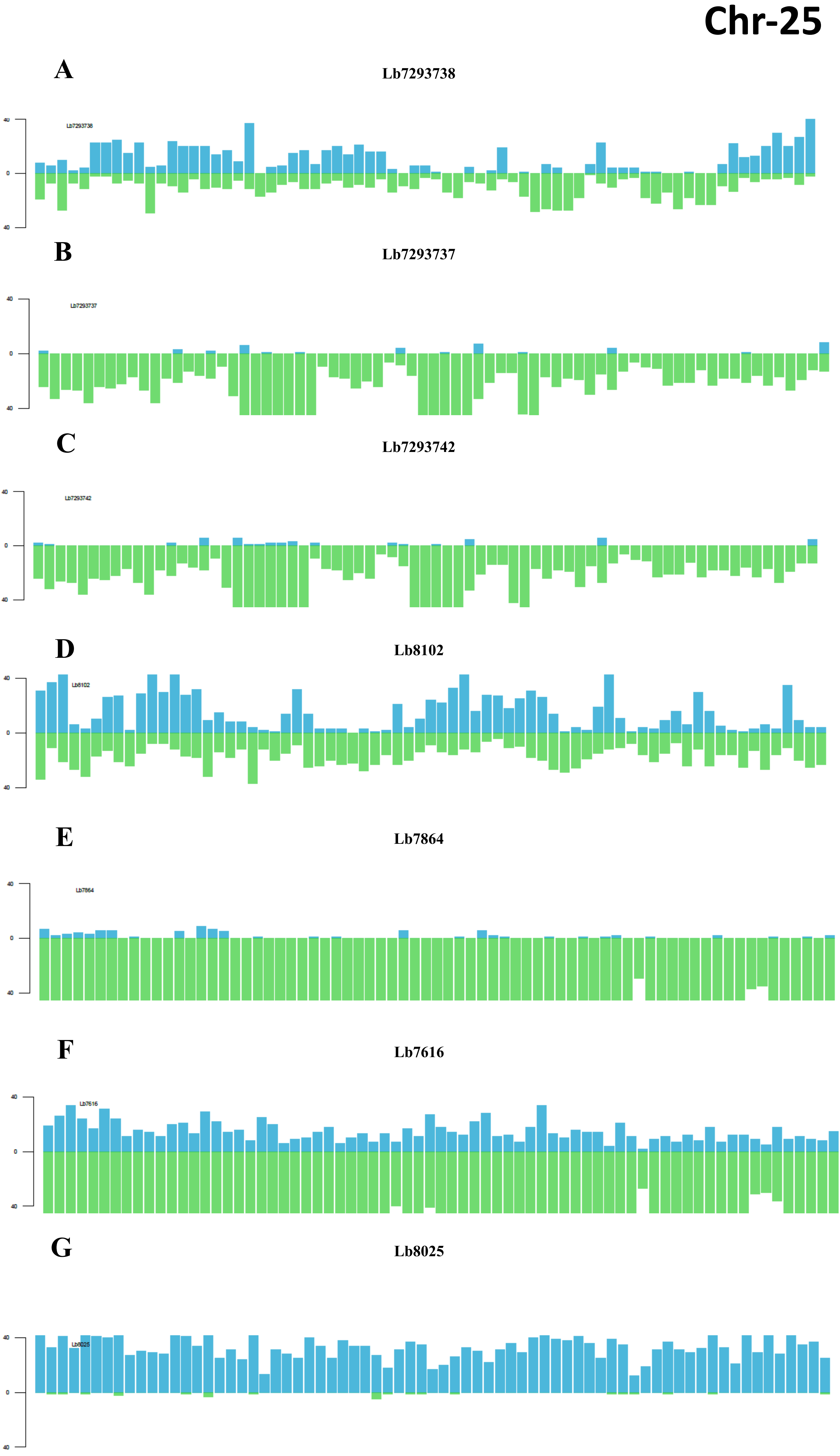
Chr-25
A
Lb7293738
B
Lb7293737
C
Lb7293742
D
Lb8102
E
Lb7864
F
Lb7616
G
Lb8025
40
0
40

Chr-26

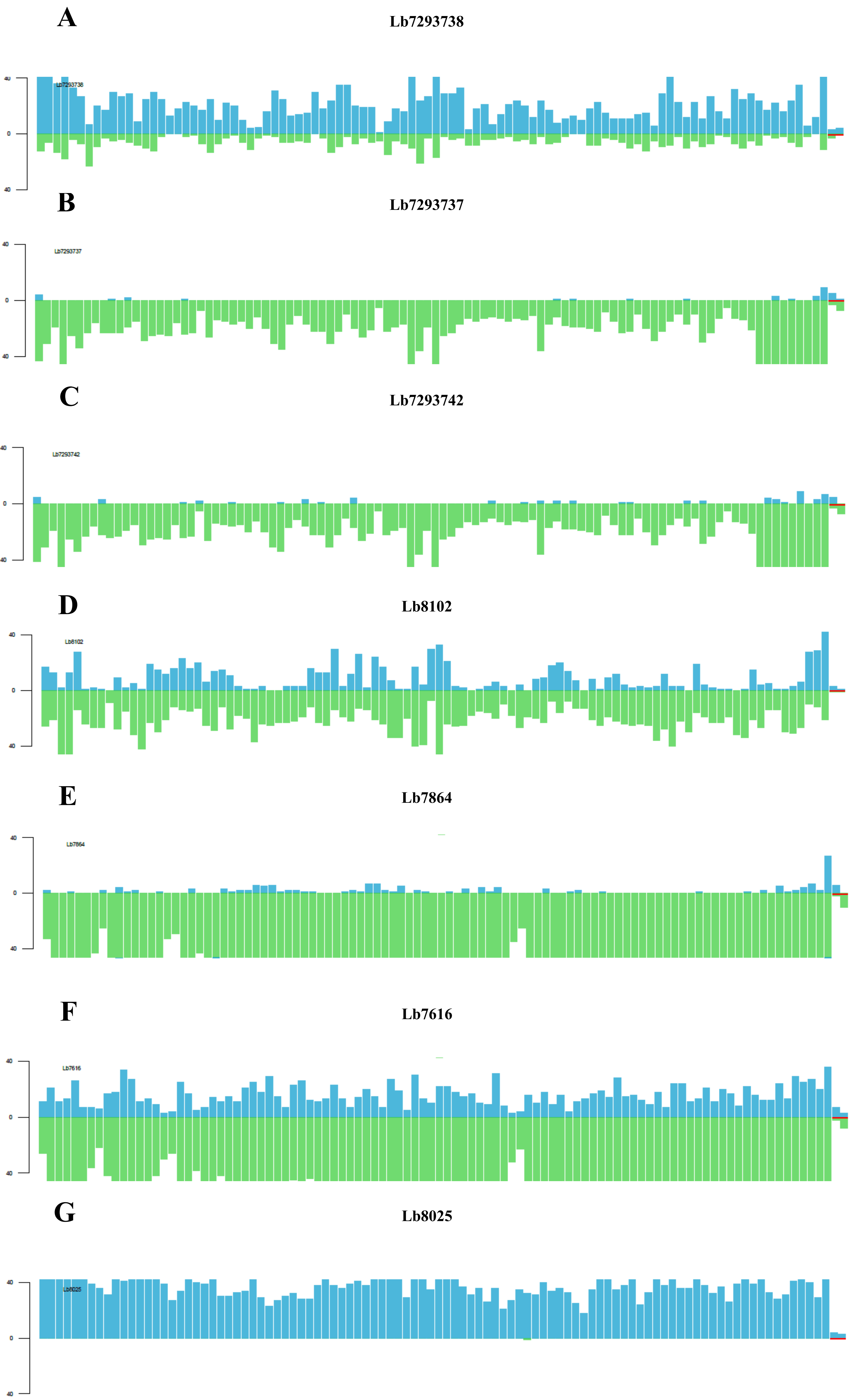
A
Lb7293738
40
0
40
B
Lb7293737
C
Lb7293742
D
Lb8102
E
Lb7864
F
Lb7616
G
Lb8025

Chr-27

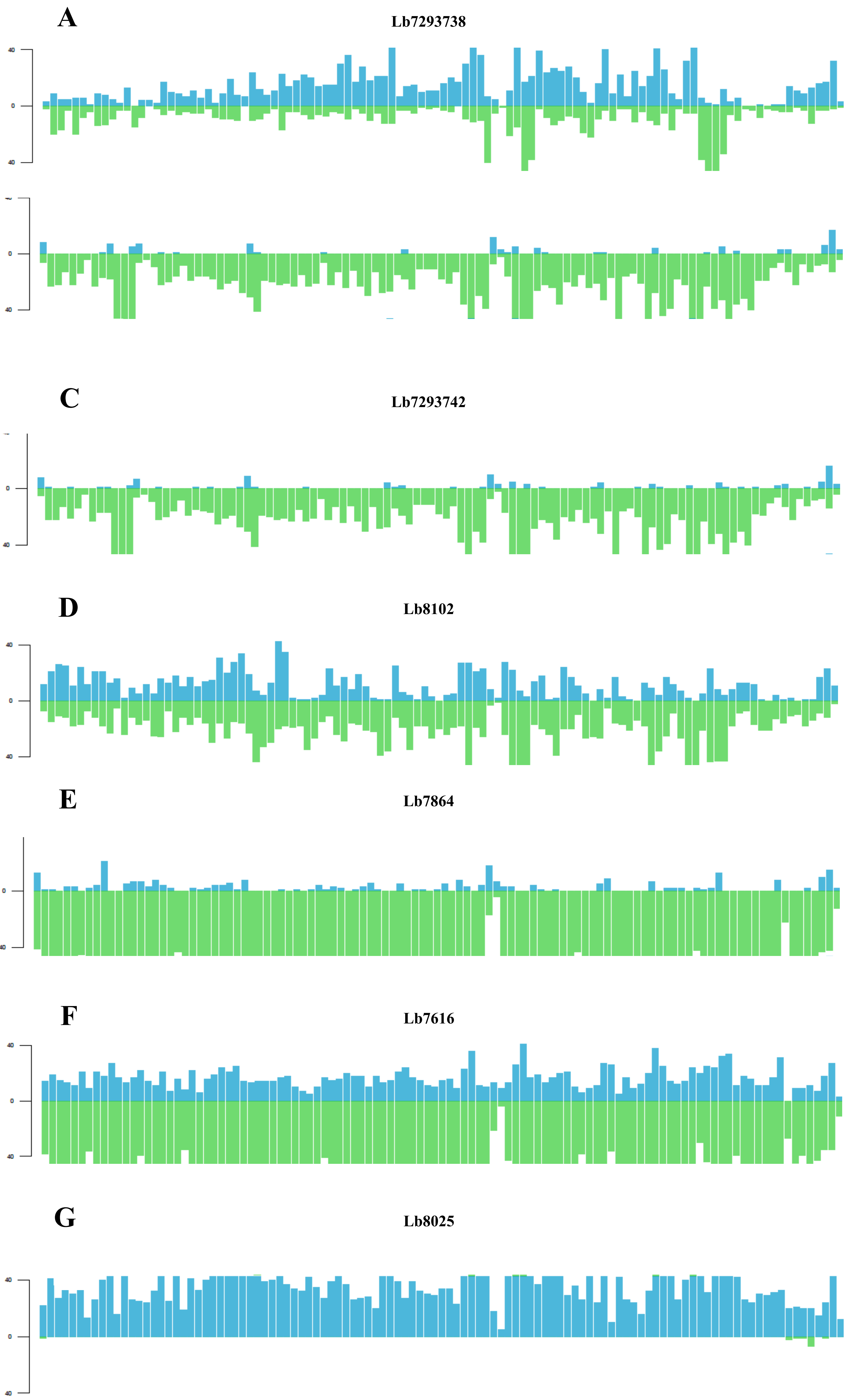

Chr-28

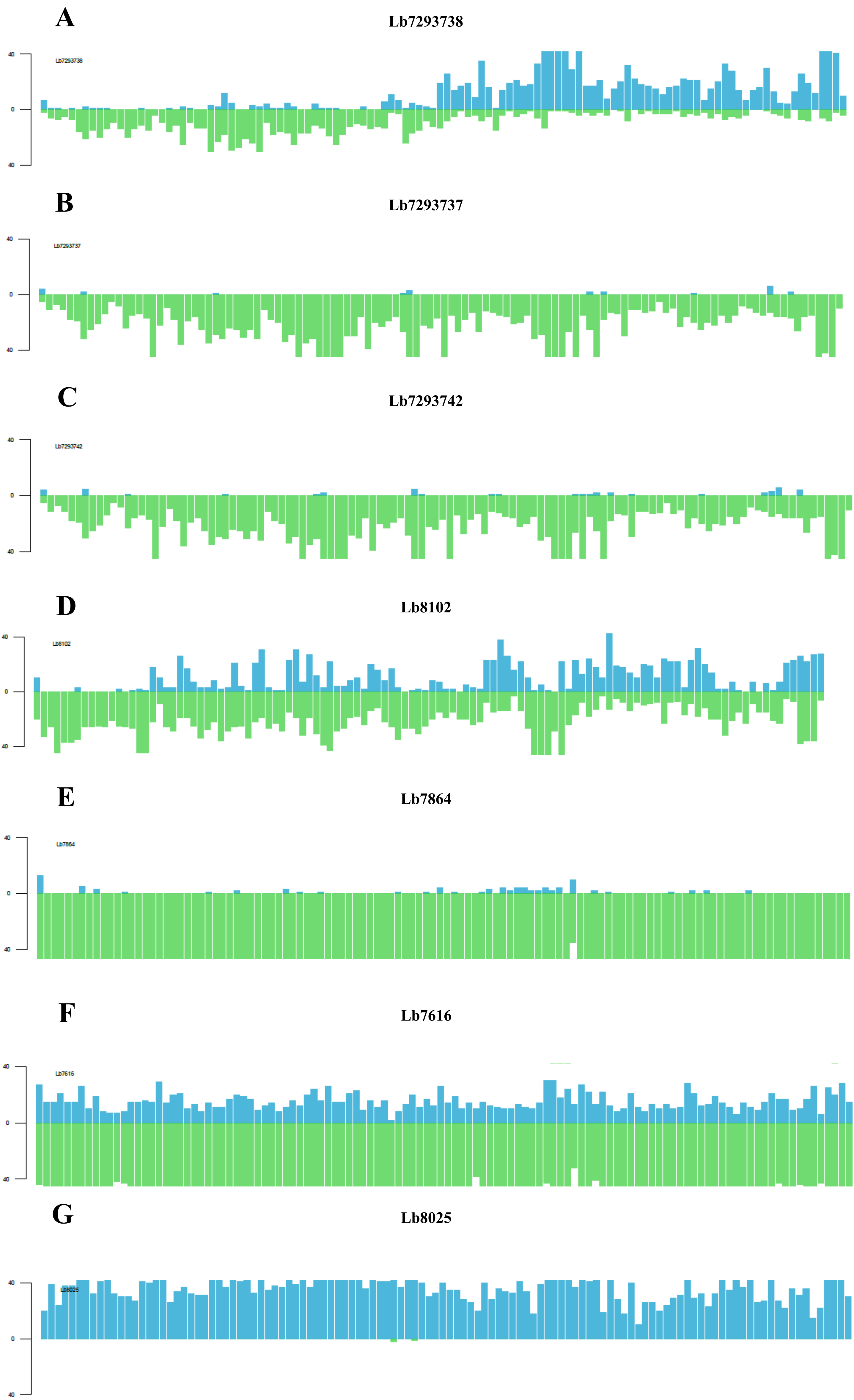
A
Lb7293738
40
0
40
B
Lb7293737
40
0
40
C
Lb7293742
40
0
40
D
Lb8102
40
0
40
E
Lb7864
40
0
40
F
Lb7616
40
0
40
G
Lb8025
40
0
40

Chr-29

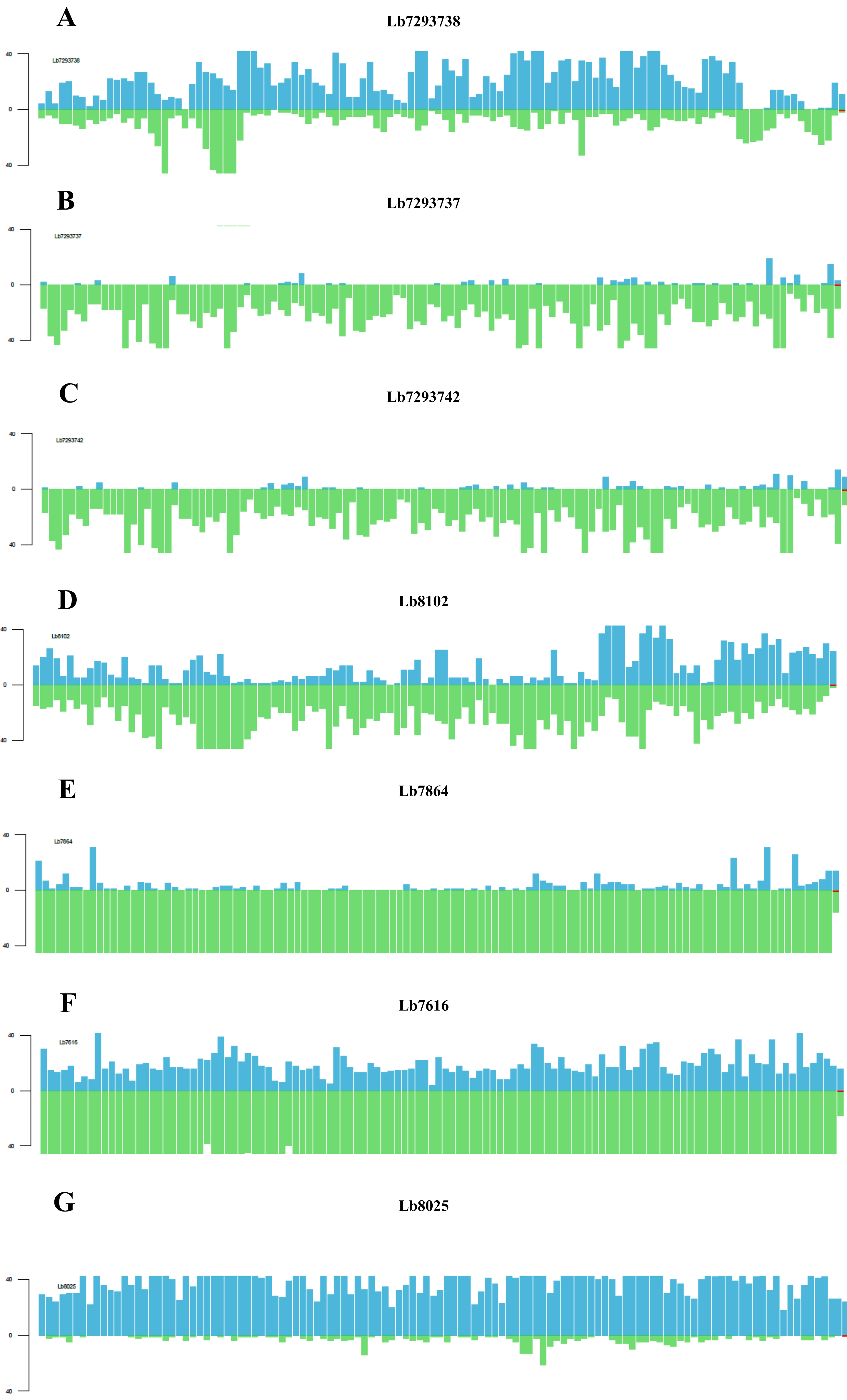
A
Lb7293738
B
Lb7293737
C
Lb7293742
D
Lb8102
E
Lb7864
F
Lb7616
G
Lb8025

Chr-30

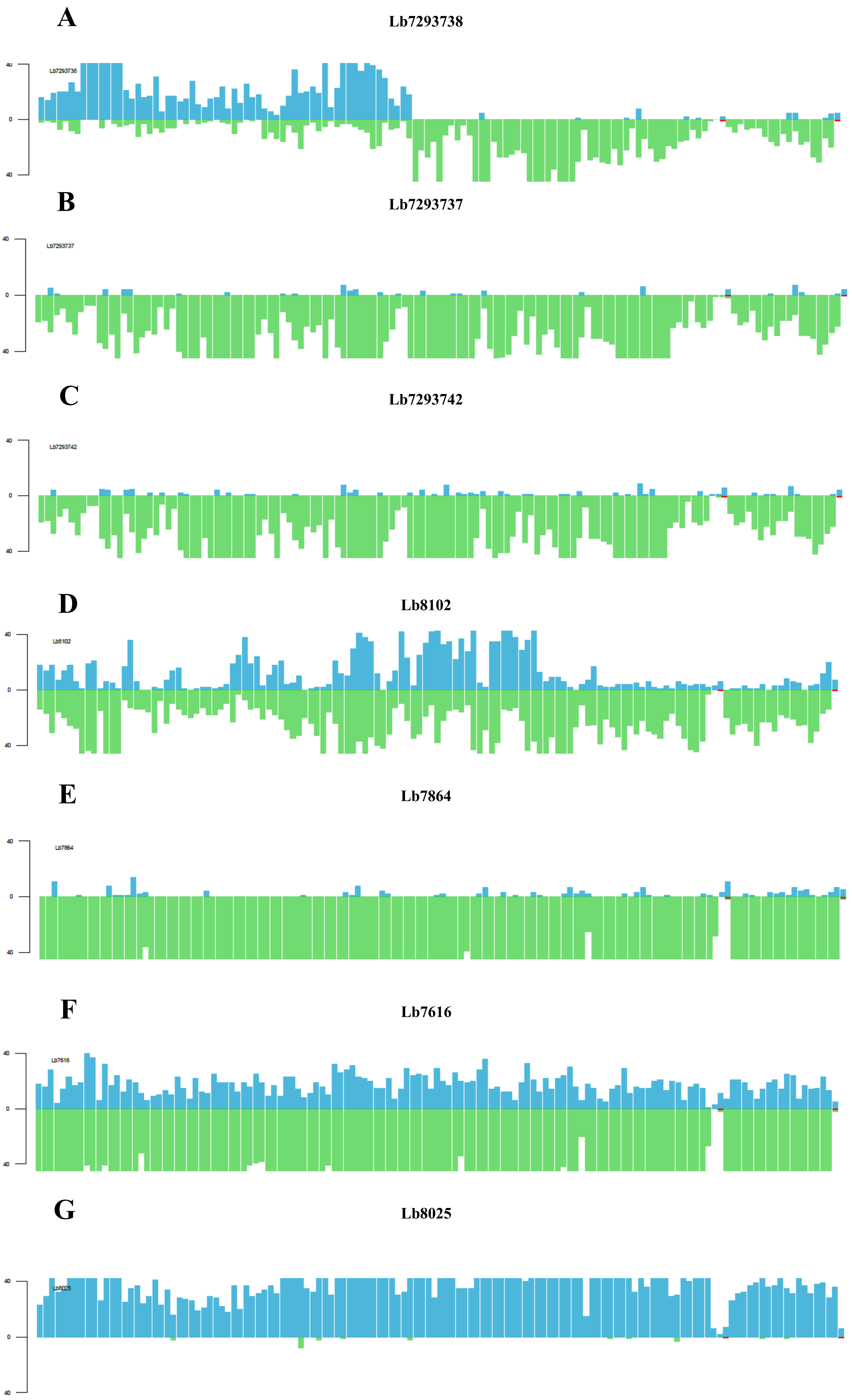
A
Lb7293738
B
Lb7293737
C
Lb7293742
D
Lb8102
E
Lb7864
F
Lb7616
G
Lb8025
40
0
40

Chr-31

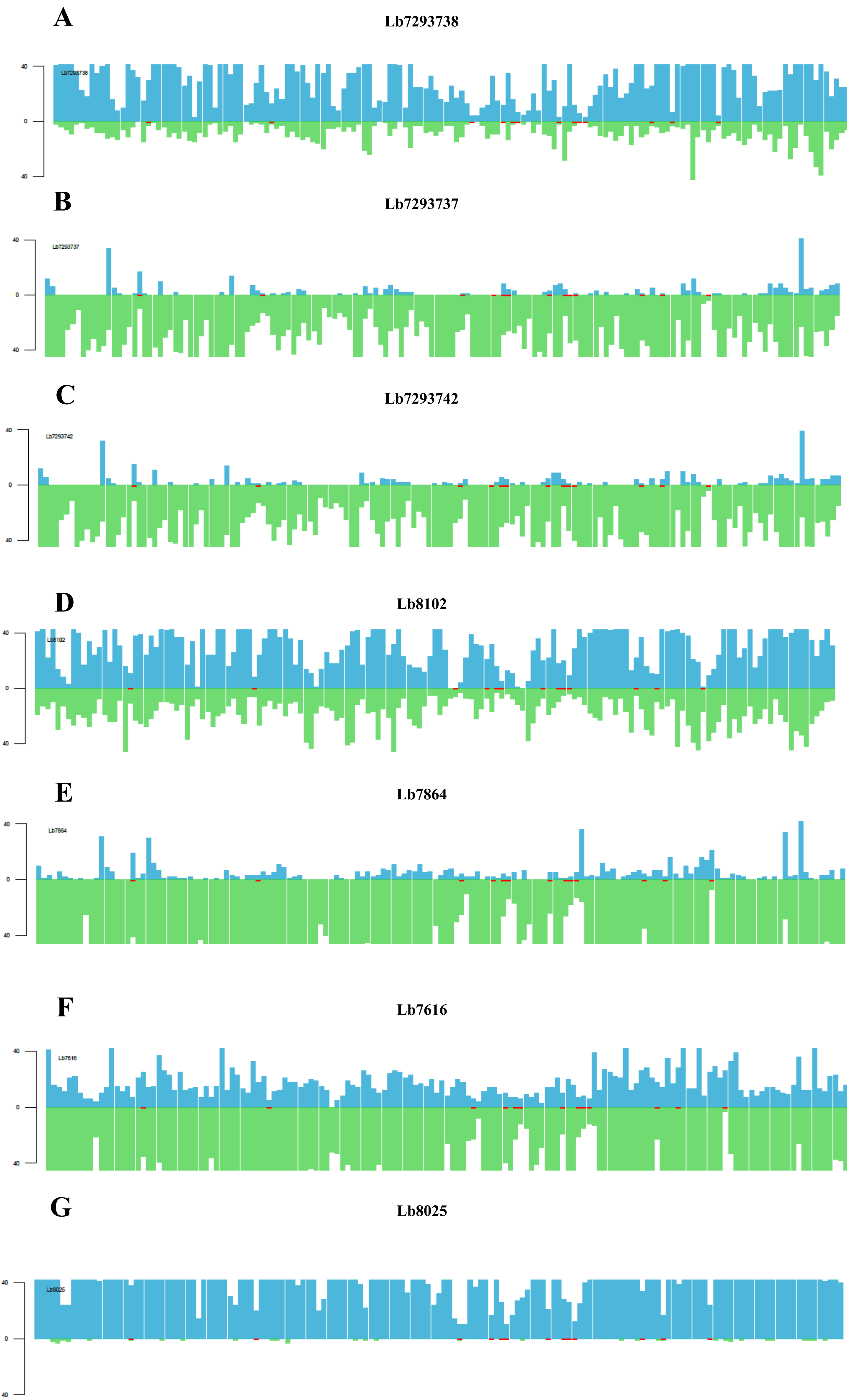
A
Lb7293738
B
Lb7293737
C
Lb7293742
D
Lb8102
E
Lb7864
F
Lb7616
G
Lb8025
40
0
40

Chr-32

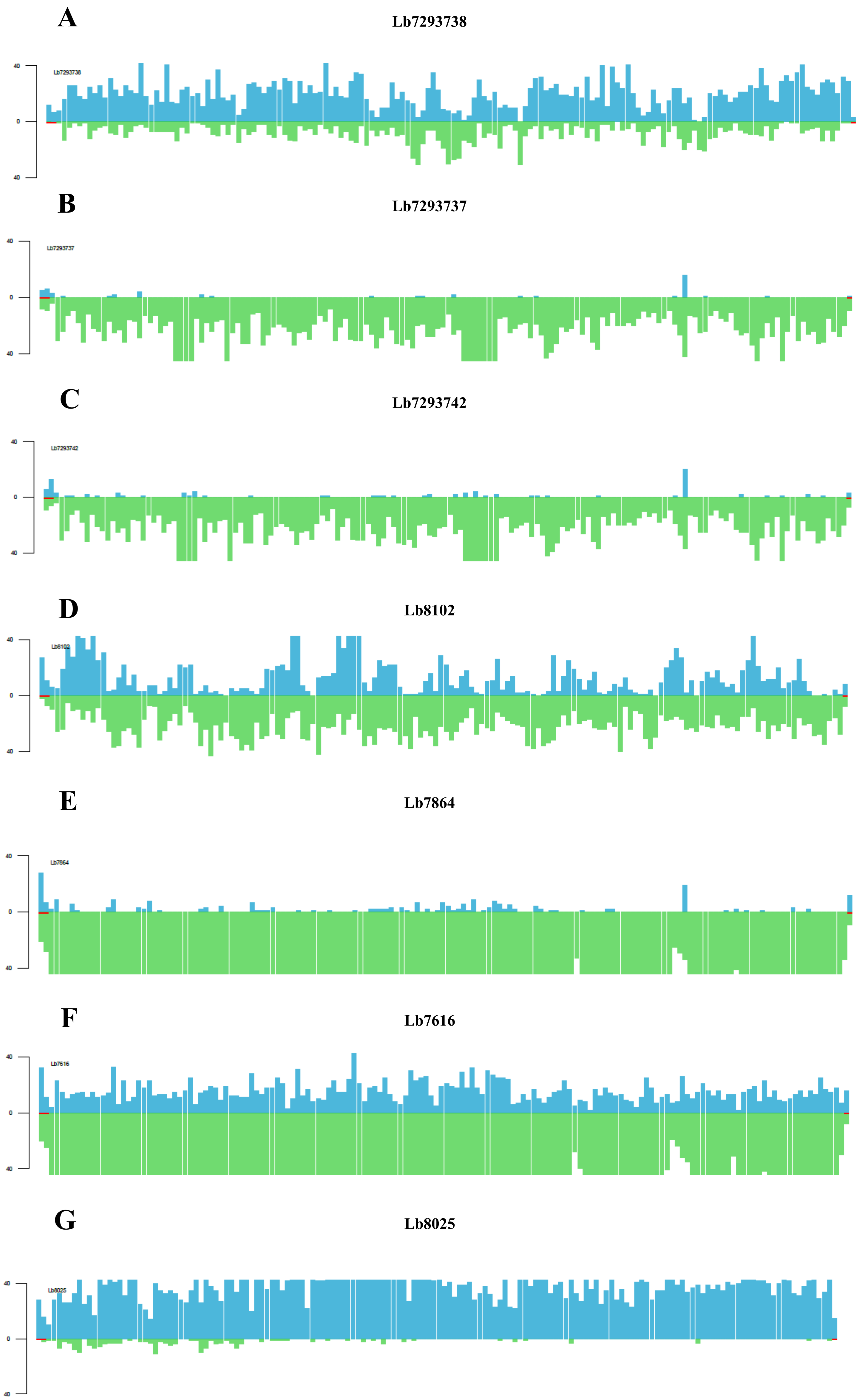
A
Lb7293738
40
0
40
B
Lb7293737
C
Lb7293742
D
Lb8102
E
Lb7864
F
Lb7616
G
Lb8025

Chr-33

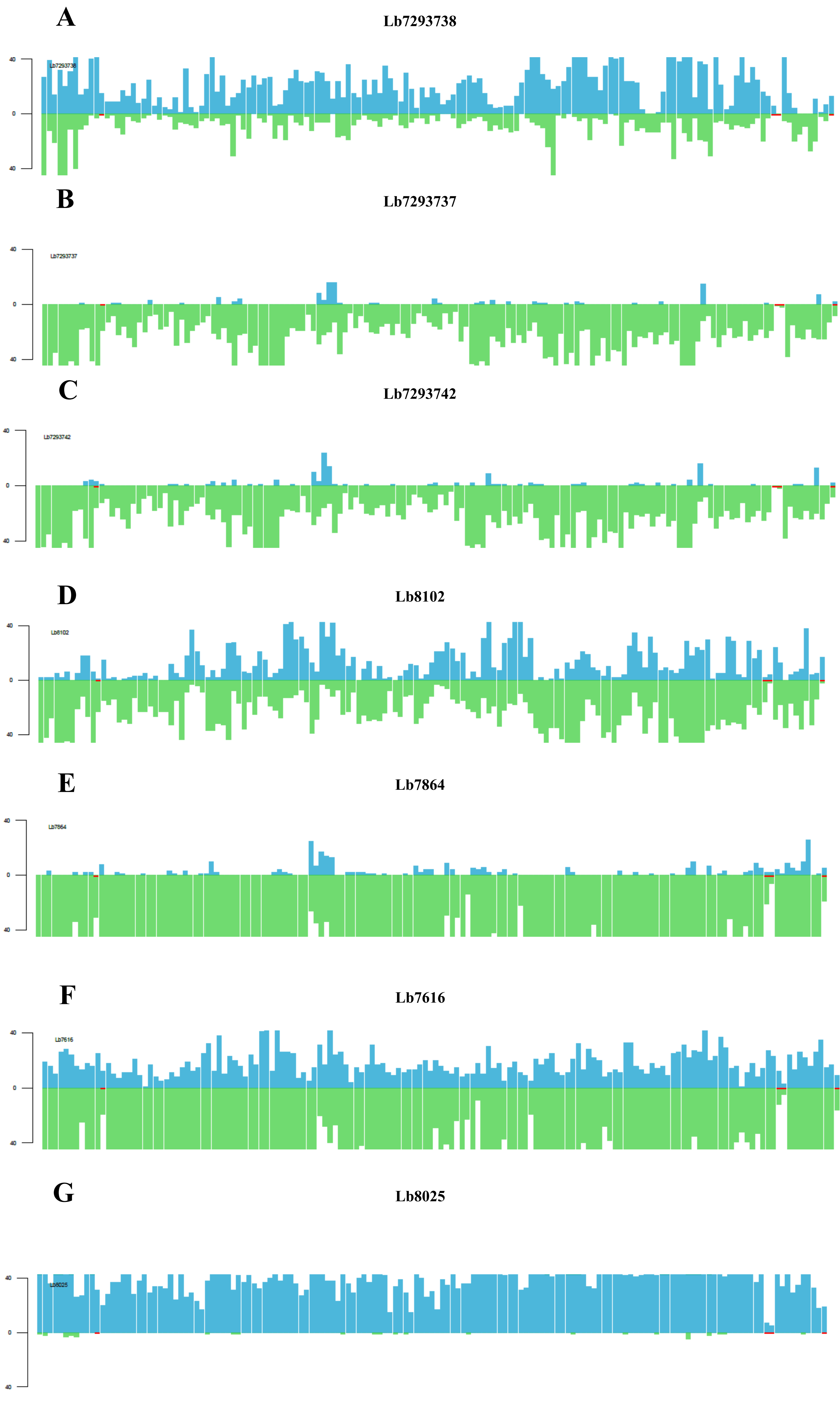
A
Lb7293738
B
Lb7293737
C
Lb7293742
D
Lb8102
E
Lb7864
F
Lb7616
G
Lb8025

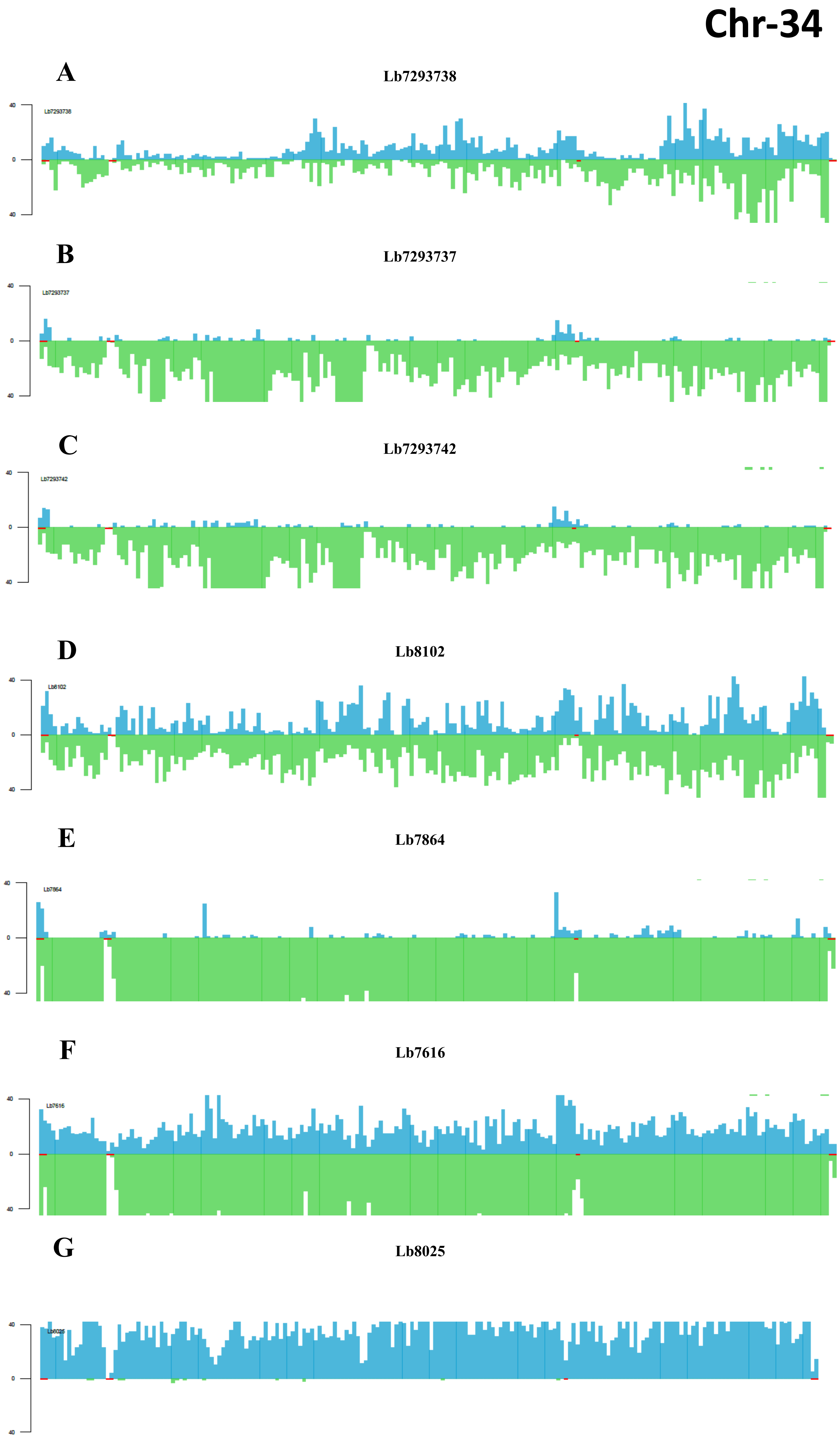
Chr-34
A
Lb7293738
B
Lb7293737
C
Lb7293742
D
Lb8102
E
Lb7864
F
Lb7616
G
Lb8025

Chr-35

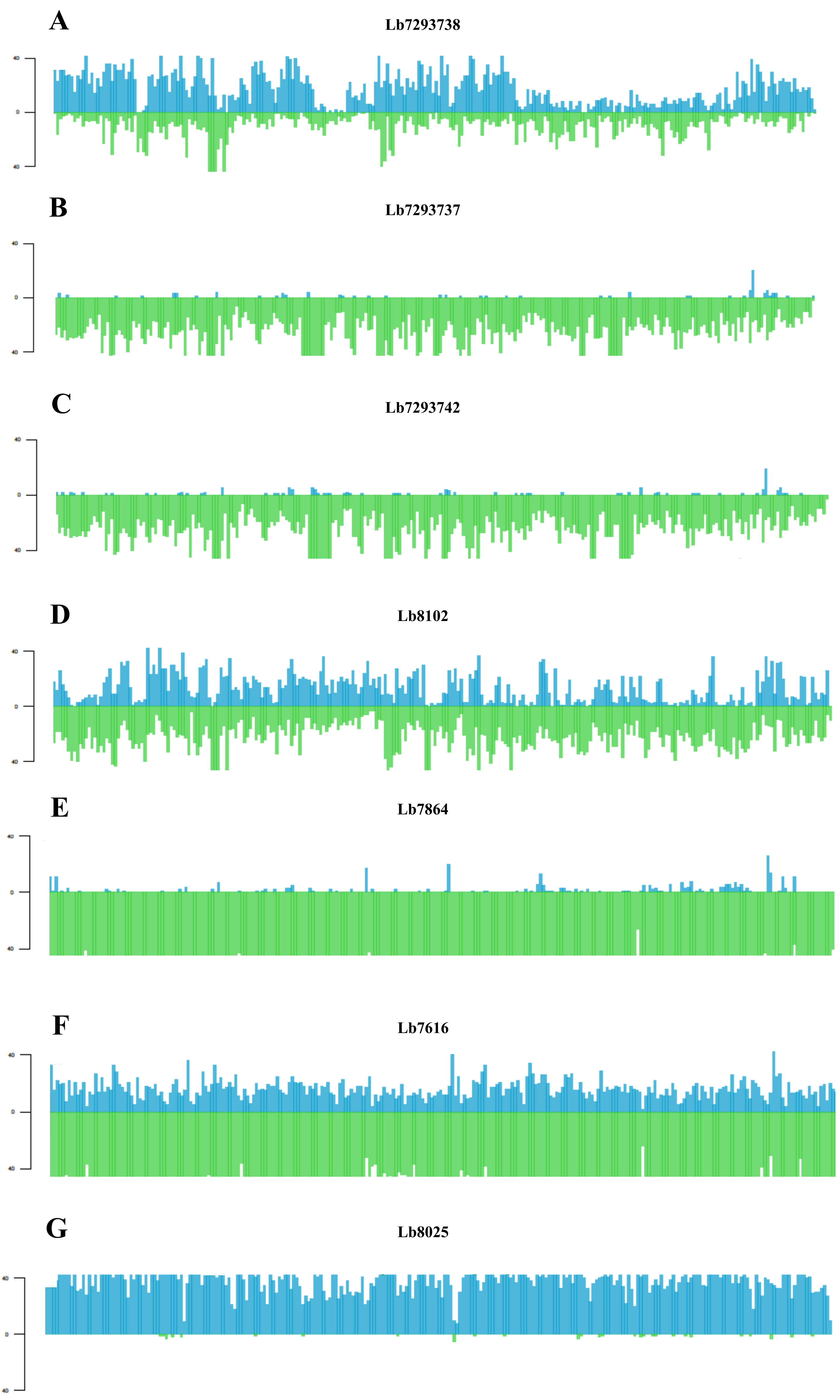
A
Lb7293738
40
0
40
B
Lb7293737
40
0
40
C
Lb7293742
40
0
40
D
Lb8102
40
0
40
E
Lb7864
40
0
40
F
Lb7616
40
0
40
G
Lb8025
40
0
40
